# Clinicopathologic and genomic features of lobular like invasive mammary carcinoma: is it a distinct entity?

**DOI:** 10.1038/s41523-023-00566-7

**Published:** 2023-07-13

**Authors:** Jing Yu, Edaise M. da Silva, Hae-Sun La, Beth Z. Clark, Jeffrey L. Fine, Gloria J. Carter, Tatiana M. Villatoro, T. Rinda Soong, Adrian V. Lee, Steffi Oesterreich, Thais Basili, Juan Blanco-Heredia, Pier Selenica, Qiqi Ye, Arnaud Da Cruz Paula, Higinio Dopeso, Andrea Gazzo, Antonio Marra, Fresia Pareja, Jorge S. Reis-Filho, Rohit Bhargava

**Affiliations:** 1 Department of Pathology, University of Pittsburgh Medical Center Magee-Womens Hospital, Pittsburgh, PA USA; 2 Department of Pathology and Laboratory Medicine, Memorial Sloan Kettering Cancer Center, New York, NY USA; 3 Department of Pharmacology and Chemical Biology, University of Pittsburgh, UPMC Hillman Cancer Center, Pittsburgh, PA USA

**Keywords:** Breast cancer, Breast cancer

## Abstract

This study describes “lobular-like invasive mammary carcinomas” (LLIMCas), a group of low- to intermediate-grade invasive mammary carcinomas with discohesive, diffusely infiltrative cells showing retained circumferential membranous immunoreactivity for both E-cadherin and p120. We analyzed the clinical-pathologic features of 166 LLIMCas compared to 104 classical invasive lobular carcinomas (ILCs) and 100 grade 1 and 2 invasive ductal carcinomas (IDCs). Tumor size and pT stage of LLIMCas were intermediate between IDCs and ILCs, and yet often underestimated on imaging and showed frequent positive margins on the first resection. Despite histomorphologic similarities to classical ILC, the discohesion in LLIMCa was independent of E-cadherin/p120 immunophenotypic alteration. An exploratory, hypothesis-generating analysis of the genomic features of 14 randomly selected LLIMCas and classical ILCs (7 from each category) was performed utilizing an FDA-authorized targeted capture sequencing assay (MSK-IMPACT). None of the seven LLIMCas harbored *CDH1* loss-of-function mutations, and none of the *CDH1* alterations detected in two of the LLIMCas was pathogenic. In contrast, all seven ILCs harbored *CDH1* loss-of-function mutations coupled with the loss of heterozygosity of the *CDH1* wild-type allele. Four of the six evaluable LLIMCas were positive for *CDH1* promoter methylation, which may partially explain the single-cell infiltrative morphology seen in LLIMCa. Further studies are warranted to better define the molecular basis of the discohesive cellular morphology in LLIMCa. Until more data becomes available, identifying LLIMCas and distinguishing them from typical IDCs and ILCs would be justified. In patients with LLIMCas, preoperative MRI should be entertained to guide surgical management.

## Introduction

Invasive mammary carcinoma of no special type, commonly referred to as invasive ductal carcinoma (IDC), and the special subtype of invasive lobular carcinoma (ILC) represent the two most common types of invasive breast carcinomas^1^. While IDCs typically show varying degrees of duct formation, ILCs are characterized by discohesive tumor cells with single-file infiltrative growth patterns dispersed in the fibrous stroma^2^. The differences between IDC and ILC, from clinicopathological features to prognostic outcomes, have been extensively reported in the literature, sometimes with conflicting results^3–5^. More recently, attention has turned to the molecular and evolutionary differences between the two entities and their precursor lesions, laying the foundations for personalized management of breast cancers^6–10^. Even though the diagnosis of IDC versus ILC is usually straightforward, cases with ambiguous histomorphology are not uncommon. Immunohistochemical (IHC) assessment of E-cadherin with or without p120 and beta-catenin is often used to assist in the diagnosis of such cases.

E-cadherin is a transmembrane adhesion glycoprotein encoded by the *CDH1* gene (16q22.1). P120 catenin is a tyrosine kinase substrate anchored to the internal domain of E-cadherin in a juxtamembranous fashion^11–13^. Characteristically, ILC harbors biallelic inactivation of the *CDH1* gene, often through a combination of pathogenic loss-of-function mutations coupled with loss-of-heterozygosity (LOH) of the *CDH1* wild-type allele^7,14^. When E-cadherin is absent or nonfunctional, p120 catenin undergoes redistribution from the cell membrane to the cytoplasm. Although practice patterns among subspecialists in breast pathology are not uniform, conventionally, lack of E-cadherin membranous expression coupled with diffuse, intense cytoplasmic p120 catenin expression is diagnostic of lobular lesions, whereas distinctively crisp, intense membranous positivity for both E-cadherin and p120 catenin is characteristic of ductal phenotype. In the past decade, emerging evidence has revealed that a non-functional E-cadherin might present as an aberrant (i.e., lack of strong membranous reactivity) yet visible pattern immunohistochemically^15–19^. Most reported cases with aberrant E-cadherin immunoreactivity, however, displayed a corresponding disruption of the cadherin-catenin complex^15,17–21^.

The 5th edition of the WHO classification of tumors for breast carcinoma does not recommend the use of immunohistochemistry (IHC) for the diagnosis of ILC^2^. However, in our experience, the interobserver agreement is limited in classifying ILC with ambiguous morphology without IHC. Findings from a recent study by Christgen et al.^22^ also supported our observation. In their study, 35 pathologists were asked to classify specimens (using 2 sets of cases—set A with H&E section only and set B with H&E and E-cadherin IHC) as non‐lobular breast carcinoma versus mixed breast carcinoma versus ILC. Pairwise interobserver agreement was moderate in set A (median κ = 0.58) and substantial in set B (median *κ* = 0.75, *p* < 0.001). Agreement with the reference diagnosis was substantial in set A (median *κ* = 0.67) and almost perfect in set B (median *κ* = 0.86, *p* < 0.001). The authors of this study concluded that subtyping of breast cancer as ILC achieves almost perfect agreement with a pre‐defined reference standard if the assessment is supported by E‐cadherin IHC. To improve the standardization of lobular carcinoma diagnosis, it has been our practice at Magee-Womens Hospital since 2004–2005 to confirm the first-time diagnosis of ILC by E-cadherin (along with p120) IHC staining. Either complete absence or “aberrant” reactivity for E-cadherin combined with intense cytoplasmic p120 catenin expression is required for a diagnosis of ILC. Reactivity for E-cadherin is considered “aberrant” if it is partially membranous, beaded, perinuclear dot-like, or cytoplasmic. In the event of an equivocal E-cadherin staining, concurrent cytoplasmic p120 staining supports the diagnosis of ILC, whereas circumferential membranous p120 reactivity is indicative of ductal immunophenotype. When different combinations of E-cadherin/p120 immunoprofile are present, a diagnosis of mixed IDC and ILC is justified. Our approach, although different from WHO recommendation, results in a more accurate classification and is the basis of this study.

We have observed in our daily practice a group of invasive carcinomas predominantly characterized by dissociated tumor cells with low-to-intermediate grade, uniform nuclei, and lobular-like growth patterns but display distinct membranous IHC staining for both E-cadherin and p120. We refer to such cases as “lobular-like invasive mammary carcinoma” (LLIMCa), but it is unclear whether this terminology is appropriate and if such cases harbor biallelic inactivation of *CDH1*, akin to ILCs. To our knowledge, no previous study has defined the clinical-pathologic characteristics or addressed the clinical behavior of LLIMCa, even though multiple prior studies, including those analyzing “IDC with lobular features”, in fact, examined mixed ductal and lobular carcinomas with heterogeneous E-cadherin expression^23–27^.

The current study aims to (1) analyze the clinical, radiological, and pathological characteristics and prognostic outcomes of LLIMCa, (2) compare the characteristics of LLIMCa with those of classical ILC and typical IDC of no special type, (3) assess representative LLIMCa cases for biallelic alterations of *CDH1*, and (4) perform an exploratory, hypothesis-generating analysis of the repertoire of somatic genetic alterations comparing representative LLIMCa and classical ILC cases.

## Results

### Morphologic and immunohistologic features

**Fig. 1:**
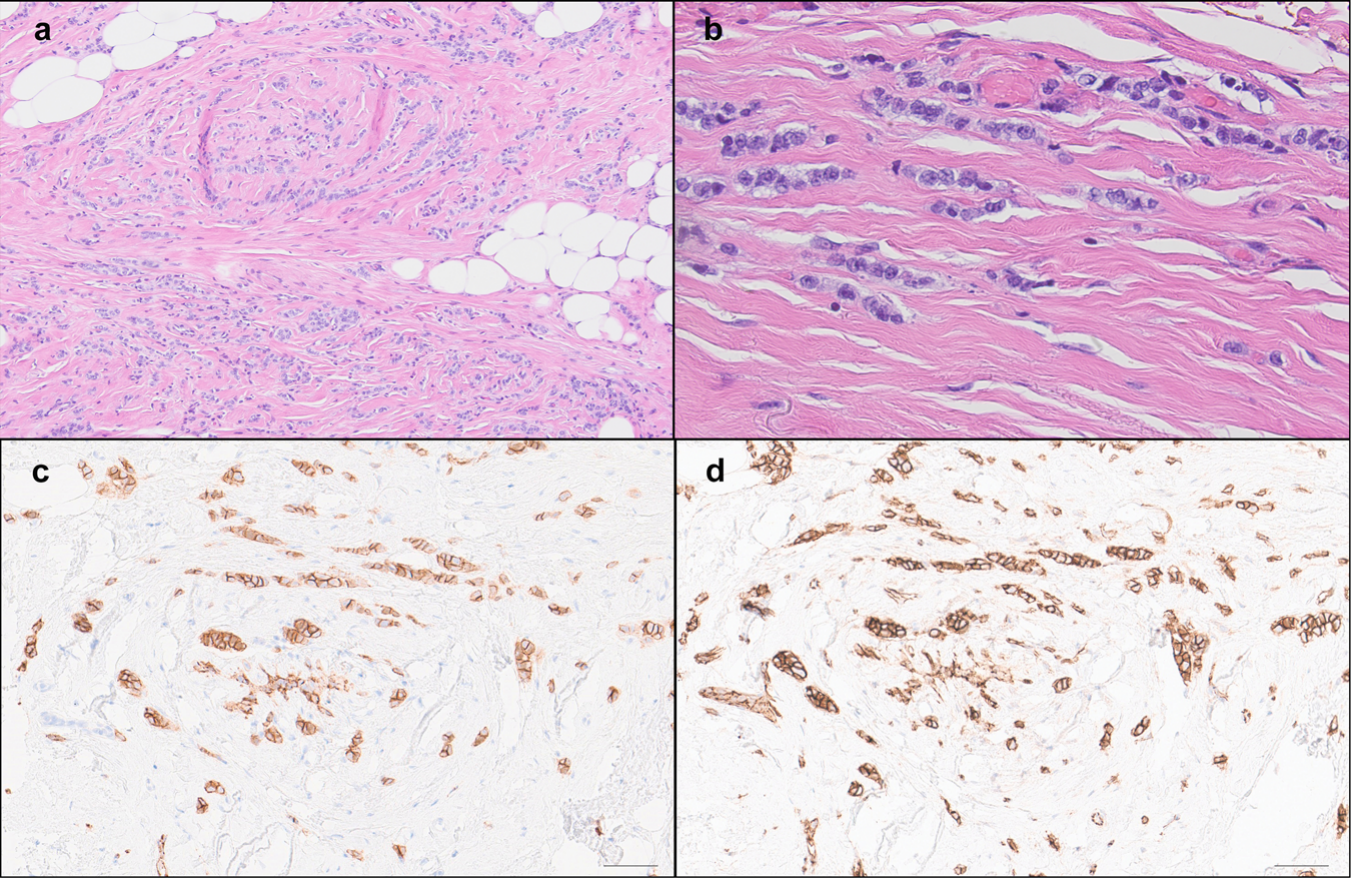
Histologic characteristics of lobular-like invasive mammary carcinoma (LLIMCa). LLIMCa shows individual cells in single files and cords within the fibrous stroma (**a** H&E, 100× and **b** H&E, 400×). The tumor cells show circumferential membranous staining for E-cadherin (**c** 200×) and p120 (**d** 200×). Scale bar = 100 µm.

**Fig. 2:**
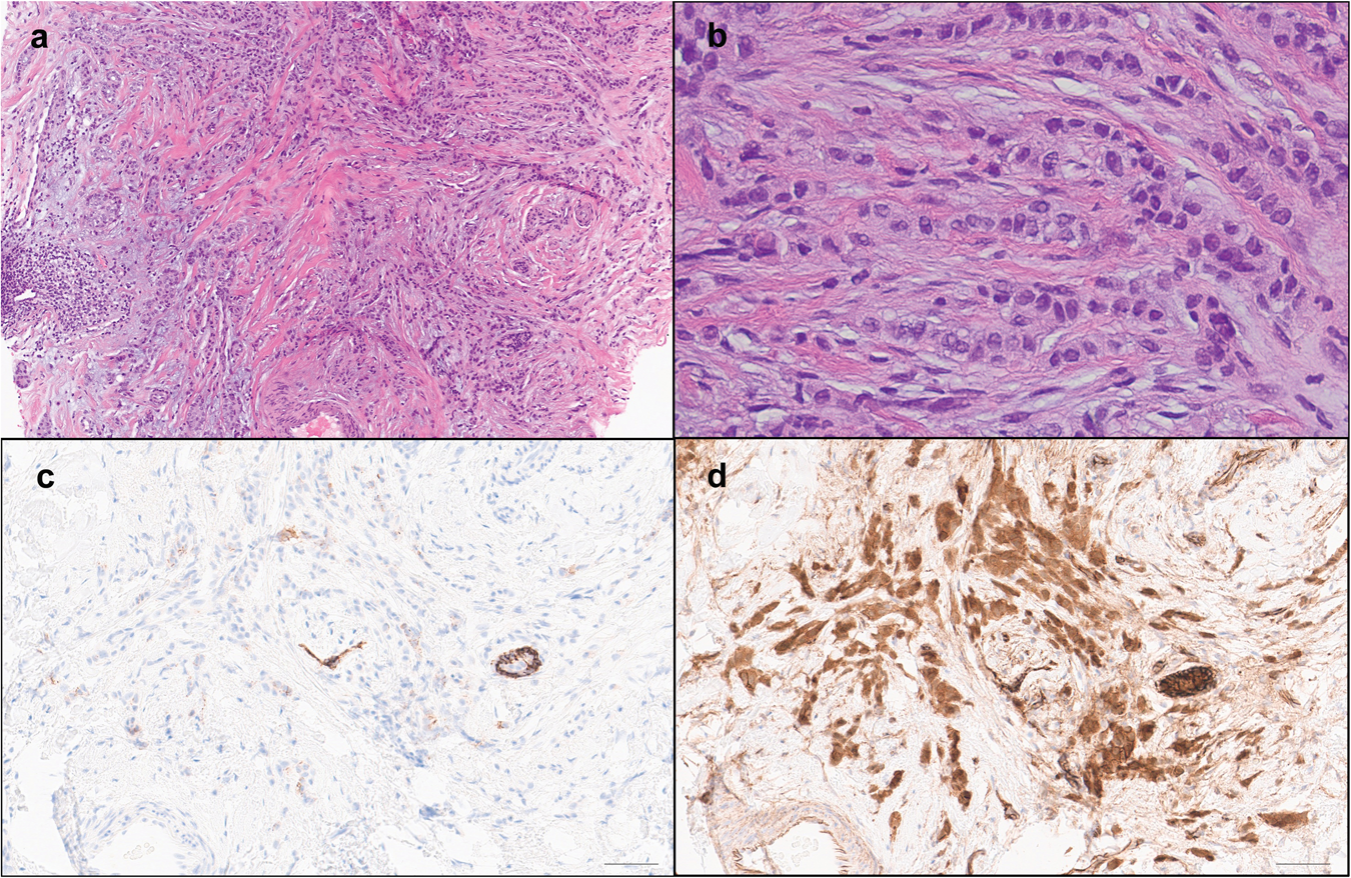
Histologic characteristics of invasive lobular carcinoma (ILC). ILC shows individual cells in single files and cords within the fibrous stroma (**a** H&E, 100× and **b** H&E, 400×). The tumor cells show aberrant partial membranous staining for E-cadherin (**c** 200×) and predominantly cytoplasmic reactivity for p120 (**d** 200×). Scale bar = 100 µm.

Consistent with the definition of LLIMCa we put forth (refer to Methods for details), this group of tumors was found to harbor predominantly discohesive, uniform, small- to intermediate-size nuclei and lobular-like dissociated growth pattern, presenting as individual cells in single-files and cords within the fibrous stroma. Neoplastic cells were occasionally arranged in a concentric “targetoid” pattern around benign ducts and lobules. Nested or trabecular patterns were infrequently observed. Cytologically the tumor cells tended to have slightly larger and more angulated nuclei than classical-type ILC. The histomorphologic features were reflected in the analyzed Nottingham scores. Signet ring cells were not prominent in any of the LLIMCa cases. Scattered intracytoplasmic vacuoles were seen in some cases of LLIMCa. Overall, LLIMCas were morphologically difficult or even impossible to classify definitively as either lobular or ductal on H&E sections alone (Fig. 1a, b). Immunohistochemically, all tumor cells demonstrated distinct and circumferential membranous expression of both E-cadherin and p120 catenin, characteristic of ductal phenotype (Fig. 1c, d). In contrast, ILCs were characterized by either complete loss or aberrant expression of E-cadherin, coupled with predominantly cytoplasmic p120 catenin (Fig. 2).

In addition, β-catenin stain was performed on all 14 cases (7 LLIMCas and 7 ILCs) randomly selected for the exploratory genomic analysis. All 7 cases of LLIMCa demonstrated membranous expression. All 7 cases of ILC revealed granular cytoplasmic uptake of β-catenin (Supplementary Figs. 1 and 2). A recent study identified a group of ILCs with N-terminal-deficient E-cadherin while preserving E-cadherin C-terminus^28^. To confirm whether LLIMCas in the present study were, in fact, ILCs with N-terminal-deficient yet C-terminal-conserved E-cadherin protein, we subjected the above 14 cases to IHC staining of antibodies against E-cadherin N-terminus. All 7 LLIMCas showed membranous staining, while all 7 ILCs were negative for N-terminal E-cadherin (Supplementary Fig. 3).

### Clinical characteristics

A comparison of the clinical characteristics of LLIMCa (*n* = 166), IDC (*n* = 100), and ILC (*n* = 104) (Table 1) revealed that patients with LLIMCa presented at a slightly older mean age (60.6 years) compared to those with IDC (57 years, *p* = 0.035), but were similar in age to ILC patients (61.6 years, *p* = 0.493). During the study period, LLIMCa patients more often (81%) underwent breast-conserving segmental resection as the first surgical procedure, compared to ILC (63%, *p* = 0.003) and IDC (52%, *p* < 0.0001) patients. In contrast, a significantly higher number of LLIMCa (83%, *p* < 0.0001) and ILC (75%, *p* < 0.0001) patients received adjuvant radiation therapy compared to IDC patients (47%), who had a significantly higher number of total mastectomies and avoided subsequent radiation. There were no differences in adjuvant systemic endocrine therapy or chemotherapy among the three tumor types. Albeit not statistically significant, the proportions of patients with LLIMCa developing locoregional recurrence appeared to be intermediate between ILC and IDC. In contrast, ILC was associated with a significantly higher frequency of locoregional recurrence compared to IDC. No difference in the patterns of distant metastasis was observed between different tumor types in our study.

**Table 1:** Comparison of clinical characteristics.

	LLIMCa (*n* = 166)	ILC (*n* = 104)	IDC (*n* = 100)	LLIMCa vs ILC (*P*-value)	LLIMCa vs IDC (*P*-value)	ILC vs IDC (*P*-value)
*Age (years)*						
Mean	60.6	61.6	57	0.493	0.035*	0.012*
Range	31–89	43–85	26–85			
*Procedure*						
Segmental	135 (81%)	65 (63%)	52 (52%)	0.003*	<0.0001*	0.002*
Mastectomy	31 (19%)	39 (37%)	48 (48%)			
*Radiation treatment*						
No	29 (17%)	25 (24%)	53 (53%)	0.210	<0.0001*	<0.0001*
Yes	137 (83%)	78 (75%)	47 (47%)			
Unknown	0 (0%)	1 (1%)	0 (0%)			
*Systemic therapy*						
Endo only	95 (57%)	59 (57%)	68 (68%)	0.135	0.208	0.122
Chemo + endo	67 (40%)	39 (37%)	30 (30%)			
Chemo only	1 (1%)	0 (0%)	1 (1%)			
None	0 (0%)	3 (3%)	0 (0%)			
Unknown	3 (2%)	3 (3%)	1 (1%)			
*Recurrence*						
No	146 (88%)	85 (82%)	93 (93%)	0.160	0.214	0.020*
Yes	20 (12%)	19 (18%)	7 (7%)			
*Recurrence type*						
No recurrence	146 (88%)	85 (82%)	93 (93%)	0.391	0.341	0.049*
Loco-regional	2 (1%)	2 (2%)	2 (2%)			
Distant	18 (11%)	17 (16%)	5 (5%)			
*Pattern of metastases*						
No metastases	148 (89%)	87 (84%)	96 (96%)	0.453	0.286	0.066
Ductal-like	9 (5%)	11 (10%)	4 (4%)			
Lobular-like	1 (1%)	1 (1%)	0 (0%)			
Neutral	8 (5%)	5 (5%)	1 (1%)			

*LLIMCa* lobular-like invasive mammary carcinoma, *ILC* invasive lobular carcinoma, *IDC* invasive ductal carcinoma. *Statistically significant, two-sided *t*-test is used to compare the age, and Chi-square and Fisher exact tests are used to compare other categories of clinical characteristics.

### Pathological characteristics

A comparison of the pathologic features of LLIMCa, ILC, and IDC (Table 2) demonstrated that the mean tumor size of LLIMCas (1.9 cm) was intermediate between IDCs (1.55 cm, *p* < 0.012) and ILCs (2.7 cm, *p* < 0.0001). As a result, LLIMCas presented at a higher pT stage than IDCs but a lower pT stage than ILCs.

**Table 2:** Comparison of pathological characteristics.

	LLIMCa (*n* = 166)	ILC (*n* = 104)	IDC (*n* = 100)	LLIMCa vs ILC (*P*-value)	LLIMCa vs IDC (*P*-value)	ILC vs IDC (*P*-value)
*Multifocality*						
No	138 (83%)	83 (80%)	78 (78%)	0.519	0.332	0.864
Yes	28 (17%)	21 (20%)	22 (22%)			
*Tumor foci number*						
Mean	1.28	1.43	1.35	0.255	0.531	0.612
Range	1–8	1–11	1–8			
*Tumor size (cm)*						
Mean	1.9	2.7	1.55	<0.0001*	0.012*	<0.0001*
Range	0.4–10	0.5–16	0.4–4			
*Tumor grade*						
I	11 (7%)	19 (18%)	46 (46%)	0.005*	<0.0001*	<0.0001*
II	155 (93%)	85 (82%)	54 (54%)			
*Nottingham score*						
3	0 (0%)	0 (0%)	5 (5%)	0.005*	<0.0001*	<0.0001*
4	0 (0%)	0 (0%)	11 (11%)			
5	11 (7%)	19 (18%)	30 (30%)			
6	140 (84%)	81 (78%)	49 (49%)			
7	15 (9%)	4 (4%)	5 (5%)			
*Calcifications*						
Absent	112 (67%)	75 (72%)	65 (65%)	0.155	0.589	0.061
Present	51 (31%)	22 (21%)	35 (35%)			
Unknown	3 (2%)	7 (7%)	0 (0%)			
*LVSI*						
No	122 (73%)	93 (89%)	78 (78%)	0.002*	0.465	0.036*
Yes	44 (27%)	11 (11%)	22 (22%)			
*Margin (1st surg)*						
Negative	104 (63%)	67 (65%)	81 (81%)	0.261	0.003*	0.002*
Close	42 (25%)	19 (18%)	16 (16%)			
Positive	20 (12%)	18 (17%)	3 (3%)			
*LN status*						
Negative	106 (64%)	64 (61%)	69 (69%)	0.590	0.579	0.292
Positive	51 (31%)	36 (35%)	25 (25%)			
Not available	9 (5%)	4 (4%)	3 (3%)			
*pT stage*						
1	111 (67%)	56 (54%)	82 (82%)	0.001*	0.016*	<0.0001*
2	51 (31%)	34 (33%)	18 (18%)			
3	4 (2%)	14 (13%)	0 (0%)			
*pN stage*						
0	106 (64%)	64 (62%)	69 (69%)	0.850	0.444	0.239
1	41 (25%)	27 (26%)	25 (25%)			
2	6 (4%)	6 (6%)	3 (3%)			
3	4 (2%)	3 (3%)	0 (0%)			
X	9 (5%)	4 (3%)	3 (3%)			

*LLIMCa* lobular-like invasive mammary carcinoma, *ILC* invasive lobular carcinoma, *IDC* invasive ductal carcinoma, *LVSI* lympho-vascular space invasion, *LN* lymph node, *pT* pathologic tumor stage, *pN* pathologic nodal stage. *Statistically significant, two-sided *t*-test is used to compare the tumor foci number and tumor size, and Chi-square and Fisher exact tests are used to compare the other pathological variables.

LLIMCas was more likely to have positive or close margins (37%) in the first surgical procedure compared to IDCs (19%, *p* = 0.003) but was similar to ILCs (35%, *p* = 0.261). LLIMCas were found to have a significantly higher rate of lymphovascular space invasion (27%) than ILCs (11%, *p* = 0.002) but were similar to IDCs (22%, *p* = 0.465). No differences in multifocality, number of tumor foci, tumor-associated calcifications, lymph node status, or pN stage were observed between any of the tumor types.

Within the group of nuclear grade 1 and 2 tumors, as designed in the current study, LLIMCas exhibited a higher Nottingham grade and score than IDCs and ILCs. A substantial number of IDC were Nottingham grade I (grade I: 46%, grade II: 54%) due to the presence of tubule formation, whereas LLIMCas (grade I: 7%, grade II: 93%, *p* < 0.0001) and ILCs (grade I: 18%, grade II: 82%, *p* < 0.0001) were more likely to be grade II due to the lack of tubules. Additionally, LLIMCas frequently had higher Nottingham grades and scores compared to ILCs (*p* = 0.005), owing to the larger nuclei and more frequent mitoses.

### Pathological–radiological correlation of tumor size

Analysis of the pathologic and radiologic tumor sizes at the time of diagnosis (Table 3) revealed that LLIMCas had a similar mean tumor size (1.35 cm) as IDCs (1.5 cm, *p* = 0.202) on radiologic measurement, which was smaller than that of ILCs (1.7 cm, *p* = 0.01). The final tumor size of LLIMCas (1.9 cm) upon pathologic examination of the resection specimen was intermediate between that of IDCs (1.55 cm, *p* = 0.012) and ILCs (2.7 cm, *p* < 0.0001). The majority of both LLIMCas (71%) and ILCs (75%) revealed larger pathologic tumor size compared to radiologic tumor size, whereas IDCs showed an even distribution of pathologic tumor size rather consistent with radiologic size. As a result, the pathologic to radiologic tumor size ratio of LLIMCas (1.6) was similar to ILCs (1.8) but significantly higher than that of IDC (1.1, *p* < 0.0001), indicating that the pathologic tumor size of either LLIMCas or ILCs was much larger than the tumor size estimated by imaging.

**Table 3:** Pathology–radiology correlation of tumor size.

	LLIMCa (*n* = 166)	ILC (*n* = 104)	IDC (*n* = 100)	LLIMCa vs ILC (*P*-value)	LLIMCa vs IDC (*P*-value)	ILC vs IDC (*P*-value)
*Pathology size (cm)*						
Mean	1.9	2.7	1.55	<0.0001*	0.012*	<0.0001*
Range	0.4–10	0.5–16	0.4–4			
*Radiology size (cm)*						
Mean	1.35	1.7	1.5	0.010*	0.202	0.119
Range	0–8.6	0.4–8	0.4–3.5			
*Path:Rad size* *>* *1*						
No	34 (21%)	20 (19%)	47 (47%)	0.755	<0.0001*	<0.0001*
Yes	118 (71%)	78 (75%)	50 (50%)			
Not available	14 (8%)	6 (6%)	3 (3%)			
*Path:Rad size ratio*						
Mean	1.6	1.8	1.1	0.255	<0.0001*	<0.0001*
Range	0.4–8.8	0.2–8	0.6–1.6			

*LLIMCa* lobular-like invasive mammary carcinoma, *ILC* invasive lobular carcinoma, *IDC* invasive ductal carcinoma. *Statistically significant, two-sided *t*-test is used to compare pathology size, radiology size, and Path:Rad size ratio, Fisher exact test is used to compare Path:Rad size > 1.

### Prognostic biomarkers

LLIMCa displayed similar profiles of estrogen receptor (ER), progesterone receptor (PR), and Ki67 compared to ILC and IDC (Table 4), even though the mean ER H-score of LLIMCas (248) was slightly higher than that of ILCs (231, p = 0.046). LLIMCas appeared to show a higher rate of HER2 positivity (10%) compared to ILCs (1%, *p* = 0.004) and IDCs (1%, *p* = 0.004) in the study population. However, only a small percentage (3 of 16, 1.8%) of the HER2-positive LLIMCas were IHC 3+; the others were IHC 2+ and amplified by FISH with low HER2 copies but HER2/CEP17 ratio crossing the 2 cut-offs. The low HER2 positivity rate in IDC was attributed to the exclusion of nuclear grade 3 tumors. Both LLIMCas and ILCs demonstrated significantly higher Magee Equation 2 (ME2) scores compared to IDC (Table 4). The ME2 score distribution, however, was similar between LLIMCas and ILCs (33% of cases with a score <18 for both LLIMCas and ILCs compared to 50% of cases with a score <18 for IDCs).

**Table 4:** Tumor prognostic biomarkers and ME2 scores.

Variables	LLIMCa (*n* = 166)	ILC (*n* = 104)	IDC (*n* = 100)	LLIMCa vs ILC (*P*-value)	LLIMCa vs IDC (*P*-value)	ILC vs IDC (*P*-value)
*ER status*						
Negative	3 (2%)	3 (3%)	1 (1%)	0.679	1.0	0.622
Positive	163 (98%)	101 (97%)	99 (99%)			
*PR status*						
Negative	16 (10%)	13 (12%)	4 (4%)	0.545	1.0	0.041*
Positive	150 (90%)	91 (88%)	96 (96%)			
*HER2 status*						
Negative	150 (90%)	103 (99%)	99 (99%)	0.004*	0.004*	1.0
Positive	16 (10%)	1 (1%)	1 (1%)			
*ER H-score*						
Mean	248	231	245	0.046*	0.709	0.130
Range	0–300	0–300	0–300			
*PR H-score*						
Mean	124	121	137	0.801	0.270	0.244
Range	0–300	0–300	0–300			
*Ki-67 index*						
Mean	21	16	17	0.111	0.134	0.857
Range	3–75	1–75	1–60			
Available on:	*N* = 63	*N* = 54	*N* = 49			
*ME2 score*						
Mean	20.18	20.53	18.13	0.535	<0.0001*	<0.0001*
Range	12.45–37.08	11.24–35.28	8.00–32.87			
*ME2 categories*						
Less than 18	55 (33%)	34 (33%)	50 (50%)	1.0	0.009*	0.015*
18 or more	111 (67%)	70 (67%)	50 (50%)			

*LLIMCa* lobular-like invasive mammary carcinoma, *ILC* invasive lobular carcinoma, *IDC* invasive ductal carcinoma, *ER* estrogen receptor, *PR* progesterone receptor, *ME2* Magee Equation 2. *Statistically significant, two-sided Fisher exact test is used to compare ER status, PR status, HER2 status, and ME2 categories, and *t*-test is used to compare ER *H*-score, PR *H*-score, Ki-67 index, and ME2 score.

### Long-term survival analysis

**Fig. 3:**
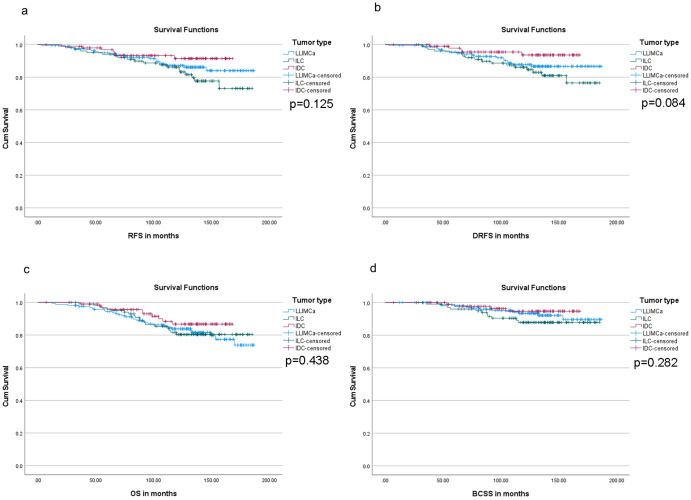
Kaplan Meier survival curves based on different histologic types. Recurrence-free survival or RFS (**a**), distant recurrence-free survival or DRFS (**b**), overall survival or OS (**c**), and breast cancer-specific survival or BCSS (**d**). Statistical significance is not reached based on different histologic types, p-values reported using log-rank test. LLIMCa lobular-like invasive mammary carcinoma, ILC invasive lobular carcinoma, IDC invasive ductal carcinoma.

**Fig. 4:**
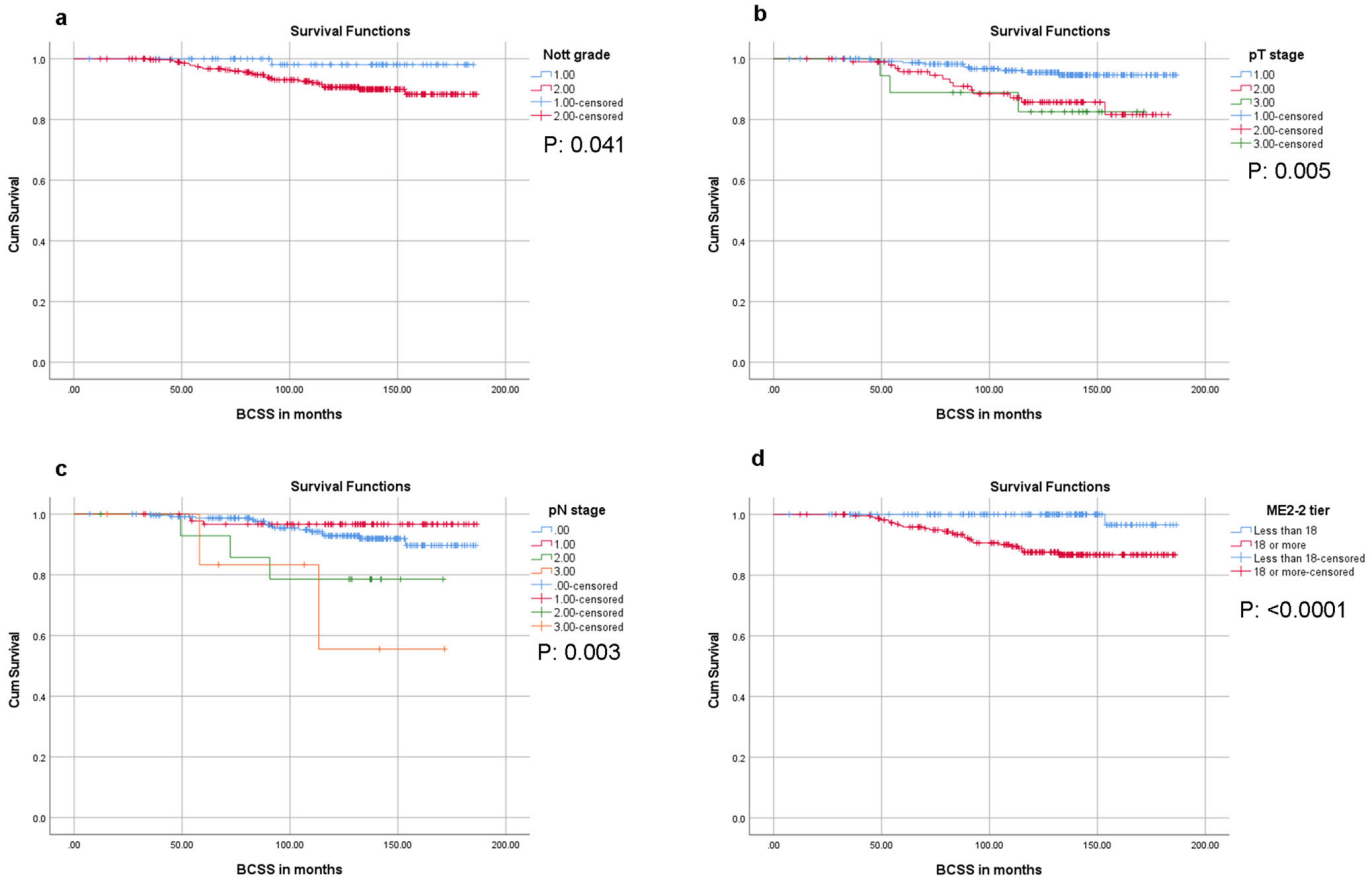
Breast cancer-specific survival (BCSS). Lower Nottingham grade (**a**), lower pathologic tumor (pT) stage (**b**), lower pathologic nodal (pN) stage (**c**), and lower Magee Equation 2 (ME2) score (**d**) were associated with significantly improved BCSS, *p*-values reported using the log-rank test.

For all three groups of patients, the median follow-up time for survival analysis was 10 years or longer (LLIMCa: 130 months, range 12.3–186.6 months; ILC: 128.7 months, range 28.8–185.1 months; IDC: 119.2, range 7.3–168.1 months). In the Kaplan–Meier survival analysis, although LLIMCas appeared to harbor an intermediate survival between IDCs and ILCs, statistical significance was not reached (Fig. 3) in either separate or combined sets of comparisons (Supplementary Data sets). Lower tumor grade (grade I), lower pT stage (pT1), lower pN stage (pN0 + pN1), and lower ME2 score (<18) were associated with significantly improved recurrence-free survival (RFS), distant RFS (DRFS), and breast cancer-specific survival (BCSS) (Table 5, Fig. 4 for BCSS, and Supplementary Data sets). Lower pT stage (pT1), lower pN stage (pN0 + pN1), and lower ME2 score (<18) were associated with significantly improved overall survival (OS) (Table 5 and Supplementary Data sets). Multivariable Cox proportional hazard regression analysis showed statistically significant improved RFS, DRFS, OS, and BCSS associated with lower pT stage (pT1) and lower ME2 score (<18) (Supplementary Data sets).

**Table 5:** Kaplan–Meier survival analysis log-rank test *p*-values for tumor types and known prognostic variables.

Variables	RFS	DRFS	OS	BCSS
ILC vs Others (IDC + LLIMCa)	0.074	0.071	0.736	0.148
IDC vs Others (ILC + LLIMCa)	0.113	0.059	0.202	0.248
Grade	0.007*	0.016*	0.080	0.041*
Nodal status	0.695	0.294	0.661	0.987
pT stage	0.002*	<0.0001*	0.014*	0.005*
pN stage	0.004*	0.001*	<0.0001*	0.003*
ME2 score	<0.0001*	0.003*	0.020*	<0.0001*

*Statistically significant. *ILC* invasive lobular carcinoma, *IDC* invasive ductal carcinoma, *LLIMCa* lobular-like invasive mammary carcinoma, *pT* pathologic tumor stage, *pN* pathologic nodal stage, *ME2* Magee Equation 2, *RFS* recurrence free-survival, *DRFS* distant recurrence-free survival, *OS* overall survival, *BCSS* breast cancer-specific survival.

### Somatic genetic alterations and mutation signatures

**Fig. 5:**
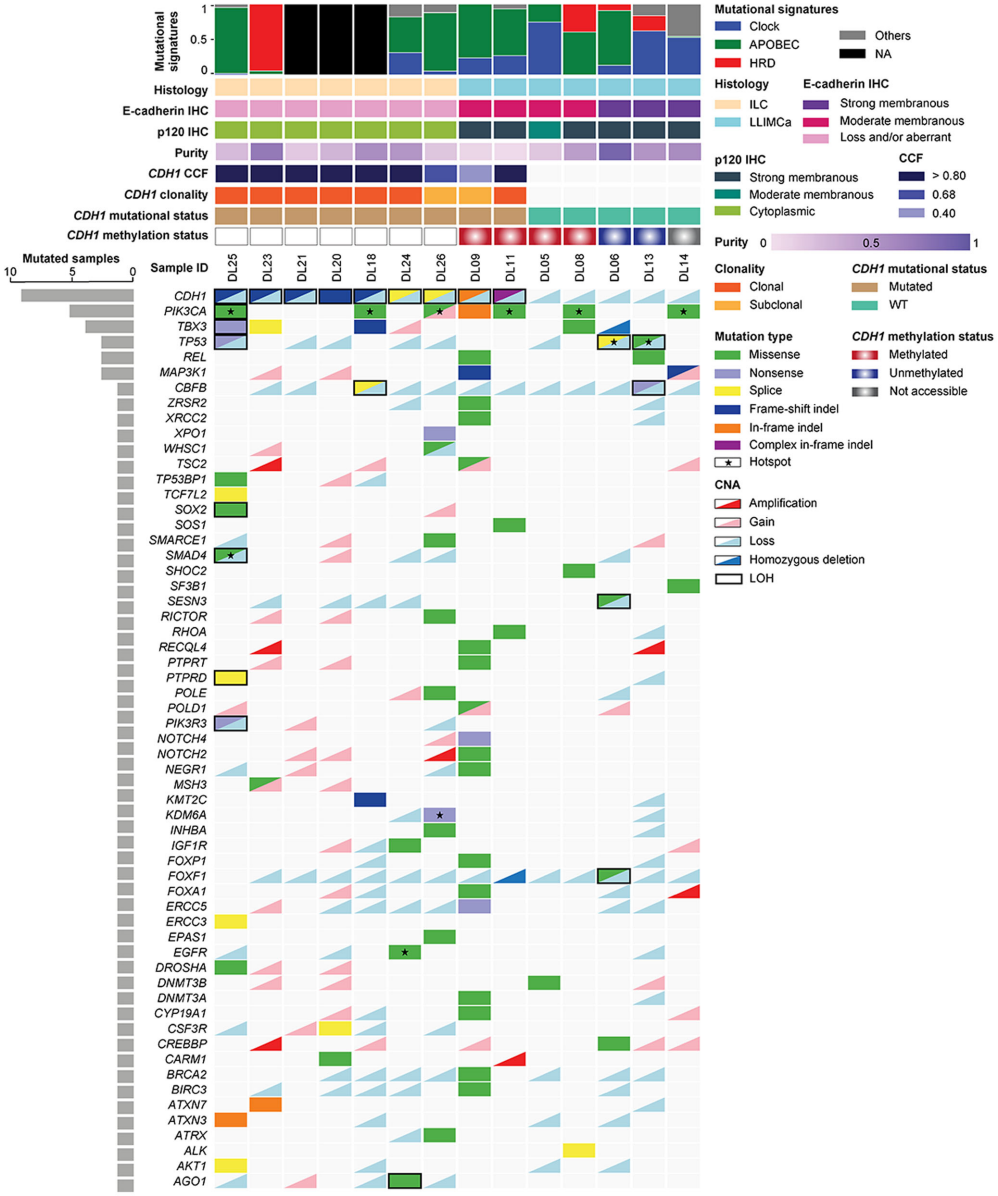
Repertoire of non-synonymous somatic mutations identified in invasive lobular carcinomas (ILC) and lobular-like invasive mammary carcinomas (LLIMCa) subjected to MSK-IMPACT. Non-synonymous somatic alterations identified in 7 ILCs and 7 LLIMCas detected by massively parallel sequencing targeting 515 cancer-related genes. Cases are shown in columns, and genes in rows. Clinicopathologic characteristics, including histology, E-cadherin and p120 staining by immunohistochemistry (IHC), tumor purity, *CDH1* cancer cell fraction (CCF), mutational clonality, and status are depicted in phenobars (top). Somatic mutation types and copy number alterations are color-coded according to the legend. Loss of heterozygosity (LOH) is depicted by a square. HRD homologous recombination DNA repair defect, NA not available, LOH loss of heterozygosity.

As an exploratory, hypothesis-generating analysis, we compared the repertoire of somatic genetic alterations of randomly selected 7 ILC and 7 LLIMCa cases from the current cohort (H&E and IHC stains shown in Supplementary Figs. 1 and 2) subjected to the Food and Drug Administration (FDA)-approved Memorial Sloan Kettering (MSK) Integrated Mutation Profiling of Actionable Cancer Targets (MSK-IMPACT) targeted sequencing assay (Fig. 5, Supplementary Table 1). Copy number analysis revealed 16q LOH in all cases (Fig. 5, Supplementary Table 2), consistent with their ER-positive status and lower Nottingham grades. In addition to 16q LOH, this analysis revealed that all bona fide ILCs harbored *CDH1* loss-of-function biallelic mutations (7/7), of which five were frameshift indel and two were splice site mutations uniformly coupled with LOH of the wild-type allele. Conversely, five of the seven LLIMCas did not harbor *CDH1* mutations or genomic rearrangements. *CDH1* alterations were identified in 2 LLIMCas: one (case DL09, Fig. 5) only harbored a subclonal *CDH1* in-frame indel mutation coupled with LOH. This case mostly displayed membranous E-cadherin and p120 expression with focal areas of aberrant expression (cytoplasmic E-cadherin instead of membranous reactivity, supplementary fig. 4), which was identified only after careful re-review following the genomic analyses. The other *CDH1-*mutated LLIMCa (case DL11, Fig. 5) harbored a complex in-frame indel (i.e., one intronic deletion, one splice site mutation (p.X441_splice) and a frameshift indel (p.D443Gfs*10) in cis) with negligible impact on protein structure coupled with subclonal LOH. As expected, based on the genomic profile, DL11 displayed membranous expression of all four IHC markers (both N- and C-terminal-E-cadherin, p120, and β-catenin, Supplementary Figs. 1–3). Nevertheless, none of the seven LLIMCas harbored the loss-of-function mutations characteristic of ILCs, and none of the *CDH1* alterations detected in the analyzed LLIMCas was biallelic. They are predicted not to affect gene function and are neither pathogenic nor clinically meaningful.

Eleven of the 14 cases (78%) subjected to MSK-IMPACT had sufficient SNVs mutational signatures (Fig. 5, Supplementary Table 3) to be decomposed by SigMA, a machine learning-based algorithm optimized for mutational signature decomposition of formalin-fixed paraffin-embedded samples analyzed with targeted capture sequencing data. This analysis revealed that 75% (3/4) of ILCs displayed a dominant APOBEC mutational signature compared to 57% (4/7) of LLIMCas (*p* > 0.05, Fisher’s exact test). Homologous recombination deficiency-related signature (HRD) was found to be dominant in 25% (1/4) of ILCs, and 43% (3/7) of LLIMCas displayed mutational signatures attributed to aging.

Seven LLIMCas were subjected to *CDH1* promoter methylation assessment by digital droplet PCR (ddPCR). This analysis revealed that four of the seven LLIMCas displayed *CDH1* promoter methylation (Fig. 5, supplementary table 3), and these cases displayed slightly reduced E-cadherin expression (intensity of E-cadherin staining classified by one author (R.B.) prior to methylation testing), including the 2 LLIMCas that harbored *CDH1* alterations (DL09 and DL11). Two of the remaining 3 LLIMCas were unmethylated, and in one case, the *CDH1* methylation status was not accessible as the results did not meet the qualitative standards. The comparative analysis of the repertoire of somatic genetic alterations, patterns of gene copy number profiles, and mutational signatures present in LLIMCas and classical ILCs did not reveal any significant differences.

## Discussion

Here we report a series of distinct breast carcinomas that we term as LLIMCa, characterized by low- to intermediate-grade uniform cells and exhibiting discohesive lobular-like growth patterns while maintaining intact adhesion molecule E-cadherin expression. In short, LLIMCa demonstrates some features similar to ILC but different from IDC, such as significantly larger pathologic tumor size than the size estimated on imaging, more frequent positive or close margins with breast-conserving surgery, higher Nottingham grade and score, and higher ME2 scores; some features similar to IDC such as higher rate of lymphovascular space invasion; and some features intermediate between ILC and IDC such as tumor size and pT stage. The study identifies several important findings worthy of close attention.

First, LLIMCas display a discohesive lobular-like morphologic pattern in the presence of membranous staining by E-cadherin (with both C- and N-terminal antibodies), p120, and β-catenin. A dysfunctional E-cadherin/catenin complex due to genetic or epigenetic biallelic inactivation of *CDH1* is the defining hallmark of ILC. The dynamic alteration of the E-cadherin/p120 complex is thought to be responsible for the changes in cell motility and, accordingly, the dispersed infiltrative morphology of ILC^6,7,11,21,29–33^. In the present study, the distinct membranous staining for all four currently used IHC markers of E-cadherin/catenin complex in LLIMCa suggests that the phenotypic appearance of discohesive, diffuse growth pattern observed in this particular type of invasive carcinoma has occurred in the presence of an intact E-cadherin/p120 junctional complex. Correspondingly, in the 14 randomly selected cases subjected to the exploratory genomic analyses, *CDH1* loss-of-function biallelic mutations are identified in all 7 bona fide ILC cases. In contrast, none of the 7 LLIMCa cases harbors the pathogenic *CDH1* loss-of-function mutation. Rather, the only CDH1 alterations detected in two of the LLIMCas consists of in-frame indels that are predicted not to affect gene function and are neither pathogenic nor clinically meaningful. It appears that the mechanisms driving the typical appearance of LLIMCa are not dependent on *CDH1* biallelic loss-of-function mutations or E-cadherin/catenin complex genomic alterations. However, *CDH1* promoter methylation assessment reveals that four of the seven LLIMCas are methylated. This result raises the tantalizing hypothesis that the abnormal (slightly reduced) E-cadherin expression pattern observed in these four cases, which is not observed in the unmethylated LLIMCas (strong membranous expression), might be associated with epigenetic mechanisms. Hence, the assessment of *CDH1* methylation status, using a sensitive ddPCR method and tissue microdissection to ensure high tumor purity, should be considered in the presence of even slightly reduced E-cadherin expression in cancers lacking bona fide biallelic *CDH1* pathogenic mutations. Our findings warrant further whole-genome sequencing, epigenomic and RNA-sequencing analyses to define the mechanistic basis for the phenotypic features of LLIMCas. Another finding worth noting is the presence of a dominant ABOBEC mutational signature in 57% (4 of 7 tested) of LLIMCas. ABOBEC signatures are more frequent in ILCs compared to IDCs and have been suggested as a biomarker of resistance to endocrine therapy^34^. This should also be further investigated.

Second, LLIMCas often harbor significantly larger pathologic tumor size than the size estimated on imaging and frequently positive or close margins at the first surgical procedure. This appears to be associated with lobular-like tumor morphology despite the preservation of the E-cadherin/catenin complex. In the past decade, multiple studies and consensus guidelines have recommended preoperative MRI for optimal management of patients with ILC, given its high sensitivity and better accuracy of measuring tumor size and multifocal disease^35–39^. Similar findings have been reported in “IDC with lobular features” on core biopsy^40^. Moreover, the percentage of aberrant E-cadherin and p120 expression was found to be irrelevant to additional diseases detected on MRI in such patients^41^. Given the findings in our study, we contend that distinguishing LLIMCas from the usual type IDCs in the biopsy report may be required and that preoperative MRI should be considered to guide surgical management for this type of invasive breast carcinoma.

Third, all three types of invasive carcinomas in our study cohort show essentially no statistically significant difference in hormone receptor status or prognosis, including long-term survival. Most of the previous studies included tumors of all nuclear grades^23,24^, whereas ILCs consist of significantly more low- to intermediate-grade Luminal A-type tumors and are more often hormone receptor-positive compared to IDCs. As a result, the differences reported in prior studies might have been differences between low- to intermediate-grade versus high-grade tumors rather than between ductal and lobular cancers. In our study design, we excluded all nuclear grade 3 tumors. Therefore, since we have a distinctively uniform low- to intermediate-grade tumor population with different phenotypes, the similar hormone receptor status, and survival outcome might be a reflection of the inherent nature of low-intermediate grade, ER-positive Luminal A-like tumors regardless of histologic phenotypes. Genomic studies of the mixed ductal and lobular carcinomas revealed that all components in such cases were frequently clonally related, suggesting shared origins of a common neoplastic clone^42^. Moreover, several lines of studies revealed that ILC shared common genetic alterations with low-grade IDC and direct clonal divergence from the ductal to the lobular phenotype occurred late in tumor evolution, where the aberrant E-cadherin expression appeared to be a key distinguishing switch^30,43,44^. Hence, the multistep model of breast cancer progression suggests that ILCs or tumors exhibiting lobular-like growth patterns may arise from the low-grade, ER-positive “ductal” pathway^10,42–46^. Whether LLIMCa represents an intermediate form in the phenotypic switch from ductal to lobular type is yet to be determined.

Lastly, although an accurate diagnosis of LLIMCa is required for proper pre-surgical evaluation and local therapy, the prognosis of these tumors remains defined by the traditional prognostic factors as well as the multivariable model ME2. Our survival analysis fails to show statistically significant differences when LLIMCa is compared to ILC or IDC, either by itself or in combined groups with IDC or ILC. In contrast, the traditional prognostic markers are associated with significantly improved survival. Interestingly, the ME2 score <18 is associated with significant improvement in all four survival measurements (RFS, DRFS, OS, and BCSS). Since ME2 only uses readily available histopathologic and IHC data from pathology reports at no additional cost, its use should be encouraged in routine practice.

Our study does have some limitations. The study cohort is retrospectively extracted from a single institution, a 6-year period archive, during which time we started routine E-cadherin/p120 stains on invasive tumors with lobular-like growth patterns. The retrospective nature and relatively small sample size could be the potential basis for the non-significant clinicopathologic and survival observations. In addition, only a limited number of cases are sequenced for the pilot molecular analyses. Ideally, multicenter prospective outcome studies along with whole-genome sequencing, epigenomic and RNA-sequencing analyses to define the mechanistic basis for the phenotypic features of LLIMCas would be useful to corroborate the findings.

Despite these limitations, we present a series of LLIMCas with morphologic features mimicking those of ILCs, but maintaining immunophenotypic membranous E-cadherin expression characteristic of IDCs, and typically spared of the pathogenic *CDH1* loss-of-function gene mutations. Their morphologic discohesion could be partly explained by *CDH1* promoter methylation in some but not all cases. One could potentially argue to classify all tumors with lobular-like growth patterns as ILCs regardless of E-cadherin expression in the current practice setting of breast cancer treatment. Yet, the interobserver agreement for the diagnosis of ILC without IHC is moderate at best. The holy grail of understanding different types of breast carcinoma is to develop more targeted and personalized clinical treatment; germane to this endeavor is the correct cataloging of distinct phenotypes in a systematic manner. We would contend that to facilitate future investigations in elucidating ductal versus lobular phenotypes at molecular, evolutional, functional, and therapeutic levels, accurately identifying LLIMCas and separating LLIMCas from the typical IDCs or ILCs would be justified. From a patient management standpoint, we suggest that preoperative MRI be entertained to guide the surgical management of patients with LLIMCa.

## Materials and methods

### Defining LLIMCa

Lobular-like IDCs (LLIMCas) in the current study were defined as invasive mammary carcinomas consisting of uniform discohesive cells with low to intermediate grade nuclei, dispersed growth pattern, and rare to absent tubule formation while maintaining moderate to strong uniform circumferential membranous reactivity for E-cadherin and p120 throughout the tumor.

### Case selection and IHC studies

The study protocol was approved by the University of Pittsburgh Institutional Review Board with a waiver of the informed consent. All invasive mammary carcinomas with ambiguous histomorphology for ductal or lobular differentiation and, therefore, with IHC C-terminus E-cadherin stain (Clone: 36; Catalog # 790-4497; Vendor: Ventana, Tucson, AZ; Dilution: ready to use [RTU]; Pre-treatment: CC1-S 64 min; Detection: Ultraview; Staining platform: Ventana Benchmark Ultra) performed were extracted from a 6-year (2004–2009) archive at UPMC Magee-Womens Hospital. Among them, the cases from 2007 to 2009 also had concurrent p120 stain (Clone: 98; Catalog # 610134; Vendor: BD Biosciences, Franklin Lakes, NJ; Dilution: 1:200; Pre-treatment: CC1-S; Detection: Ultraview; Staining platform: Ventana Benchmark Ultra) performed. Cases were classified as LLIMCas only if they displayed the criteria mentioned previously. In contrast, cases were classified as ILCs if they displayed absent and/or aberrant E-cadherin staining along with cytoplasmic p120 staining. Fourteen cases submitted for sequencing were also stained for beta-catenin (Clone: B-catenin-1; Catalog # M3539, Vendor: Dako, Santa Clara, CA; Dilution: 1:250; Pre-treatment: CC1 24 minutes; Detection: Optiview; Staining platform: Ventana Benchmark Ultra) and N-terminus E-cadherin antibody (Clone: 36B5; Catalog # PA0387, Vendor: Leica Biosystems, Deer Park, IL; Dilution: RTU; Pre-treatment: ER2 20 min; Detection: DAB polymer refine; Staining platform: Leica Bond III).

The following cases were excluded from the extracted cohort to allow for more homogeneous comparison among different tumor groups and more accurate assessment of clinical outcomes: (1) mixed ductal and lobular carcinomas with either mixed components or indeterminate variable E-cadherin and p120 staining patterns; (2) high grade (nuclear grade 3) carcinomas; (3) tubulolobular carcinomas; (3) microinvasive carcinomas (≤1 mm); (4) concurrent multifocal tumor with different morphology; (5) patients with neoadjuvant treatment; (6) patients with previous cancer diagnosis; (7) cases with no in-house primary surgical resection or incomplete follow-up information.

Ultimately, 166 cases of LLIMCa and 104 cases of ILC were identified. Additionally, 100 cases of grade 1 or 2 typical IDC (carcinoma, no special type) were retrieved over the same period for comparison analysis.

In addition, β-catenin stain (Clone: β-catenin-1; Vendor: Agilent (Dako), Santa Clara, CA; Dilution: 1:250; Pre-treatment: CC1 24’; Detection: OptiView; Staining platform: Ventana Benchmark Ultra) and N-terminal-E-cadherin stain (Clone: 36B5; Vendor: Leica, Deer Park, IL; Dilution: RTU; Pre-treatment: ER2, 20’; Detection: DAB Polymer Refine; Staining platform: Leica Bond III) were performed on the 14 LLIMCa and ILC cases randomly selected for the exploratory genomic tests.

### Tissue preparation and DNA extraction

Ten 8 μm-thick sections from each representative formalin-fixed paraffin-embedded (FFPE) tumor and matched normal tissue blocks of 7 LLIMCas and 7 classical ILCs were stained with nuclear fast red and subjected to microdissection using a sterile needle under a stereomicroscope (Olympus SZ61) to enrich tumor cell content, as previously described^47,48^. Genomic DNA was extracted from the tumor and matched normal tissue using the QIAamp DNA FFPE Tissue Kit (Qiagen) according to manufacturers’ instructions.

### MSK-IMPACT sequencing

Tumor and normal DNA from each case were subjected to massively parallel sequencing targeting all coding regions of 505 cancer-related genes using the FDA-approved MSK-IMPACT assay as previously described^47,49^. The median depth of coverage of tumor and normal samples was 758× (range: 502×–1167×) and 389× (range: 145×–540×), respectively. In brief, reads were aligned to the reference human genome GRCh37 using the Burrows–Wheeler Aligner (BWA v0.7.15)^50^. The Genome Analysis Toolkit (GATK. V3.1.1)^51^ was employed for local realignment, duplicate removal, and base quality recalibration. Somatic single nucleotide variants (SNVs) were detected by MuTect (v1.0)^52^, insertions, and deletions (indels) by Strelka^53^, Varscan2^54^, Scalpel^55^, and Lancet^56^. All mutations were manually inspected using the Integrative Genomics Viewer (IGV). The cancer cell fraction (CCF) of each mutation was inferred, as well as clonal probability, using ABSOLUTE^57^. Copy number alterations (CNAs) and LOH were determined using FACETS^58^. Mutations targeting hotspot loci were assigned according to Chang et al.^59^. Mutational signatures were inferred using Signature Multivariate Analysis (SigMA) based on all synonymous and nonsynonymous somatic mutations^60^. Exposure-based dominant mutational signatures obtained by SigMA, an algorithm previously validated for the analysis of formalin-fixed paraffin-embedded samples, were reported in cases with at least 5 SNVs (Supplementary Table 3), as previously described^48^. The repertoire of non-synonymous somatic mutations, mutational frequencies, and CNAs of classical ILCs were compared to genetic alterations affecting LLIMCas.

### *CDH1* promoter methylation assessment by digital droplet PCR

Following PicoGreen quantification, 0.2–9 ng bisulfite-treated genomic DNA was combined with locus-specific primers targeting the two *CDH1* promoter CpG islands, FAM- and HEX-labeled probes (Supplementary Table 3), the restriction enzyme HaeIII, and digital PCR Supermix for probes (no dUTP). CpG Methylated DNA (ThermoFisher, Waltham, MA) and Universal Unmethylated DNA (Millipore, Burlington, MA) were used as positive and negative controls, respectively. All reactions were performed on a QX200 ddPCR system (Bio-Rad, Hercules, CA), and each sample was evaluated in two technical duplicates.

Reactions were partitioned into ~41 K droplets per well using the QX200 droplet generator. Emulsified PCRs were run on a 96-well thermal cycler using the following cycling conditions: 95 °C 10’; 50 cycles of 94 °C 60’ and 54 °C 2’; 98 °C 10’. Plates were read and analyzed using the QuantaSoft software (Bio-Rad, Hercules, CA) to assess the number of droplets positive for *CDH1* promoter methylated, unmethylated, both, or neither. Methylation Frequency (MF) was inferred as MF = 100 * Methylated/(Methylated + Unmethylated). Methylation of the *CDH1* promoter was defined as higher than 35 methylated droplets.

### Clinical characteristics

A review of patient’s electronic medical records was conducted to obtain the following information: (1) age at diagnosis; (2) date of first diagnosis; (3) type of first surgical procedure; (4) adjuvant therapies; (5) date of first recurrence; (6) type of recurrence and site of distant metastasis; (7) date and status at last follow-up (for patients who developed non-breast new malignancies, the last contact date was censored at the date of new malignancy diagnosis); (8) date and cause of death.

### Pathologic characteristics

Pathology reports of the first surgical procedure were reviewed to obtain the following tumor pathologic characteristics: (1) multifocality and, if present, number of foci; (2) tumor size; (3) tumor Nottingham grade; (4) Nottingham score; (5) tumor-associated microcalcifications; (6) lymphovascular space invasion; (7) margin status at first surgery; (8) lymph node status; (9) pathologic stages of tumor (pT) and lymph node (pN).

### Predictive and prognostic biomarkers

Results of the IHC stains for ER, PR, HER2, and Ki67 were extracted from the pathology reports. ER and PR were scored by the modified histologic score (H-score) method, calculated by multiplying the intensity of expression (0–3) by the percentage of cells showing that intensity (0–100%). The sum of these numbers was referred to as the H-score. *H*-score ≥ 1 was considered positive. HER2 was scored per the FDA-cleared interpretation guide for Ventana anti-HER2 antibody. We started performing Ki67 stain on invasive breast carcinomas in late 2007, and the proliferation index was scored as a percentage of cells with staining of any intensity.

ME2 scores were calculated for all cases. ME2 is one of the multivariable models developed to estimate the Oncotype DX^®^ recurrence score^61–63^. ME1 and ME3 require a Ki-67 proliferation index, while ME2 does not. Since the Ki-67 proliferation index was unavailable for most of the cases, only ME2 scores were calculated.

### Tumor size pathology-radiology correlation

The final tumor size was obtained by pathologic evaluation of the primary surgical resection. The size of the tumor estimated radiologically was recorded from the mammographic or ultrasound imaging reports at the time of the diagnostic core biopsy. Pathology-to-radiology tumor size ratio was calculated and used as one of the variables for comparing different tumor types.

### Outcome and survival analysis

Clinical outcome analysis included recurrence rate, recurrence type, and sites of metastasis. Loco-regional recurrence was defined as a tumor arising in the treated breast/chest wall or within regional lymph nodes. Taking into account the findings published in the literature^64–66^, we separated the patterns of distant metastatic sites into lobular-like (gynecologic or gastrointestinal organs, regardless of any other site involvement), ductal-like (lung, pleura, liver, brain, lymph node, with or without bone involvement), and neutral (bone only).

Long-term survival data included RFS, DRFS, OS, and BCSS. RFS was defined as the time from diagnosis to first recurrence (local or distant) or the date of the last contact. DRFS was defined as the time from diagnosis to first distant recurrence or the date of the last contact. OS was defined as the time from diagnosis to death due to any cause or the date of the last contact. BCSS was defined as the time from diagnosis to death due to breast cancer or the date of the last contact.

### Statistical analysis

For comparison of means, independent sample *t*-tests were performed. Univariable analysis was performed using *χ*2 and Fisher exact tests to compare the differences in percentages between groups. A *p*-value < 0.05 was considered significant. Long-term survival data, including RFS, DRFS, OS, and BCSS, were analyzed via Kaplan–Meier curves for different tumor types (all 3 subtypes simultaneously, LLIMCa versus ILC, LLIMCa versus IDC, ILC versus IDC) and after combining LLIMCa with IDC (i.e., ILC versus IDC + LLIMCa) or ILC (i.e., IDC versus ILC + LLIMCa). A log-rank test was used to compare Kaplan–Meier curves. Survival analysis was also performed for known prognostic variables (grade, nodal status, pT stage, pN stage) and ME2 score categories. The variables showing statistically significant differences in survival by log-rank test were included for multivariable Cox proportional hazard regression analysis. Statistical analysis was performed using IBM SPSS Statistics for Windows, Armonk, NY: IBM Corp.

### Reporting summary

Further information on research design is available in the Nature Research Reporting Summary linked to this article.

## Supplementary information

Supplementary Information

Reporting Summary

Data Set 1

## Data Availability

The H&E and IHC datasets generated and analyzed during the current study are not publicly available but can be made available upon reasonable request, following ethics committee approval and a data transfer agreement, to guarantee the General Data Protection Regulation. All genomic data generated and analyzed during this study are available in the Sequencing Read Archive (SRA): SRP446067.
