# Supplementary material for: Clinicopathologic and genomic features of lobular like invasive mammary carcinoma: is it a distinct entity?
Source: NPJ Breast Cancer. 2023 Jul 13;9:60. doi: 10.1038/s41523-023-00566-7 (PMC10345141; doi:10.1038/s41523-023-00566-7)
Supplement: Supplementary file 1 — Supplementary Information [file 41523_2023_566_MOESM1_ESM.pdf]

Supplementary figure 1: ILC cases submitted for sequencing

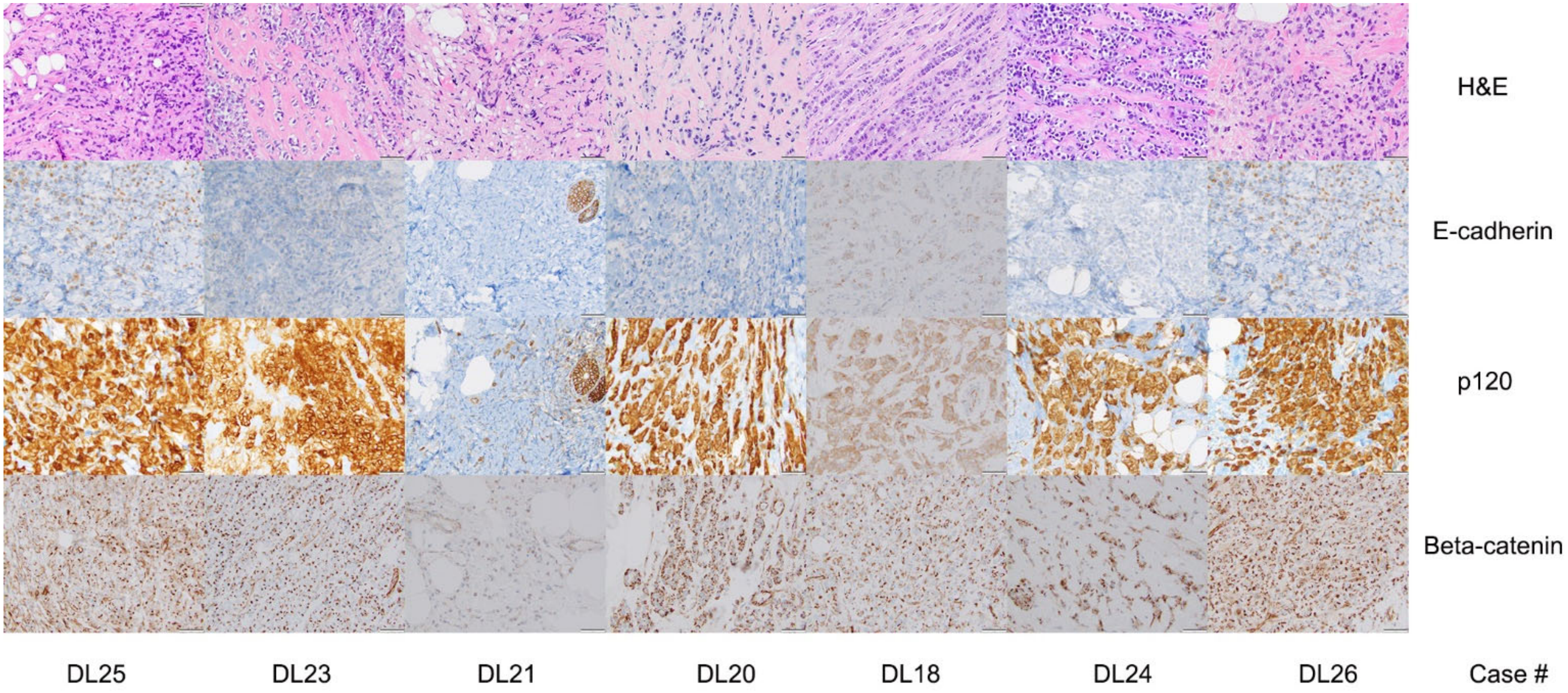

Supplementary figure 1: H&E stain shows single cell infiltrative growth pattern of classical invasive lobular carcinoma (ILC). E-cadherin stain shows lack of reactivity, p120 stain shows cytoplasmic reactivity, and beta-catenin shows aberrant granular cytoplasmic reactivity. Scale bar = 50 µm.

Supplementary figure 2: LLIMCa cases submitted for sequencing

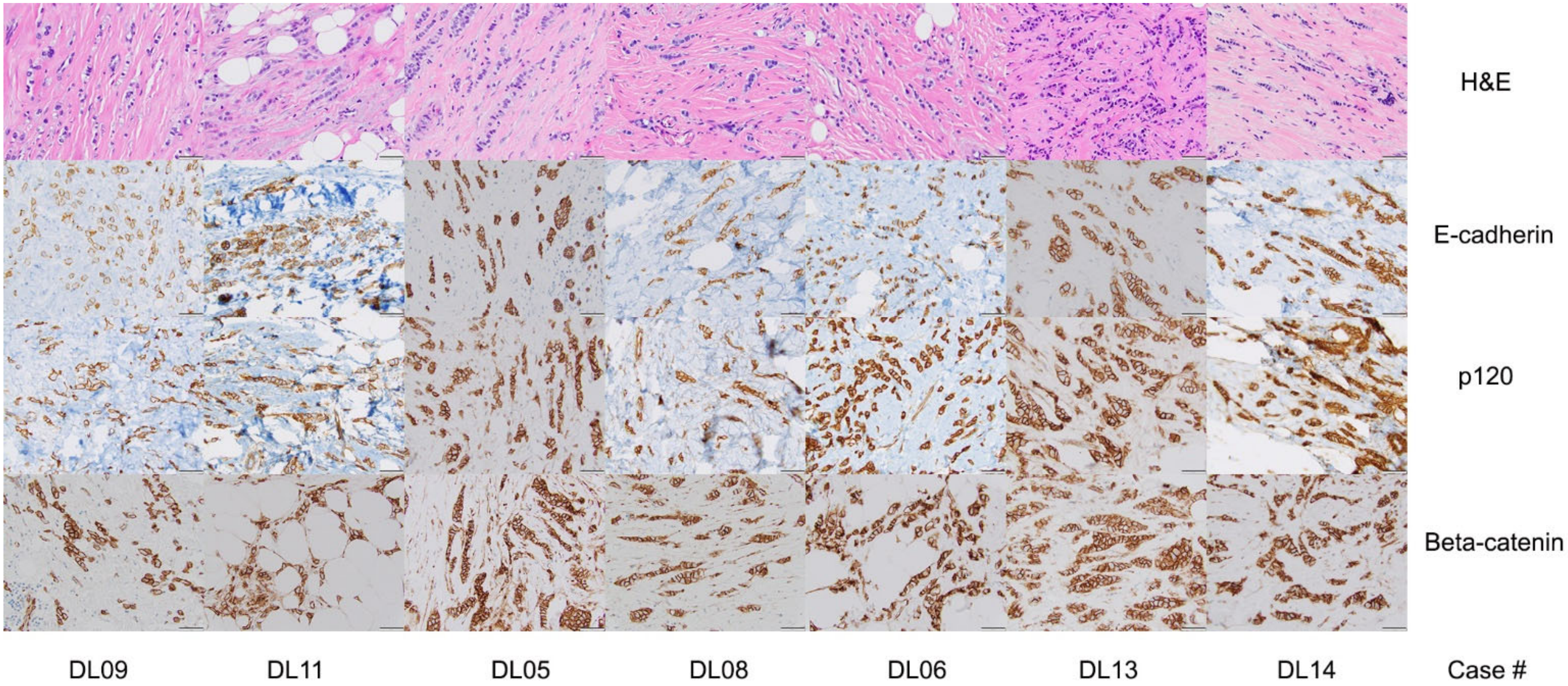

Supplementary figure 2: H&E stain shows single cell infiltrative growth pattern (lobular-like). However, all 3 stains- E-cadherin, p120, beta-catenin demonstrate circumferential membranous staining. LLIMCa: Lobular-like invasive mammary carcinoma. Scale bar = 50 µm.

Supplementary figure 3: E-cadherin N-terminus antibody staining

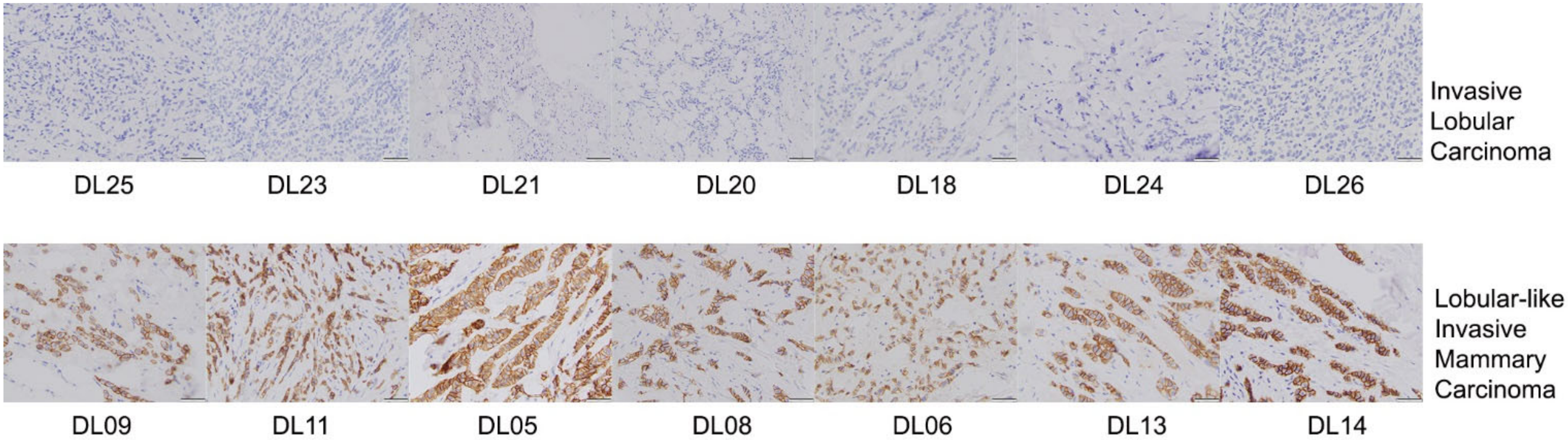

Supplementary figure 3: E-cadherin N-terminus antibody (clone 36B5, Leica) shows lack of reactivity in invasive lobular carcinomas that showed biallelic inactivation of *CDH1* on targeted sequencing (mutation coupled with loss of heterozygosity). However, the staining in lobular-like invasive mammary carcinomas is membranous, a pattern similar to what was observed with antibody clone 36 (directed against the cytoplasmic domain closer to C-terminus). Scale bar = 50 µm.

Supplementary figure 4: LLIMCa case (DL09) with subclonal *CDH1* mutation

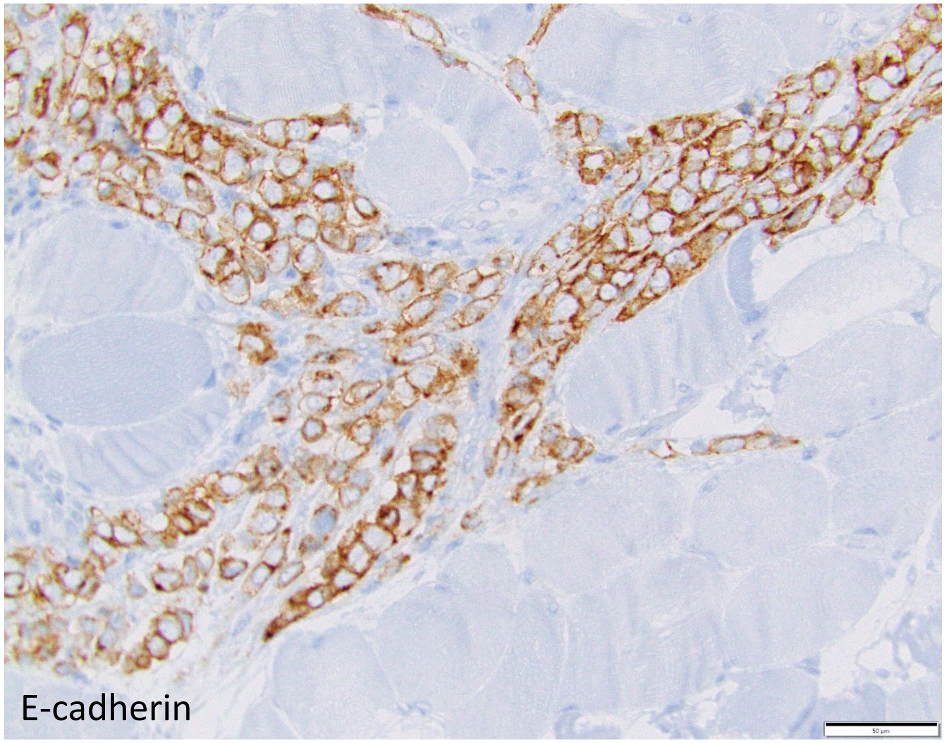

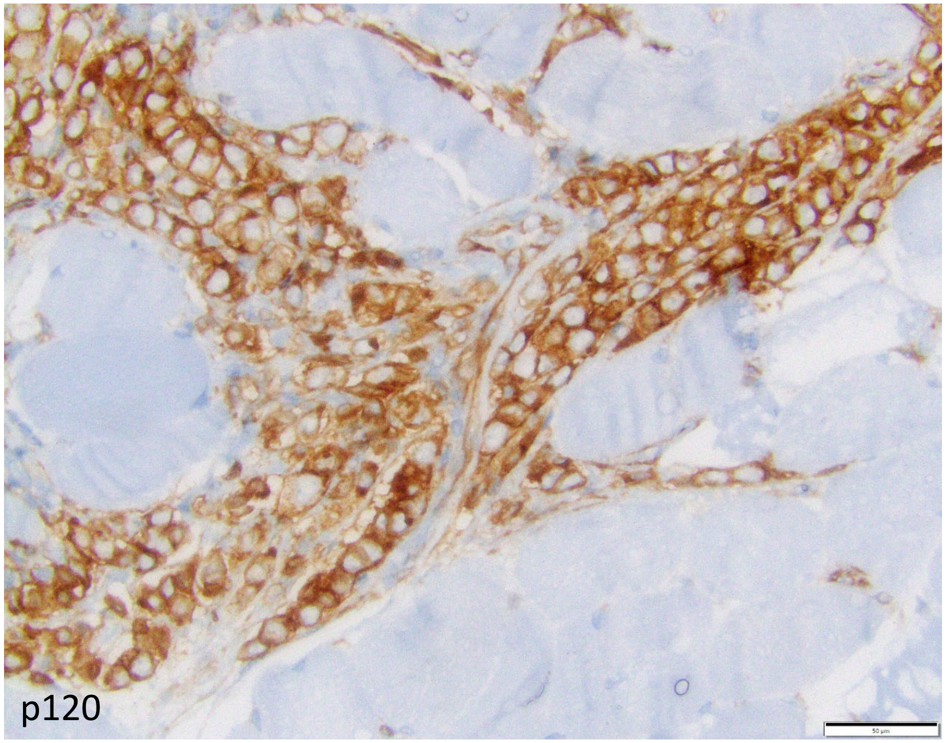

Supplementary figure 4: This case predominantly showed membranous E-cadherin and p120 reactivity (see supplementary figure 2), but focal areas showed aberrant (cytoplasmic) E-cadherin along with cytoplasmic p120. These areas were identified only after careful re-review. Scale bar = 50 µm.

Supplementary Table 1: List of non-synonymous somatic mutations identified in lobular and lobular-like invasive mammary carcinomas by targeted massively parallel sequencing (MSK-IMPACT).

Sample_ID	Chromosome	Position	REF	ALT	Gene Symbol	Variant Classification	AA change	Tumor MAF	Normal MAF	Tumor Depth	Normal Depth	Loss of Heterozygosity	Pathogenicity	Hotspot mutation	Cancer Gene Census	Kandoth et al	Lawrence et al	Cancer Cell Fraction	Purity	Clonal Status
DL05	20	31395677	G	A	DNMT3B	Missense Mutation	p.A844T	0.2573	0.0000	750	354	FALSE	Likely Pathogenic	FALSE	FALSE	FALSE	FALSE	1	0.35	Clonal
DL06	11	94911908	C	T	SESN3	Missense Mutation	p.R341Q	0.0426	0.0000	258	205	TRUE	Passenger	FALSE	FALSE	FALSE	FALSE	0.14	0.89	Subclonal
DL06	16	3830853	G	A	CREBBP	Missense Mutation	p.S568F	0.0394	0.0000	355	193	FALSE	Passenger	FALSE	TRUE	FALSE	TRUE	0.17	0.89	Subclonal
DL06	16	86544366	C	T	FOXF1	Missense Mutation	p.S64L	0.0625	0.0000	224	104	TRUE	Likely Pathogenic	FALSE	FALSE	FALSE	FALSE	0.2	0.89	Subclonal
DL06	17	7577156	C	A	TP53	Splice Site	p.X261_splice	0.4311	0.0000	225	157	TRUE	Likely Pathogenic	TRUE	TRUE	TRUE	TRUE	0.97	0.89	Clonal
DL08	10	112764488	G	T	SHOC2	Missense Mutation	p.R366L	0.2008	0.0000	508	237	FALSE	Passenger	FALSE	FALSE	FALSE	FALSE	0.7	0.58	Subclonal
DL08	12	115118706	T	A	TBX3	Missense Mutation	p.N212I	0.1810	0.0000	735	248	FALSE	Likely Pathogenic	FALSE	TRUE	TRUE	TRUE	0.63	0.58	Subclonal
DL08	2	29449939	C	G	ALK	Splice Region	p.V972V	0.1868	0.0000	589	310	FALSE	Passenger	FALSE	TRUE	FALSE	TRUE	0.65	0.58	Subclonal
DL08	3	178936082	G	A	PIK3CA	Missense Mutation	p.E542K	0.1752	0.0000	645	275	FALSE	Likely Pathogenic	TRUE	TRUE	TRUE	TRUE	0.61	0.58	Subclonal
DL09	1	72748143	C	T	NEGR1	Missense Mutation	p.C12Y	0.0291	0.0000	687	396	FALSE	Passenger	FALSE	FALSE	FALSE	FALSE	0.23	0.25	Subclonal
DL09	1	120462998	G	A	NOTCH2	Missense Mutation	p.S1778L	0.0740	0.0000	473	327	FALSE	Likely Pathogenic	FALSE	TRUE	FALSE	FALSE	0.58	0.25	Subclonal
DL09	11	102195694	C	G	BIRC3	Missense Mutation	p.Q152E	0.1468	0.0000	586	437	FALSE	Passenger	FALSE	TRUE	FALSE	FALSE	1	0.25	Clonal
DL09	13	32907081	C	T	BRCA2	Missense Mutation	p.S489F	0.1107	0.0000	533	360	FALSE	Passenger	FALSE	TRUE	TRUE	FALSE	0.87	0.25	Clonal
DL09	13	103525616	C	T	ERCC5	Nonsense Mutation	p.Q963*	0.0667	0.0000	405	337	FALSE	Likely Pathogenic	FALSE	TRUE	FALSE	FALSE	0.52	0.25	Subclonal
DL09	14	38061459	G	A	FOXA1	Missense Mutation	p.S177L	0.0336	0.0031	595	324	FALSE	Likely Pathogenic	FALSE	TRUE	TRUE	TRUE	0.26	0.25	Subclonal
DL09	15	51503112	C	G	CYP19A1	Missense Mutation	p.E469Q	0.1339	0.0000	381	281	FALSE	Passenger	FALSE	FALSE	FALSE	FALSE	1	0.25	Clonal
DL09	16	2106696	G	A	TSC2	Missense Mutation	p.E234K	0.0828	0.0033	664	307	FALSE	Passenger	FALSE	TRUE	FALSE	FALSE	0.73	0.25	Subclonal
DL09	16	68849510	CACCGTCACCGTG	C	CDH1	In-Frame Deletion	p.T472_V475del	0.0588	0.0000	340	322	TRUE	Potentially Pathogenic	FALSE	TRUE	TRUE	TRUE	0.4	0.25	Subclonal
DL09	19	50909524	G	A	POLD1	Missense Mutation	p.R443Q	0.0892	0.0020	740	510	FALSE	Passenger	FALSE	FALSE	FALSE	FALSE	0.88	0.25	Clonal
DL09	2	25463195	C	G	DNMT3A	Missense Mutation	p.K766N	0.0360	0.0000	584	375	FALSE	Likely Pathogenic	FALSE	TRUE	TRUE	TRUE	0.28	0.25	Subclonal
DL09	2	61148922	C	T	REL	Missense Mutation	p.A371V	0.1588	0.0000	359	310	FALSE	Passenger	FALSE	TRUE	FALSE	FALSE	1	0.25	Clonal
DL09	20	41419853	G	T	PTPRT	Missense Mutation	p.F156L	0.1061	0.0000	622	368	FALSE	Passenger	FALSE	TRUE	FALSE	FALSE	0.83	0.25	Clonal
DL09	3	71026154	C	T	FOXP1	Missense Mutation	p.E490K	0.0976	0.0000	420	332	FALSE	Likely Pathogenic	FALSE	TRUE	FALSE	FALSE	0.77	0.25	Subclonal
DL09	3	178916939	AAGAAAAGAT	A	PIK3CA	In-Frame Deletion	p.E109_I112delinsD	0.0775	0.0000	426	421	FALSE	Potentially Pathogenic	FALSE	TRUE	TRUE	TRUE	0.61	0.25	Subclonal
DL09	5	56160643	GCCGTGTTA	G	MAP3K1	Frame-Shift Deletion	p.R307Qfs*16	0.1264	0.0023	633	426	FALSE	Likely Pathogenic	FALSE	FALSE	TRUE	TRUE	0.99	0.25	Clonal
DL09	6	32163720	C	A	NOTCH4	Nonsense Mutation	p.E1836*	0.1210	0.0000	529	209	FALSE	Passenger	FALSE	FALSE	FALSE	FALSE	0.95	0.25	Clonal
DL09	7	152345809	G	A	XRCC2	Missense Mutation	p.S254L	0.0319	0.0054	408	368	FALSE	Passenger	FALSE	FALSE	FALSE	FALSE	0.25	0.25	Subclonal
DL09	8	145740776	C	G	RECQL4	Missense Mutation	p.E442Q	0.0337	0.0000	594	396	FALSE	Passenger	FALSE	TRUE	FALSE	FALSE	0.26	0.25	Subclonal
DL09	X	15833840	C	T	ZRSR2	Missense Mutation	p.L200F	0.0495	0.0000	384	315	FALSE	Likely Pathogenic	FALSE	TRUE	FALSE	FALSE	0.39	0.25	Subclonal
DL11	16	68849403	GT	G	CDH1	Intron	.	0.0954	0.0000	367	374	FALSE	.	FALSE	TRUE	TRUE	TRUE	0.92	0.21	Clonal
DL11	16	68849411	TCTCTAGG	T	CDH1	Splice Site	p.X441_splice	0.1108	0.0000	370	394	FALSE	Likely Pathogenic	FALSE	TRUE	TRUE	TRUE	1	0.21	Clonal
DL11	16	68849424	GATTTTGA	G	CDH1	Frame-Shift Deletion	p.D443Gfs*10	0.1088	0.0000	386	403	FALSE	Likely Pathogenic	FALSE	TRUE	TRUE	TRUE	1	0.21	Clonal
DL11	2	39294771	C	T	SOS1	Missense Mutation	p.E71K	0.0607	0.0000	659	403	FALSE	Likely Pathogenic	FALSE	FALSE	FALSE	FALSE	0.71	0.21	Subclonal
DL11	3	49405948	C	T	RHOA	Missense Mutation	p.E64K	0.0650	0.0028	646	355	FALSE	Likely Pathogenic	FALSE	TRUE	FALSE	TRUE	0.76	0.21	Subclonal
DL11	3	178936082	G	A	PIK3CA	Missense Mutation	p.E542K	0.1989	0.0019	724	516	FALSE	Likely Pathogenic	TRUE	TRUE	TRUE	TRUE	1	0.21	Clonal
DL13	16	67063356	G	T	CBFB	Nonsense Mutation	p.E16*	0.3887	0.0000	319	209	TRUE	Likely Pathogenic	FALSE	TRUE	TRUE	TRUE	0.89	0.61	Clonal
DL13	17	7577517	A	C	TP53	Missense Mutation	p.I255S	0.4518	0.0000	425	289	TRUE	Likely Pathogenic	TRUE	TRUE	TRUE	TRUE	1	0.61	Clonal
DL13	2	61128161	A	C	REL	Missense Mutation	p.K113Q	0.0278	0.0000	684	347	FALSE	Passenger	FALSE	TRUE	FALSE	FALSE	0.09	0.61	Subclonal
DL14	2	198266518	A	C	SF3B1	Missense Mutation	p.L773R	0.2287	0.0000	726	127	FALSE	Likely Pathogenic	FALSE	TRUE	TRUE	TRUE	0.71	0.64	Subclonal
DL14	3	178952085	A	G	PIK3CA	Missense Mutation	p.H1047R	0.3193	0.0000	736	128	FALSE	Likely Pathogenic	TRUE	TRUE	TRUE	TRUE	1	0.64	Clonal
DL14	5	56161735	TTG	T	MAP3K1	Frame-Shift Deletion	p.V412Ffs*13	0.2717	0.0000	1104	164	FALSE	Likely Pathogenic	FALSE	FALSE	TRUE	TRUE	1	0.64	Clonal
DL18	12	115117373	TGTACTATAAGG	T	TBX3	Frame-Shift Deletion	p.P264Ifs*10	0.2352	0.0000	982	357	FALSE	Likely Pathogenic	FALSE	TRUE	TRUE	TRUE	0.74	0.63	Subclonal
DL18	16	67100582	T	G	CBFB	Splice Region		0.5018	0.0033	544	305	TRUE	Passenger	FALSE	TRUE	TRUE	TRUE	1	0.63	Clonal
DL18	16	68849654	GA	G	CDH1	Frame-Shift Deletion	p.I521*	0.4625	0.0035	534	283	TRUE	Likely Pathogenic	FALSE	TRUE	TRUE	TRUE	1	0.63	Clonal
DL18	3	178936091	G	A	PIK3CA	Missense Mutation	p.E545K	0.3503	0.0000	725	383	FALSE	Likely Pathogenic	TRUE	TRUE	TRUE	TRUE	1	0.63	Clonal
DL18	7	151860431	GTC	G	KMT2C	Frame-Shift Deletion	p.R3410Tfs*11	0.3005	0.0000	1035	431	FALSE	Likely Pathogenic	FALSE	TRUE	TRUE	TRUE	0.95	0.63	Clonal
DL20	1	36941278	G	A	CSF3R	Splice Region		0.0547	0.0037	457	267	FALSE	Passenger	FALSE	TRUE	FALSE	FALSE	0.29	0.47	Subclonal
DL20	16	68842666	CT	C	CDH1	Frame-Shift Deletion	p.V202Lfs*13	0.4865	0.0000	592	411	TRUE	Likely Pathogenic	FALSE	TRUE	TRUE	TRUE	1	0.47	Clonal
DL20	19	10982398	C	T	CARM1	Missense Mutation	p.A7V	0.1176	0.0000	34	47	FALSE	Passenger	FALSE	FALSE	FALSE	FALSE	0.62	0.47	Subclonal
DL21	16	68857525	GC	G	CDH1	Frame-Shift Deletion	p.L721*	0.1636	0.0084	269	119	TRUE	Likely Pathogenic	FALSE	TRUE	TRUE	TRUE	0.82	0.33	Clonal
DL23	12	115117309	C	G	TBX3	Splice Site	p.X288_splice	0.4158	0.0000	558	382	FALSE	Likely Pathogenic	FALSE	TRUE	TRUE	TRUE	1	0.74	Clonal
DL23	16	68842386	CAGAAGACA	C	CDH1	Frame-Shift Deletion	p.R150Efs*15	0.5697	0.0000	330	367	TRUE	Likely Pathogenic	FALSE	TRUE	TRUE	TRUE	0.96	0.74	Clonal
DL23	3	63973888	GCCCCTCCTAGAACGT	G	ATXN7	In-Frame Deletion	p.P418_S422del	0.0718	0.0000	682	715	FALSE	Passenger	FALSE	FALSE	FALSE	FALSE	0.19	0.74	Subclonal
DL23	5	79950724	G	C	MSH3	Missense Mutation	p.A60P	0.1206	0.0000	141	40	FALSE	Passenger	FALSE	FALSE	FALSE	FALSE	0.45	0.74	Subclonal
DL24	1	36359751	C	T	AGO1	Missense Mutation	p.R255C	0.0262	0.0000	763	211	TRUE	Passenger	FALSE	FALSE	FALSE	FALSE	0.09	0.61	Subclonal
DL24	15	99192893	C	A	IGF1R	Missense Mutation	p.T28K	0.3571	0.0000	616	151	FALSE	Passenger	FALSE	FALSE	FALSE	FALSE	1	0.61	Clonal
DL24	16	68842595	G	T	CDH1	Splice Site	p.X178_splice	0.5019	0.0000	526	240	TRUE	Likely Pathogenic	FALSE	TRUE	TRUE	TRUE	1	0.61	Clonal
DL24	7	55221710	C	T	EGFR	Missense Mutation	p.R252C	0.0344	0.0000	669	215	FALSE	Likely Pathogenic	TRUE	TRUE	TRUE	TRUE	0.11	0.61	Subclonal
DL25	1	46511684	G	A	PIK3R3	Nonsense Mutation	p.R365*	0.1654	0.0058	786	515	TRUE	Likely Pathogenic	FALSE	FALSE	FALSE	FALSE	0.94	0.43	Clonal
DL25	10	114799776	C	T	TCF7L2	Splice Region		0.1725	0.0032	510	316	FALSE	Passenger	FALSE	TRUE	FALSE	TRUE	1	0.43	Clonal
DL25	12	115111972	G	A	TBX3	Nonsense Mutation	p.Q590*	0.3443	0.0018	793	566	TRUE	Likely Pathogenic	FALSE	TRUE	TRUE	TRUE	1	0.43	Clonal
DL25	14	92537353	C	CGCTGCTGCTGCT	ATXN3	In-Frame Insertion	p.G306delinsEQQQR	0.3846	0.0000	559	250	FALSE	Passenger	FALSE	FALSE	FALSE	FALSE	1	0.43	Clonal
DL25	14	105237188	CAG	C	AKT1	Splice Region		0.1256	0.0000	661	287	FALSE	Passenger	FALSE	TRUE	TRUE	TRUE	0.84	0.43	Clonal
DL25	15	43705515	C	G	TP53BP1	Missense Mutation	p.E1703Q	0.1355	0.0000	716	397	FALSE	Passenger	FALSE	FALSE	FALSE	FALSE	0.91	0.43	Clonal
DL25	16	68845591	C	CTCTGTGA	CDH1	Frame-Shift Insertion	p.M282Ifs*13	0.1415	0.0000	615	367	TRUE	Likely Pathogenic	FALSE	TRUE	TRUE	TRUE	0.81	0.43	Clonal
DL25	17	7578179	C	A	TP53	Nonsense Mutation	p.E224*	0.1904	0.0020	751	505	TRUE	Likely Pathogenic	FALSE	TRUE	TRUE	TRUE	1	0.43	Clonal
DL25	18	48591825	G	C	SMAD4	Missense Mutation	p.E330Q	0.0505	0.0000	732	510	TRUE	Likely Pathogenic	TRUE	TRUE	TRUE	TRUE	0.29	0.43	Subclonal
DL25	2	128018929	G	GA	ERCC3	Splice Region		0.1028	0.0031	632	319	FALSE	Passenger	FALSE	TRUE	FALSE	FALSE	0.69	0.43	Subclonal
DL25	3	178936091	G	A	PIK3CA	Missense Mutation	p.E545K	0.1506	0.0000	757	510	TRUE	Likely Pathogenic	TRUE	TRUE	TRUE	TRUE	1	0.43	Clonal
DL25	3	181430398	G	A	SOX2	Missense Mutation	p.E84K	0.1841	0.0024	782	420	TRUE	Likely Pathogenic	FALSE	TRUE	FALSE	FALSE	1	0.43	Clonal
DL25	5	31449514	G	A	DROSHA	Missense Mutation	p.H899Y	0.1537	0.0000	683	328	FALSE	Likely Pathogenic	FALSE	TRUE	FALSE	FALSE	1	0.43	Clonal
DL25	9	8340472	G	A	PTPRD	Splice Region		0.1006	0.0000	616	332	TRUE	Passenger	FALSE	FALSE	FALSE	FALSE	0.67	0.43	Subclonal
DL26	12	133245305	C	T	POLE	Missense Mutation	p.E648K	0.1263	0.0052	665	388	FALSE	Passenger	FALSE	TRUE	FALSE	TRUE	0.82	0.37	Clonal
DL26	16	68849663	G	A	CDH1	Splice Site	p.X522_splice	0.1242	0.0032	330	317	TRUE	Likely Pathogenic	FALSE	TRUE	TRUE	TRUE	0.68	0.37	Subclonal
DL26	17	38792273	C	G	SMARCE1	Missense Mutation	p.A151P	0.0278	0.0000	540	347	FALSE	Passenger	FALSE	TRUE	FALSE	FALSE	0.18	0.37	Subclonal
DL26	2	46605104	G	A	EPAS1	Missense Mutation	p.E441K	0.0996	0.0000	532	414	FALSE	Passenger	FALSE	TRUE	FALSE	FALSE	0.64	0.37	Subclonal
DL26	2	61753572	G	A	XPO1	Nonsense Mutation	p.Q71*	0.1708	0.0000	439	336	FALSE	Likely Pathogenic	FALSE	TRUE	FALSE	TRUE	1	0.37	Clonal
DL26	3	178936082	G	A	PIK3CA	Missense Mutation	p.E542K	0.1385	0.0000	585	378	FALSE	Likely Pathogenic	TRUE	TRUE	TRUE	TRUE	1	0.37	Clonal
DL26	4	1980443	C	T	WHSC1	Missense Mutation	p.S1302L	0.2753	0.0000	454	356	FALSE	Passenger	FALSE	TRUE	FALSE	FALSE	1	0.37	Clonal
DL26	5	38945713	C	T	RICTOR	Missense Mutation	p.E1505K	0.0634	0.0000	599	351	FALSE	Passenger	FALSE	FALSE	FALSE	FALSE	0.47	0.37	Subclonal
DL26	7	41729289	C	T	INHBA	Missense Mutation	p.D414N	0.3374	0.0000	406	265	FALSE	Likely Pathogenic	FALSE	FALSE	FALSE	FALSE	1	0.37	Clonal
DL26	X	44922802	C	T	KDM6A	Nonsense Mutation	p.Q555*	0.0994	0.0000	513	312	FALSE	Likely Pathogenic	TRUE	TRUE	TRUE	TRUE	0.64	0.37	Subclonal
DL26	X	76953095	G	A	ATRX	Missense Mutation	p.S73F	0.0906	0.0000	331	241	FALSE	Passenger	FALSE	TRUE	TRUE	FALSE	0.59	0.37	Subclonal

Supplementary Table 2: List of signature signals identified by SigMA in lobular and lobular-like invasive mammary carcinomas by targeted massively parallel sequencing (MSK-IMPACT).

Sample	Histology	Mutation_count	DomSig_Exposure	DomSig_Sigma	Signature.Clock	ignature.APOBE	Signature.HRD	Signature.MSI	gnature.Smoki
DL05	LLIMCa	5	Signature.Clock	Signature_clock	0.74553582	0.25446418	0	0	0
DL06	LLIMCa	13	Signature.APOBEC	ignature_APOBE	0.136128316	0.772028627	0.091843056	0	0
DL08	LLIMCa	11	Signature.APOBEC	ignature_APOBE	0	0.60495295	0.39504705	0	0
DL09	LLIMCa	39	Signature.APOBEC	ignature_APOBE	0.239570599	0.760429401	0	0	0
DL11	LLIMCa	10	Signature.APOBEC	ignature_APOBE	0.271219158	0.66150755	0	0	0
DL13	LLIMCa	9	Signature.Clock	Signature_17	0.621796421	0	0.214952027	0	0
DL14	LLIMCa	5	Signature.Clock	Signature_17	0.527823767	0.017164336	0	0	0
DL18	ILC	NA	NA	NA	NA	NA	NA	NA	NA
DL20	ILC	NA	NA	NA	NA	NA	NA	NA	NA
DL21	ILC	NA	NA	NA	NA	NA	NA	NA	NA
DL23	ILC	5	Signature.HRD	Signature_3_lc	0.009179166	0.048659129	0.942161704	0	0
DL24	ILC	9	Signature.APOBEC	ignature_APOBE	0.316310018	0.503342109	0	0	0
DL25	ILC	37	Signature.APOBEC	ignature_APOBE	0.020271207	0.931036628	0	0	0
DL26	ILC	19	Signature.APOBEC	ignature_APOBE	0.052990907	0.825120867	0.002141983	0	0

Sample	Signature.1	Signature.2	Signature.3	Signature.4	Signature.5	Signature.6	Signature.7	Signature.8	Signature.9	Signature.10	Signature.11	Signature.12	Signature.13	Signature.14	Signature.15
DL05	0.151618148	0.25446418	0	0	0.5939177	0	0	0	0	0	0	0	0	0	0
DL06	0.136128316	0.772028627	0	0	0	0	0	0.0918431	0	0	0	0	0	0	0
DL08	0	0.484149908	0	0	0	0	0	0.3950471	0	0	0	0	0.12080304	0	0
DL09	0.031289417	0.323791157	0	0	0.2082812	0	0	0	0	0	0	0	0.43663824	0	0
DL11	0	0.66150755	0	0	0.2712192	0	0	0	0	0	0	0	0	0	0
DL13	0.044885556	0	0	0	0.5769109	0	0	0.214952	0	0	0	0	0	0	0
DL14	0	0.017164336	0	0	0.5278238	0	0	0	0	0	0	0	0	0	0
DL18	NA	NA	NA	NA	NA	NA	NA	NA	NA	NA	NA	NA	NA	NA	NA
DL20	NA	NA	NA	NA	NA	NA	NA	NA	NA	NA	NA	NA	NA	NA	NA
DL21	NA	NA	NA	NA	NA	NA	NA	NA	NA	NA	NA	NA	NA	NA	NA
DL23	0	0.048659129	0.9421617	0	0.0091792	0	0	0	0	0	0	0	0	0	0
DL24	0.316310018	0.503342109	0	0	0	0	0	0	0	0	0	0	0	0	0
DL25	0.020271207	0.526166738	0	0	0	0	0	0	0	0	0	0	0.40486989	0	0
DL26	0.052990907	0.825120867	0.002142	0	0	0	0	0	0	0	0	0	0	0	0

Sample	Signature.16	Signature.17	Signature.18	Signature.19	Signature.20	Signature.21	Signature.22	Signature.23	Signature.24	Signature.25	Signature.26	Signature.27	Signature.28	Signature.29	Signature.30
DL05	0	0	0	0	0	0	0	0	0	0	0	0	0	0	0
DL06	0	0	0	0	0	0	0	0	0	0	0	0	0	0	0
DL08	0	0	0	0	0	0	0	0	0	0	0	0	0	0	0
DL09	0	0	0	0	0	0	0	0	0	0	0	0	0	0	0
DL11	0	0.067273293	0	0	0	0	0	0	0	0	0	0	0	0	0
DL13	0	0	0.16325155	0	0	0	0	0	0	0	0	0	0	0	0
DL14	0	0.455011897	0	0	0	0	0	0	0	0	0	0	0	0	0
DL18	NA	NA	NA	NA	NA	NA	NA	NA	NA	NA	NA	NA	NA	NA	NA
DL20	NA	NA	NA	NA	NA	NA	NA	NA	NA	NA	NA	NA	NA	NA	NA
DL21	NA	NA	NA	NA	NA	NA	NA	NA	NA	NA	NA	NA	NA	NA	NA
DL23	0	0	0	0	0	0	0	0	0	0	0	0	0	0	0
DL24	0	0	0.18034787	0	0	0	0	0	0	0	0	0	0	0	0
DL25	0	0.000979729	0.04771244	0	0	0	0	0	0	0	0	0	0	0	0
DL26	0	0.008188697	0.11155755	0	0	0	0	0	0	0	0	0	0	0	0

Supplementary Table 3: List of FAM- and HEX-labeled probes used in the ddPCR.

Assay name	Forward sequence	Reverse sequence	Probe sequence	Fluorophore
CDH1_1_NonMethyl	AGTAATTTTAGGTTAGAGGGTT	AAATTCACCTACCAACCAC	TTGTGTTTATGTGAGGTTGG	HEX
CDH1_2_Methyl	TGCGGAAGTTAGTTTAGATT	AACCCATAACTAACCGAAAA	CGTTTTAGTTCGGTTCGATT	FAM
CDH1_2_NonMethyl	TTTGTGGAAGTTAGTTTAGATTT	CTAACCAAAAACACCAAACA	TGATTGTATTTGGTGTTTGTTT	HEX
CDH1_1_methyl	AGTAATTTTAGGTTAGAGGGTT	GACCACAACCAATCAACA	CGTTAGTTTCGTTTTGGGG	FAM
