# Supplementary material for: Clinicopathologic and genomic features of lobular like invasive mammary carcinoma: is it a distinct entity?
Source: NPJ Breast Cancer. 2023 Jul 13;9:60. doi: 10.1038/s41523-023-00566-7 (PMC10345141; doi:10.1038/s41523-023-00566-7)
Supplement: Supplementary file 2 — Reporting Summary [file 41523_2023_566_MOESM2_ESM.pdf]

nature portfolio

Corresponding author(s): Jing Yu, Jorge Reis-Filho, Rohit Bhargava

Last updated by author(s): Jun 29, 2023

Reporting Summary

Nature Portfolio wishes to improve the reproducibility of the work that we publish. This form provides structure for consistency and transparency in reporting. For further information on Nature Portfolio policies, see our Editorial Policies and the Editorial Policy Checklist.

Statistics

For all statistical analyses, confirm that the following items are present in the figure legend, table legend, main text, or Methods section.

n/a	Confirmed	
☐	☒	The exact sample size (*n*) for each experimental group/condition, given as a discrete number and unit of measurement
☐	☒	A statement on whether measurements were taken from distinct samples or whether the same sample was measured repeatedly
☐	☒	The statistical test(s) used AND whether they are one- or two-sided<br>*Only common tests should be described solely by name; describe more complex techniques in the Methods section.*
☐	☒	A description of all covariates tested
☒	☐	A description of any assumptions or corrections, such as tests of normality and adjustment for multiple comparisons
☒	☐	A full description of the statistical parameters including central tendency (e.g. means) or other basic estimates (e.g. regression coefficient) AND variation (e.g. standard deviation) or associated estimates of uncertainty (e.g. confidence intervals)
☒	☐	For null hypothesis testing, the test statistic (e.g. *F*, *t*, *r*) with confidence intervals, effect sizes, degrees of freedom and *P* value noted<br>*Give P values as exact values whenever suitable.*
☒	☐	For Bayesian analysis, information on the choice of priors and Markov chain Monte Carlo settings
☒	☐	For hierarchical and complex designs, identification of the appropriate level for tests and full reporting of outcomes
☒	☐	Estimates of effect sizes (e.g. Cohen's *d*, Pearson's *r*), indicating how they were calculated

Our web collection on statistics for biologists contains articles on many of the points above.

Software and code

Policy information about availability of computer code

Data collection	N/A
Data analysis	N/A

For manuscripts utilizing custom algorithms or software that are central to the research but not yet described in published literature, software must be made available to editors and reviewers. We strongly encourage code deposition in a community repository (e.g. GitHub). See the Nature Portfolio guidelines for submitting code & software for further information.

Data

Policy information about availability of data

All manuscripts must include a data availability statement. This statement should provide the following information, where applicable:

- Accession codes, unique identifiers, or web links for publicly available datasets
- A description of any restrictions on data availability
- For clinical datasets or third party data, please ensure that the statement adheres to our policy

The H&E and immunohistochemistry datasets generated and analyzed during the current study are not publicly available but can be made available upon reasonable request, following ethics committee approval and a data transfer agreement, to guarantee the General Data Protection Regulation. All genomic data generated and analyzed during this study are available in the Sequencing Read Archive (SRA): SRP446067.

Research involving human participants, their data, or biological material

Policy information about studies with human participants or human data. See also policy information about sex, gender (identity/presentation), and sexual orientation and race, ethnicity and racism.

Reporting on sex and gender	N/A
Reporting on race, ethnicity, or other socially relevant groupings	N/A
Population characteristics	N/A
Recruitment	No human subject involved. Study included a series of pathological breast cancer cases.
Ethics oversight	University of Pittsburgh Institutional Review Board

Note that full information on the approval of the study protocol must also be provided in the manuscript.

Field-specific reporting

Please select the one below that is the best fit for your research. If you are not sure, read the appropriate sections before making your selection.

☒ Life sciences ☐ Behavioural & social sciences ☐ Ecological, evolutionary & environmental sciences

For a reference copy of the document with all sections, see nature.com/documents/nr-reporting-summary-flat.pdf

Life sciences study design

All studies must disclose on these points even when the disclosure is negative.

Sample size	No sample size calculation performed. Included a series of special type pathologic breast cancer cases over a period of time.
Data exclusions	The following cases were excluded: (1) mixed ductal and lobular carcinomas with either mixed components or indeterminate variable E-cadherin and p120 staining patterns; (2) high grade (nuclear grade 3) carcinomas; (3) tubulolobular carcinomas; (4) microinvasive carcinomas (≤ 1 mm); (4) concurrent multifocal tumor with different morphology; (5) patients with neoadjuvant treatment; (6) patients with previous cancer diagnosis; (7) cases with no in-house primary surgical resection or incomplete follow-up information.
Replication	N/A
Randomization	N/A
Blinding	N/A

Reporting for specific materials, systems and methods

We require information from authors about some types of materials, experimental systems and methods used in many studies. Here, indicate whether each material, system or method listed is relevant to your study. If you are not sure if a list item applies to your research, read the appropriate section before selecting a response.

Materials & experimental systems

n/a	Involved in the study	
☐	☒	Antibodies
☒	☐	Eukaryotic cell lines
☒	☐	Palaeontology and archaeology
☒	☐	Animals and other organisms
☐	☒	Clinical data
☒	☐	Dual use research of concern
☒	☐	Plants

Methods

n/a	Involved in the study	
☒	☐	ChIP-seq
☒	☐	Flow cytometry
☒	☐	MRI-based neuroimaging

Antibodies

Antibodies used	The following antibodies were used: C-terminus E-cadherin stain (Clone: 36; Catalog # 790-4497; Vendor: Ventana, Tucson, AZ;

Antibodies used	Dilution: ready to use [RTU]; Pre-treatment: CC1-S 64 minutes; Detection: Ultraview; Staining platform: Ventana Benchmark Ultra). p120 stain (Clone: 98; Catalog # 610134; Vendor: BD Biosciences, Franklin Lakes, NJ; Dilution: 1:200; Pre-treatment: CC1-S; Detection: Ultraview; Staining platform: Ventana Benchmark Ultra). Beta-catenin (Clone: B-catenin-1; Catalog # M3539, Vendor: Dako, Santa Clara, CA; Dilution: 1:250; Pre-treatment: CC1 24 minutes; Detection: Optiview; Staining platform: Ventana Benchmark Ultra). N-terminus E-cadherin antibody (Clone: 36B5; Catalog # PA0387, Vendor: Leica Biosystems, Deer Park, IL; Dilution: RTU; Pre-treatment: ER2 20 minutes; Detection: DAB polymer refine; Staining platform: Leica Bond III).
Validation	Commercially available antibodies used for immunohistochemistry, which were validated in our CLIA approved laboratory.

Clinical data

Policy information about clinical studies

All manuscripts should comply with the ICMJE guidelines for publication of clinical research and a completed CONSORT checklist must be included with all submissions.

Clinical trial registration	N/A
Study protocol	N/A
Data collection	N/A
Outcomes	N/A
