# Supplementary material for: Clinicopathologic and genomic features of lobular like invasive mammary carcinoma: is it a distinct entity?
Source: NPJ Breast Cancer. 2023 Jul 13;9:60. doi: 10.1038/s41523-023-00566-7 (PMC10345141; doi:10.1038/s41523-023-00566-7)
Supplement: Supplementary file 3 — Data Set 1 [file 41523_2023_566_MOESM3_ESM.pdf]

Supplementary Data Sets

Kaplan-Meier Survival analysis using log rank test

Although LLIMCas appeared to harbor an intermediate survival between IDCs and ILCs, statistical significance was not reached, even when LLIMCas were separately compared to ILCs or IDCs. ILCs, however, were found to have a worse RFS ($p=0.047$) and DRFS ($p=0.026$) compared to IDCs (see KM survival curves below). Next, LLIMCas were combined with IDCs or ILCs, and grouped survival was compared to ILCs or IDCs alone, respectively. No statistically significant differences were identified in either set of comparisons (see KM survival curves below). The survival functions were then examined for the entire cohort for known prognostic variables and ME2. RFS, DRFS, and BCSS showed statistically significant differences for tumor grade, pT stage, pN stage, and ME2 score categories but not for nodal status (Table 5 in the manuscript and KM survival curves below). OS showed statistically significant differences for pT stage, pN stage, and ME2 score categories, but not for tumor grade and nodal status (Table 5 in the manuscript and KM survival curves below).

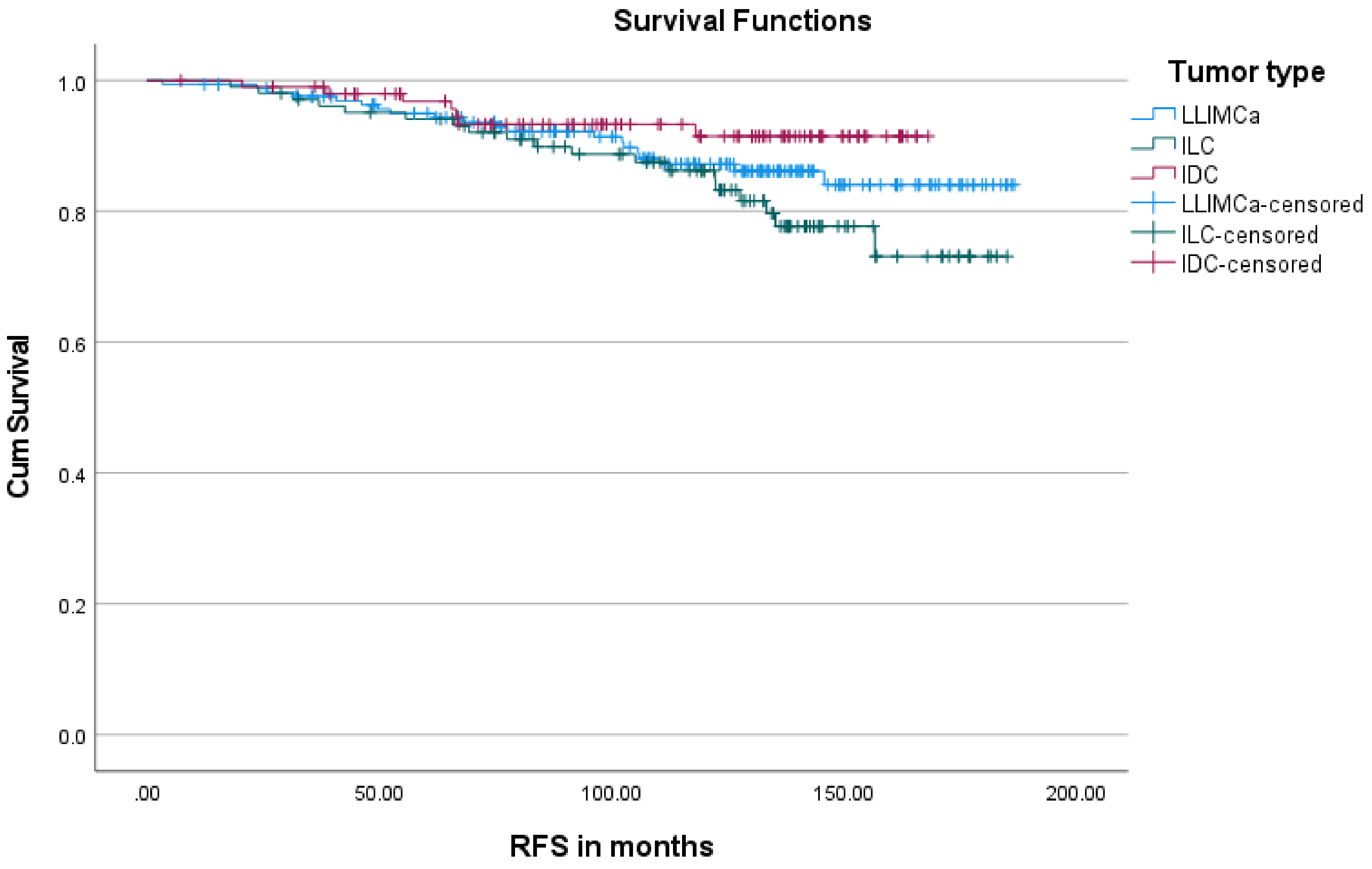

Log rank test p-value: 0.125

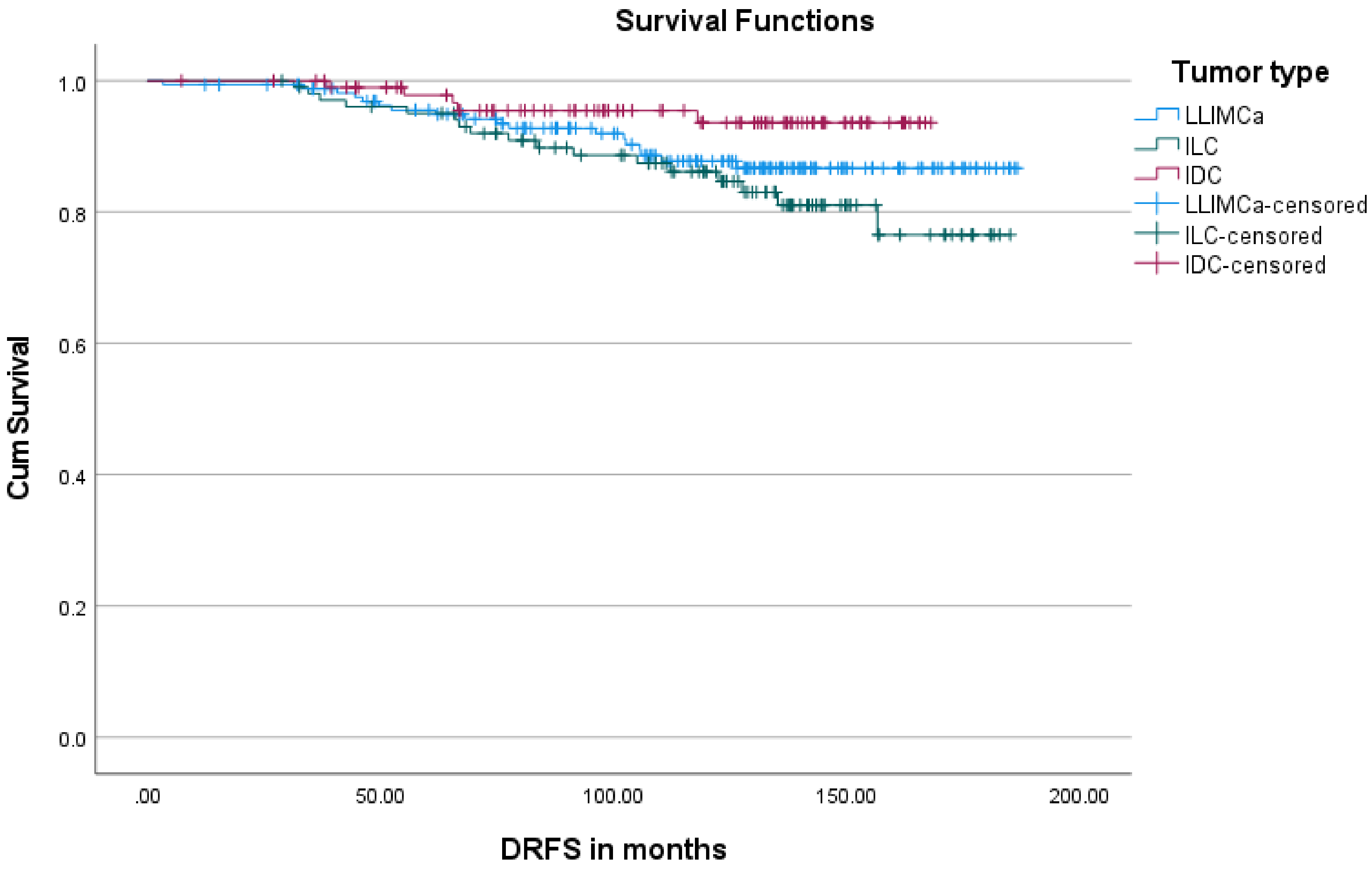

Log rank test p-value: 0.084

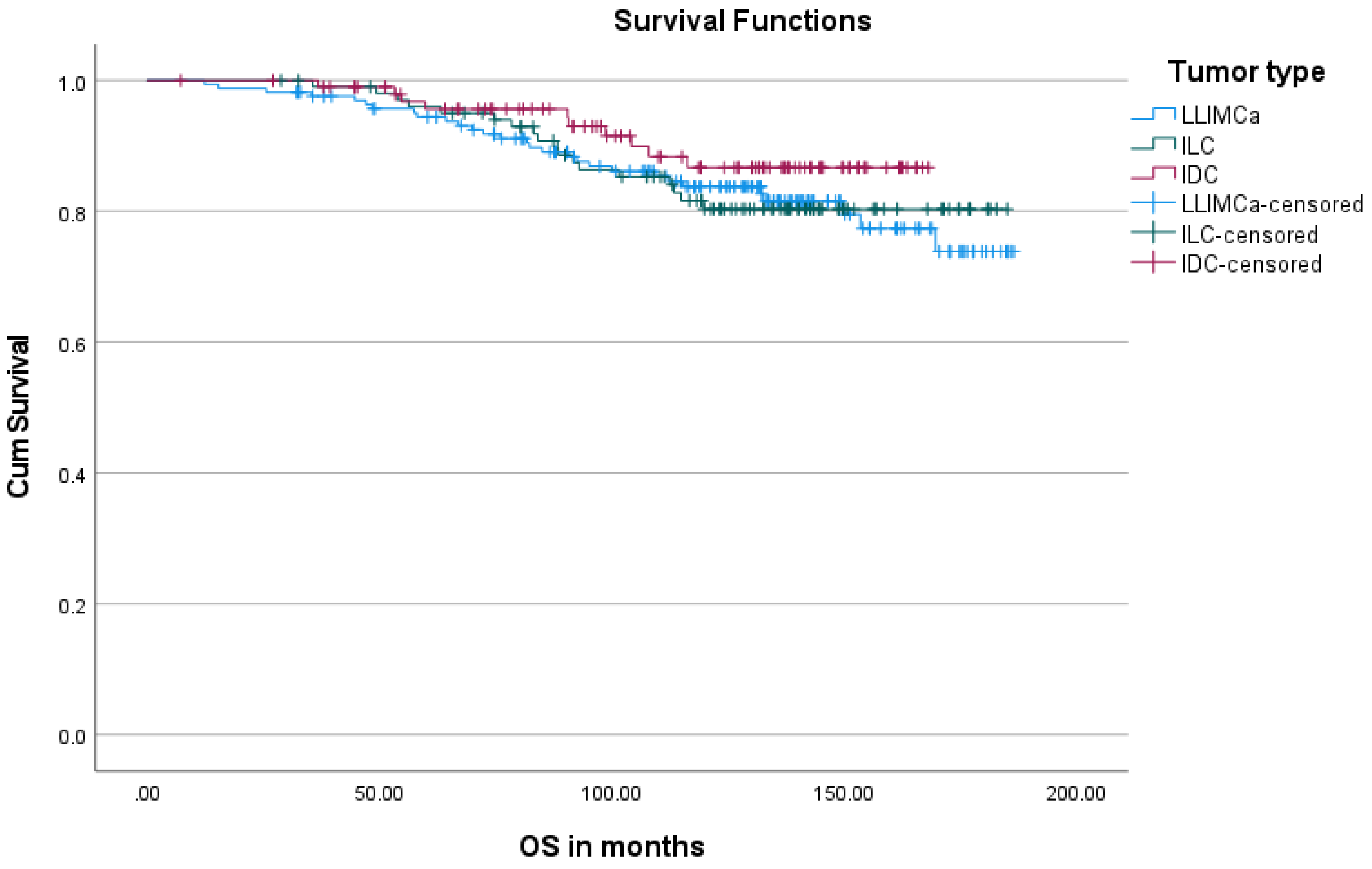

Log rank test p-value: 0.438

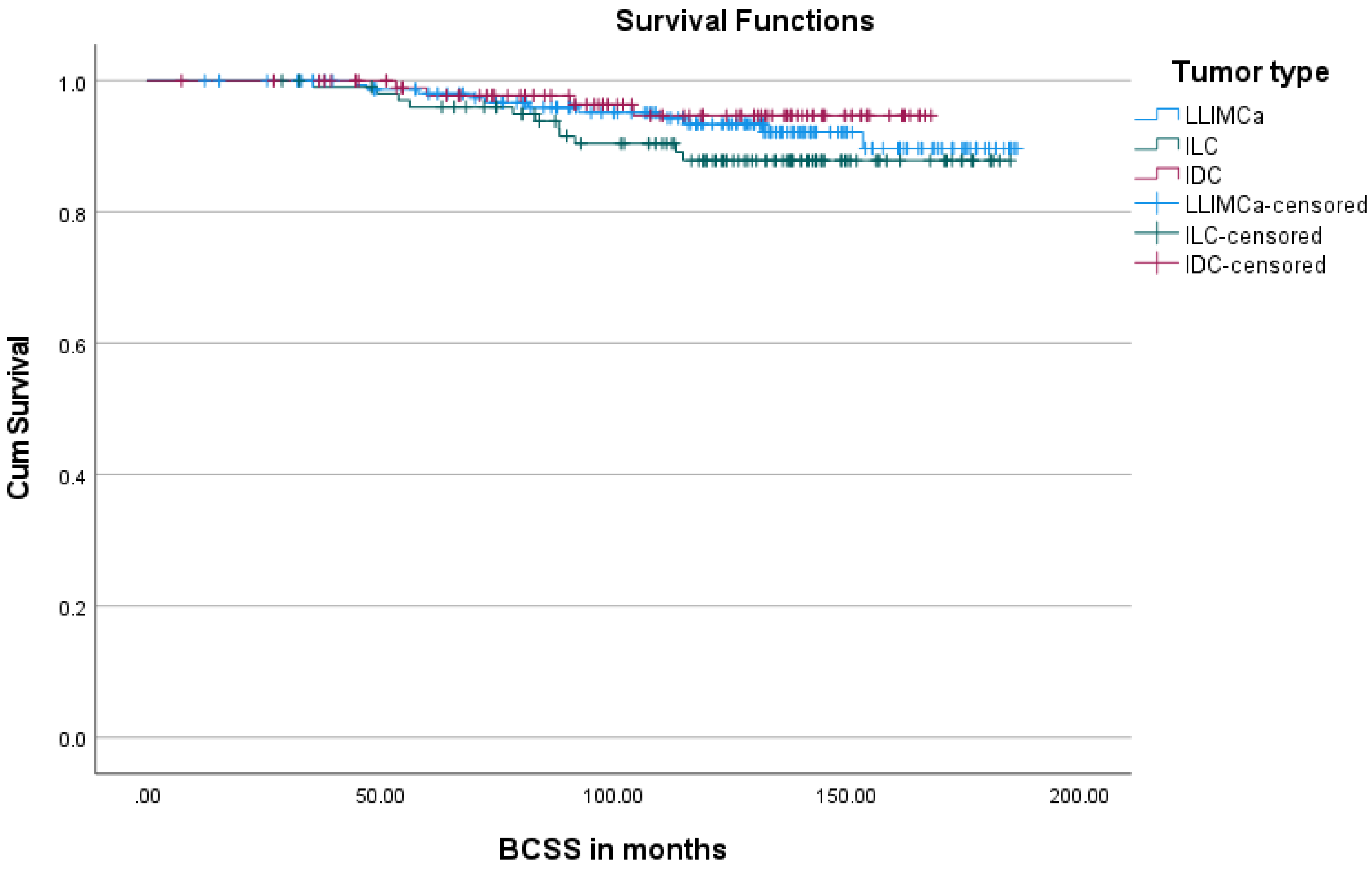

Log rank test p-value: 0.282

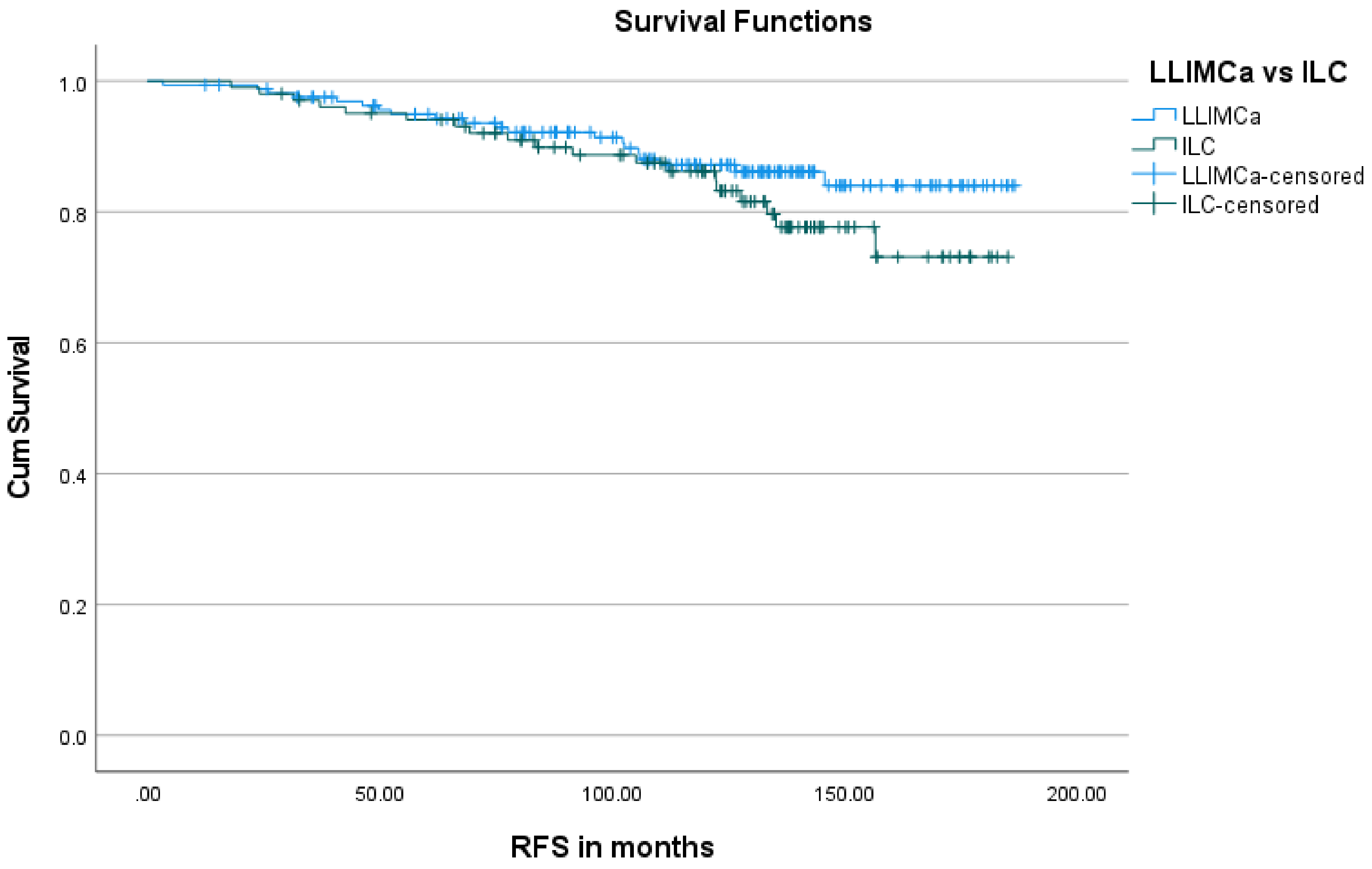

Log rank test p-value: 0.225

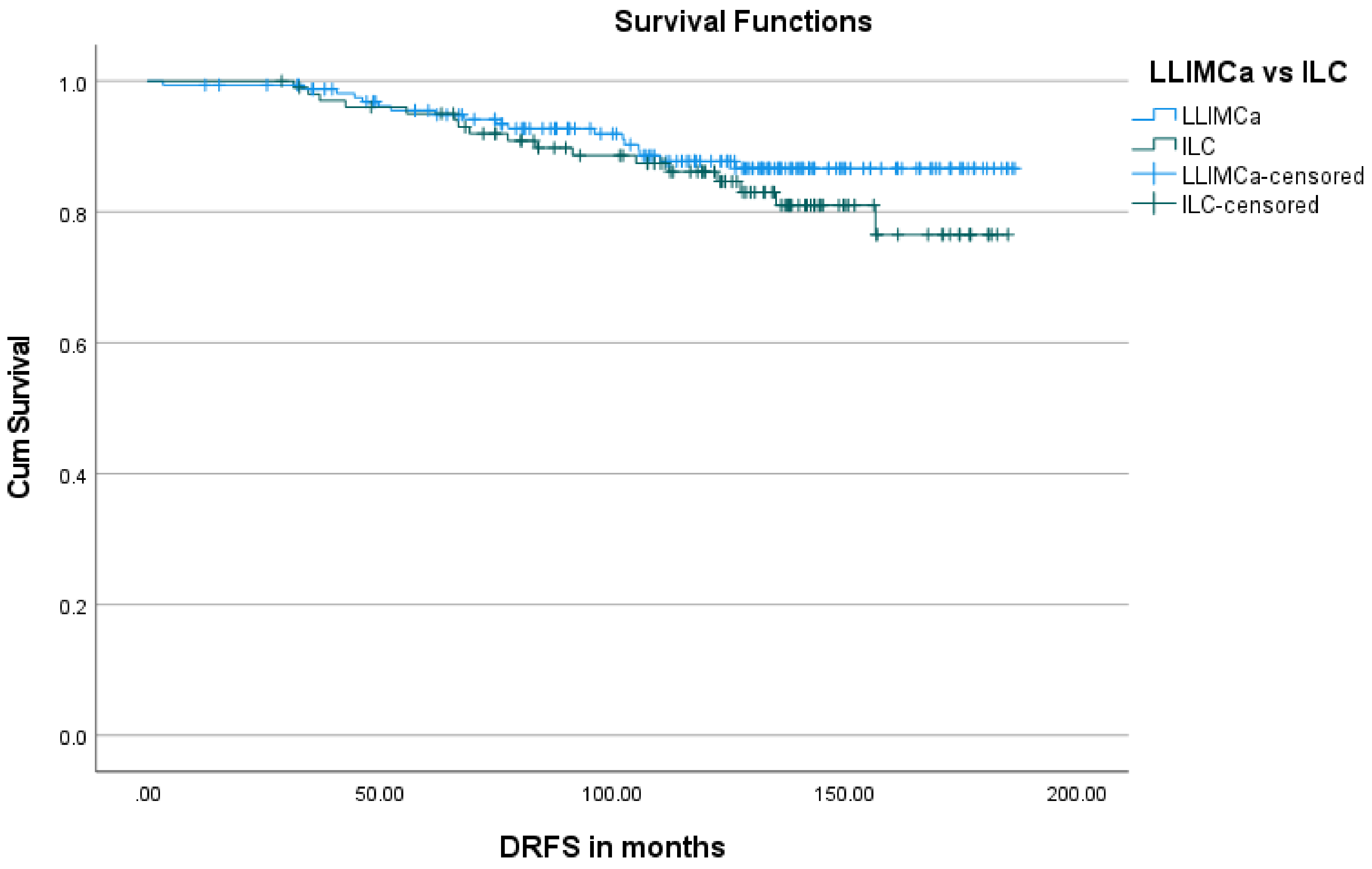

Log rank test p-value: 0.274

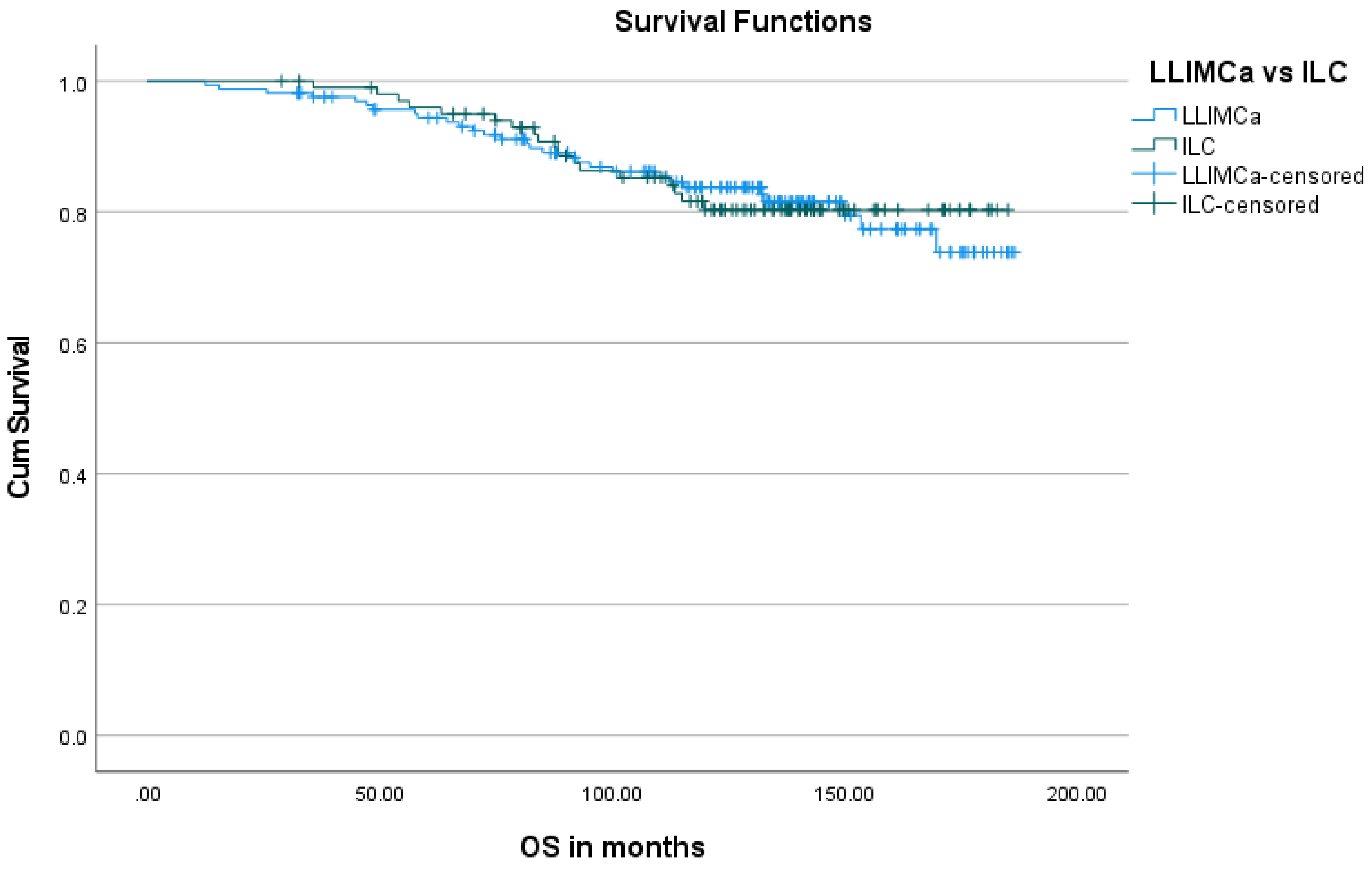

Log rank test p-value: 0.892

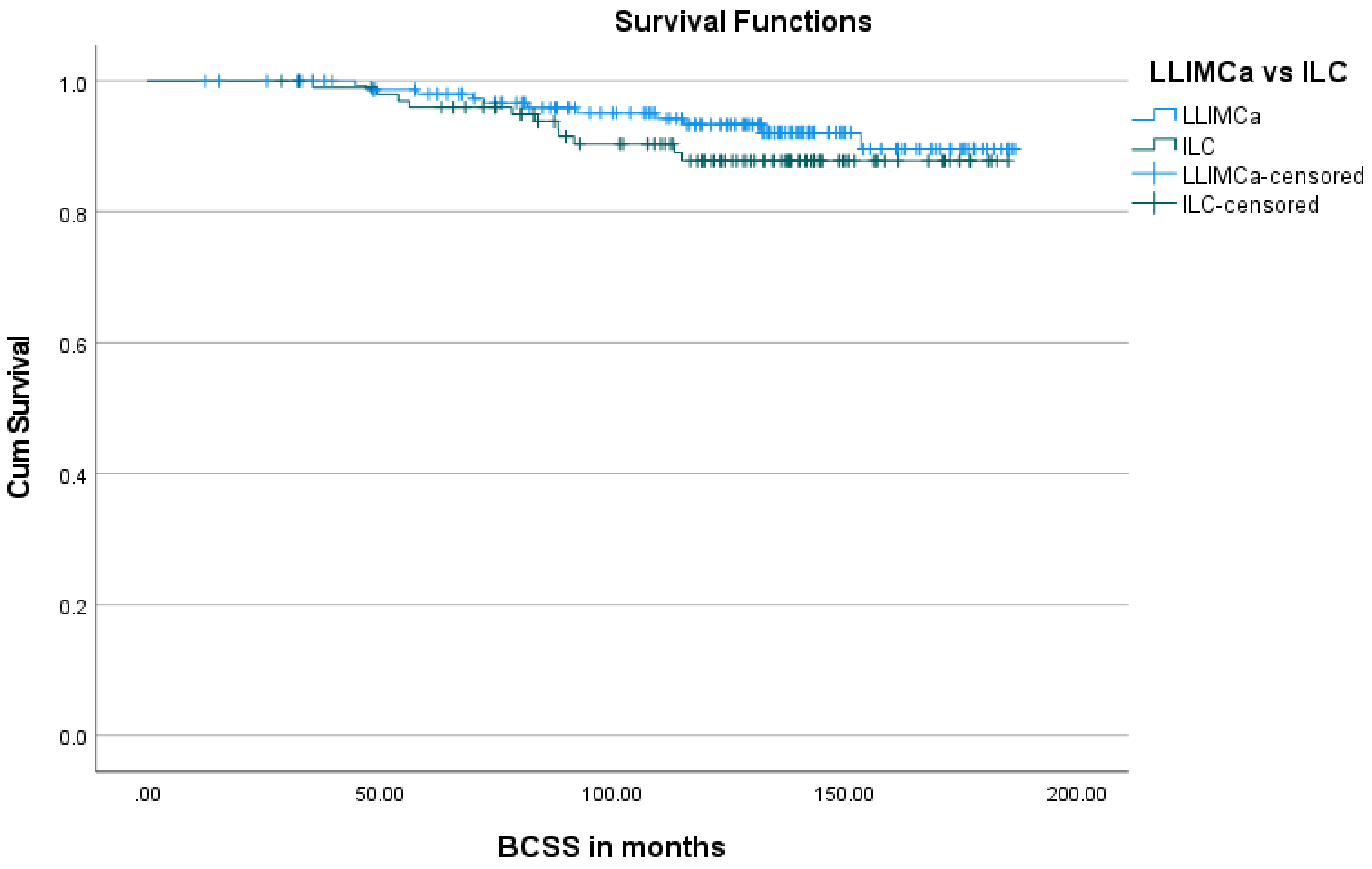

Log rank test p-value: 0.302

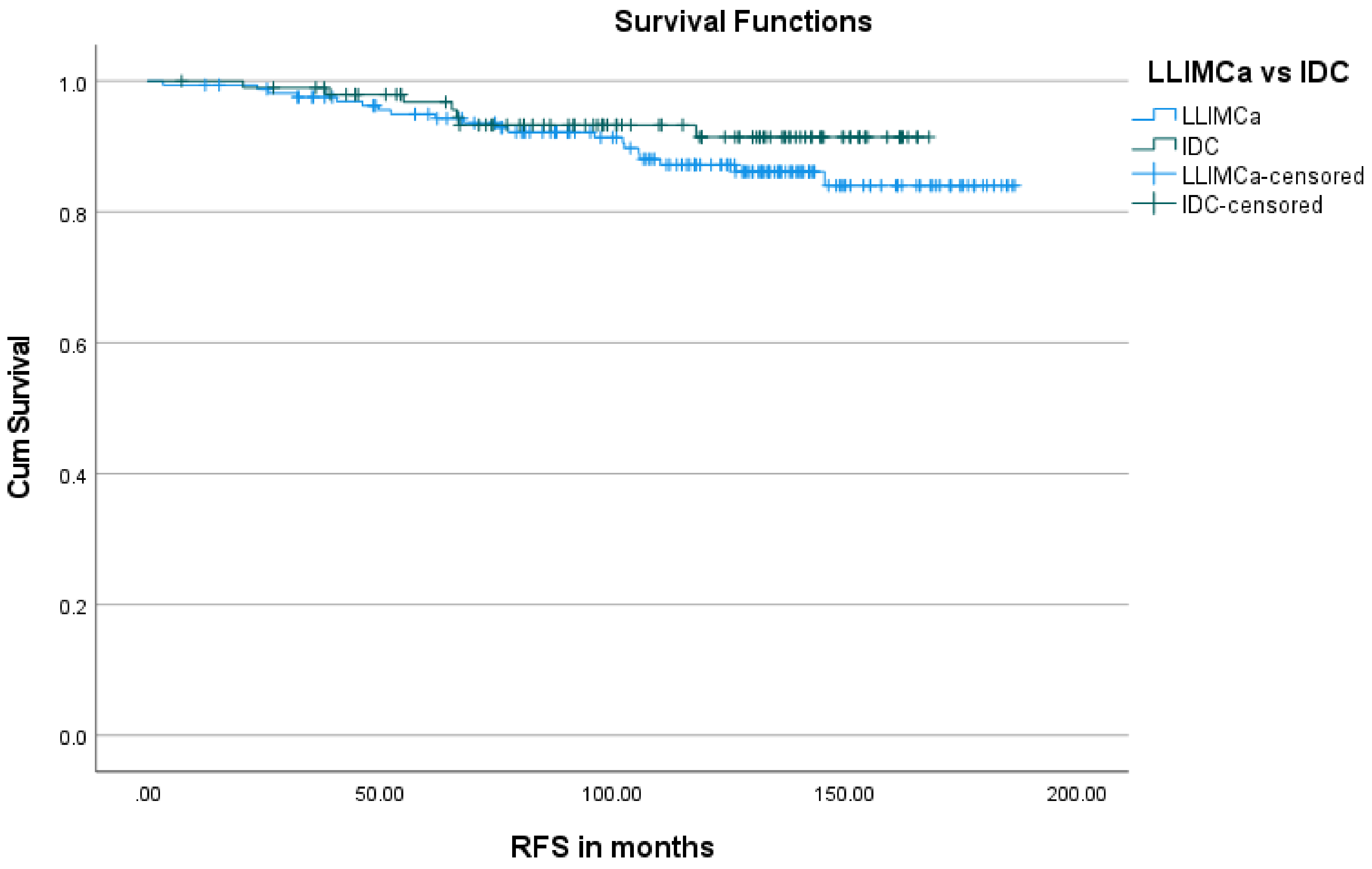

Log rank test p-value: 0.267

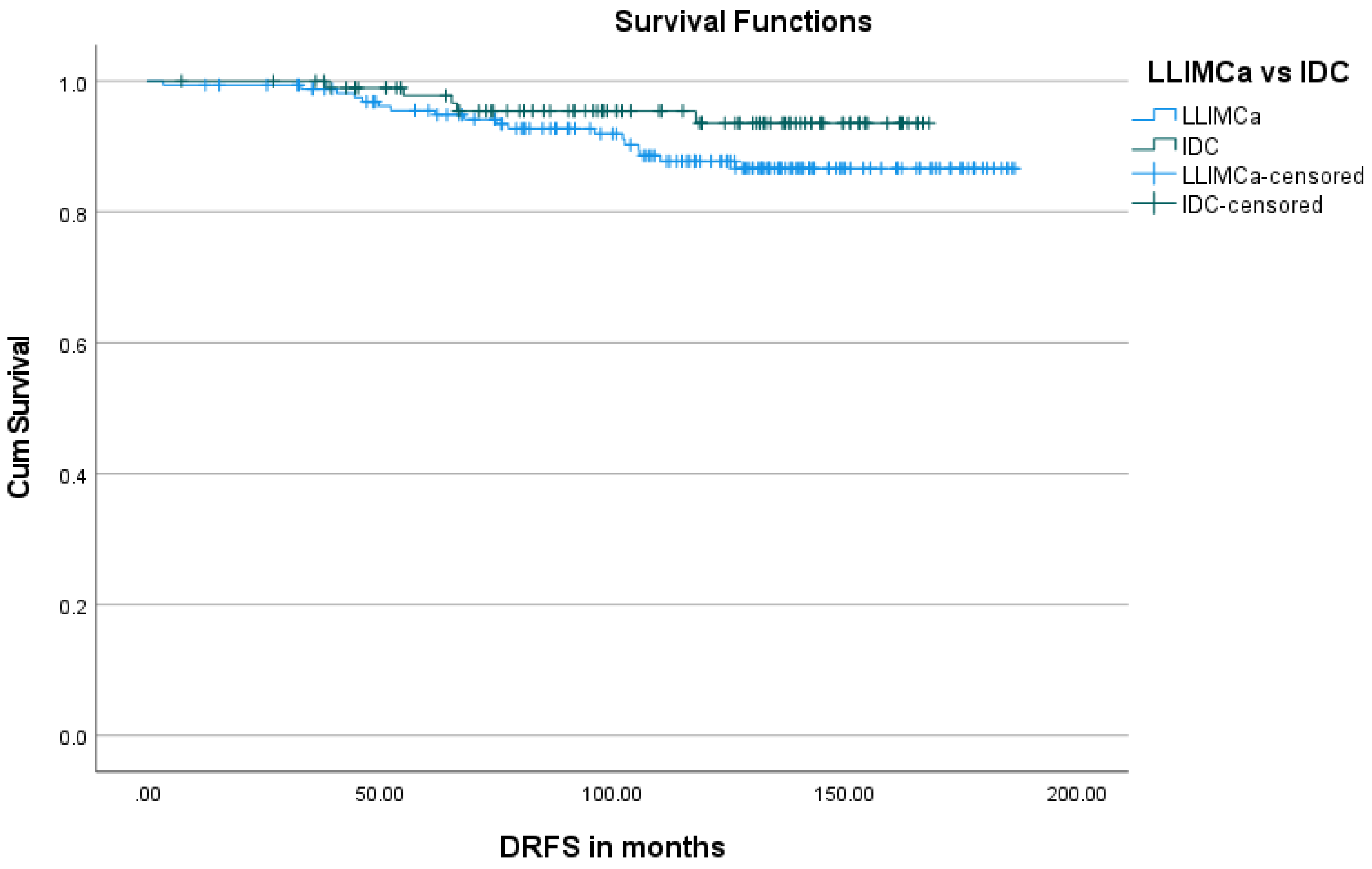

Log rank test p-value: 0.144

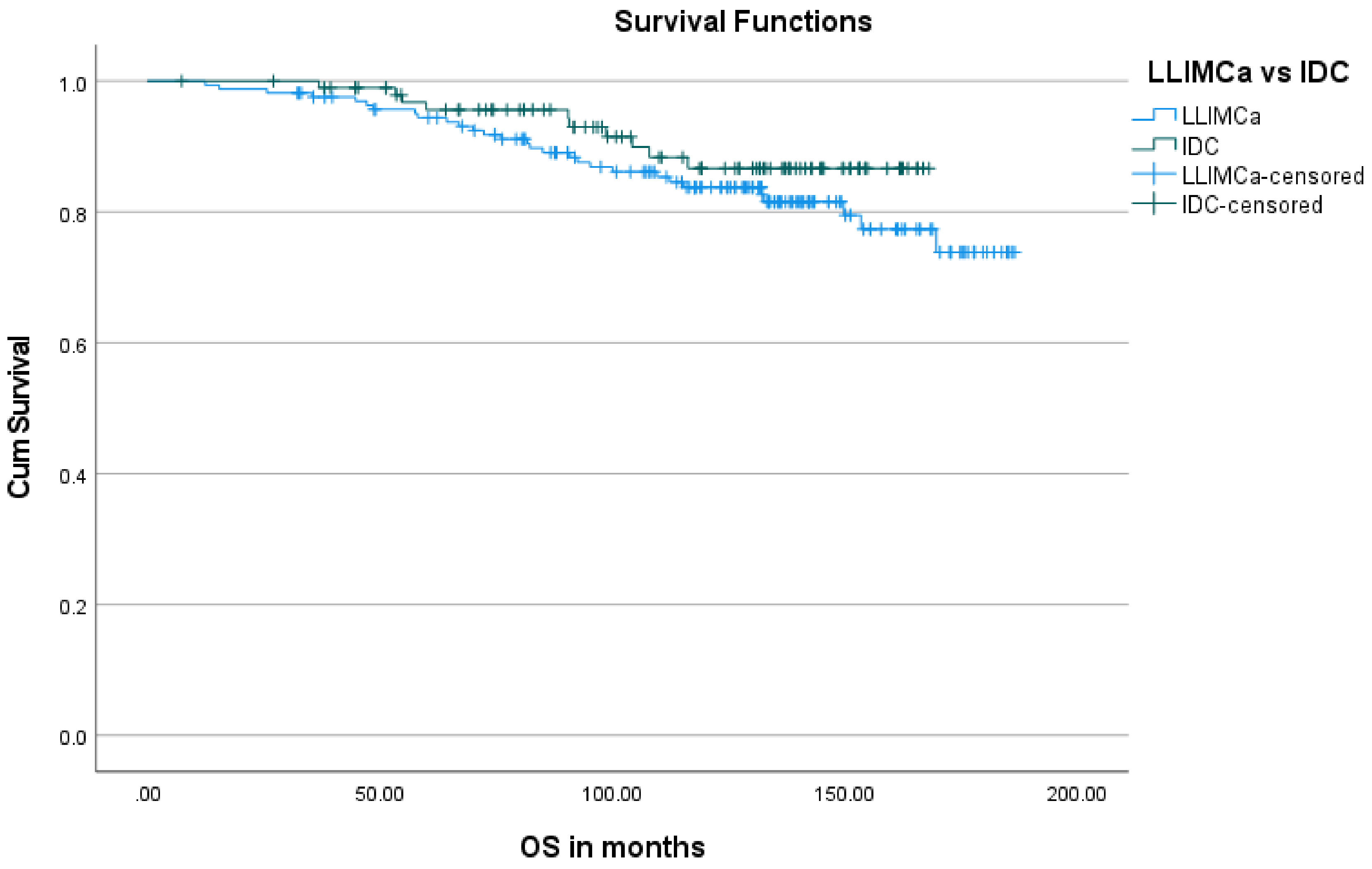

Log rank test p-value: 0.225

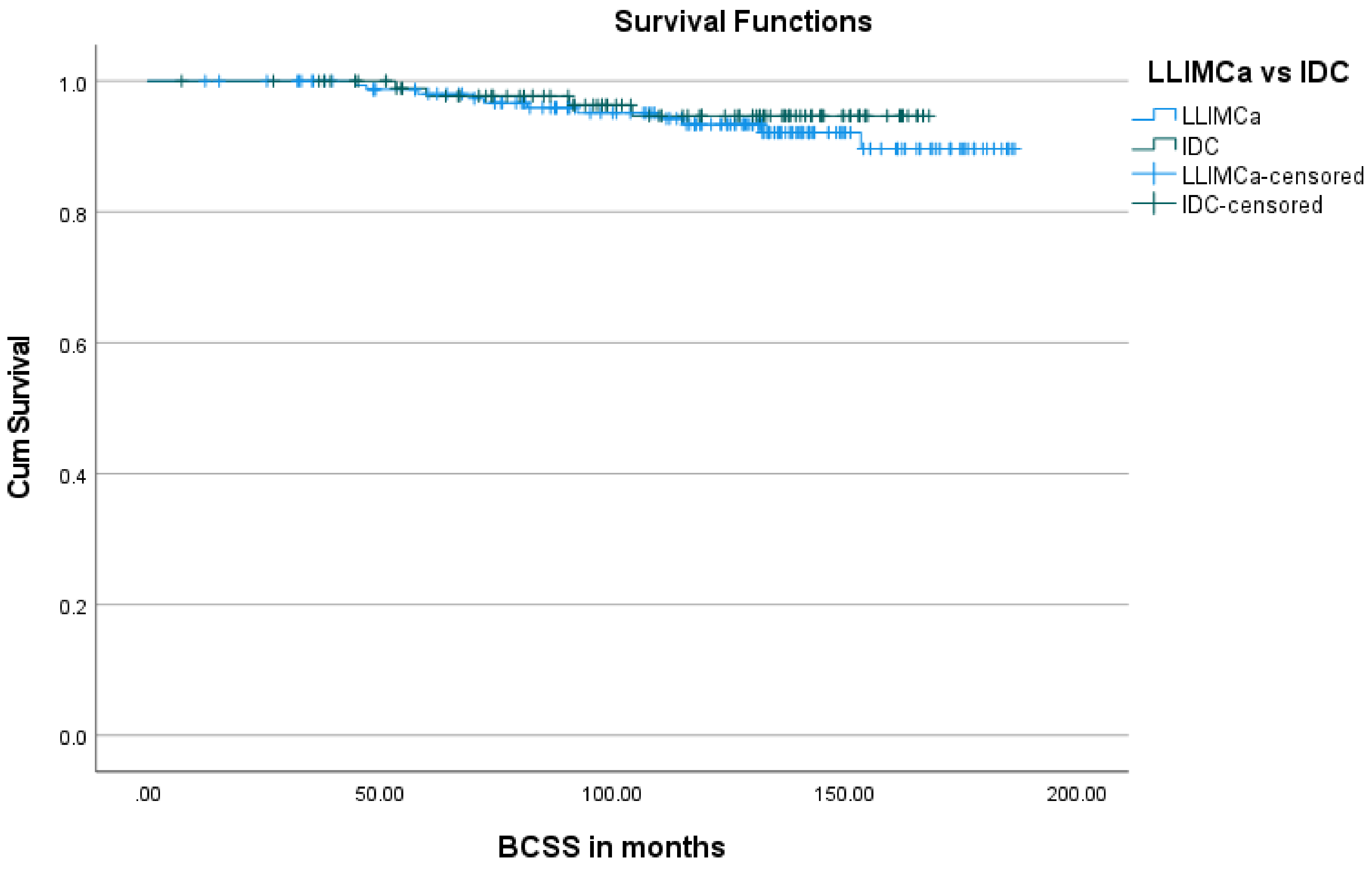

Log rank test p-value: 0.483

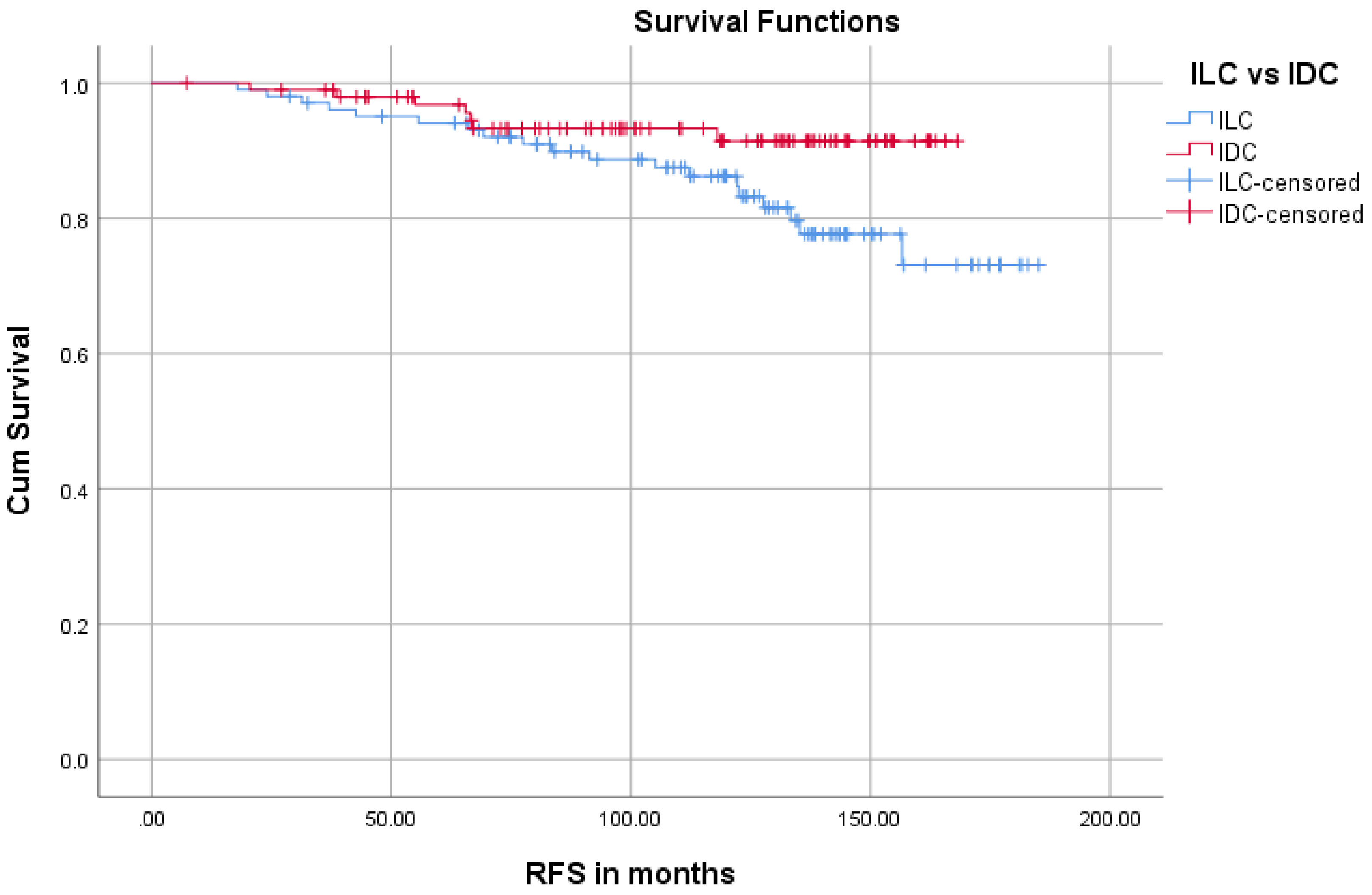

Log rank test p-value: 0.047*

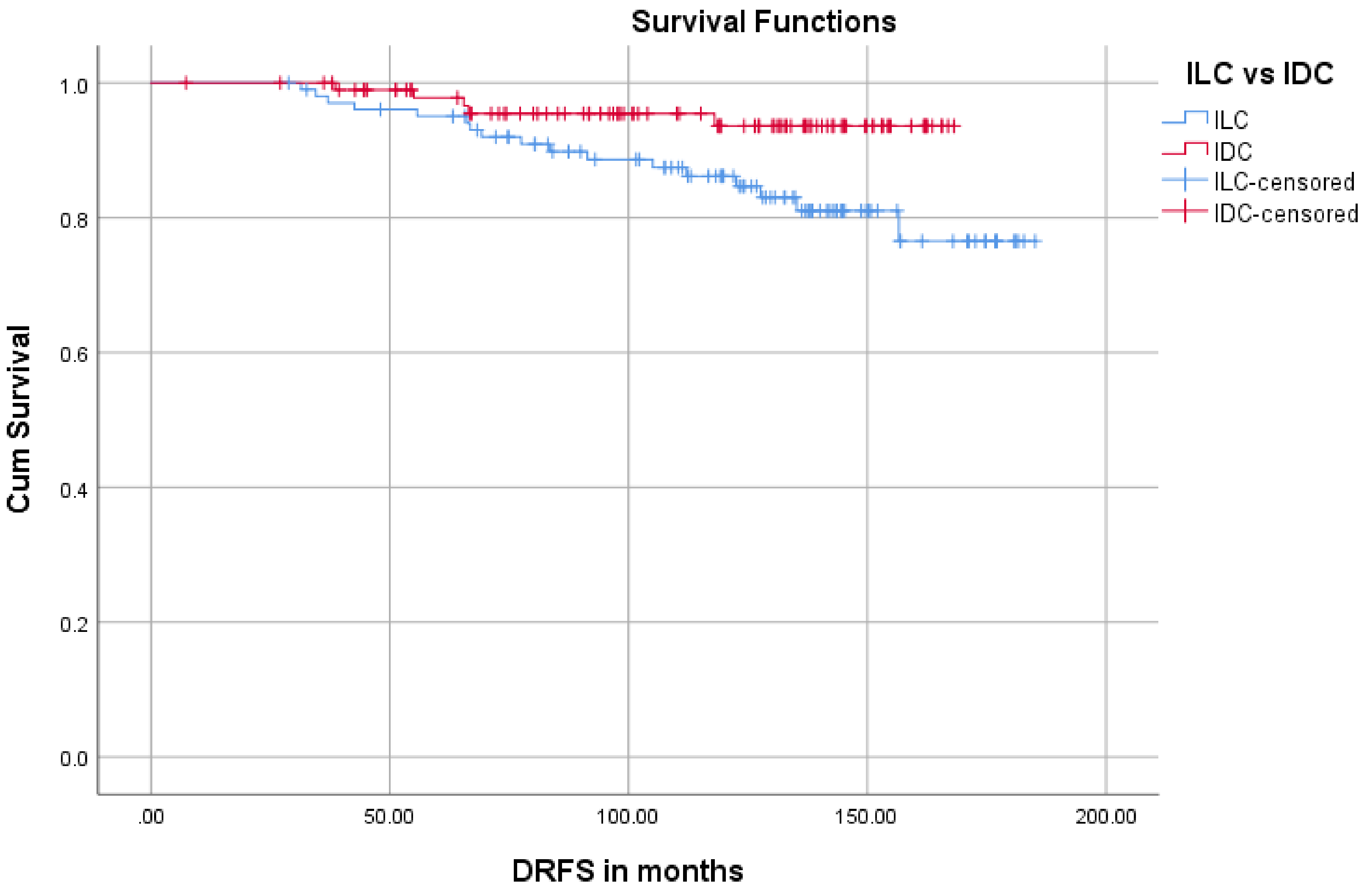

Log rank test p-value: 0.026*

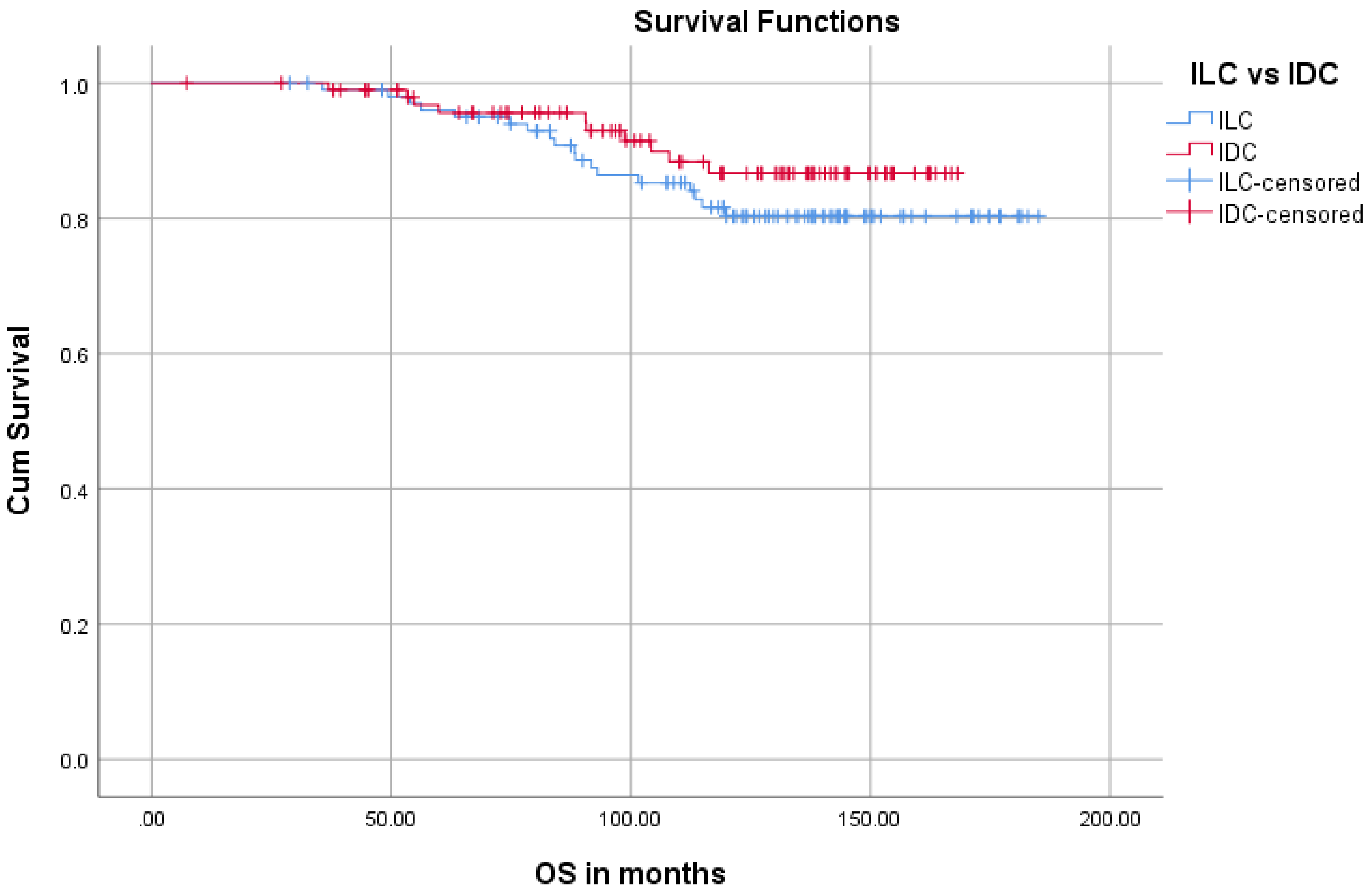

Log rank test p-value: 0.272

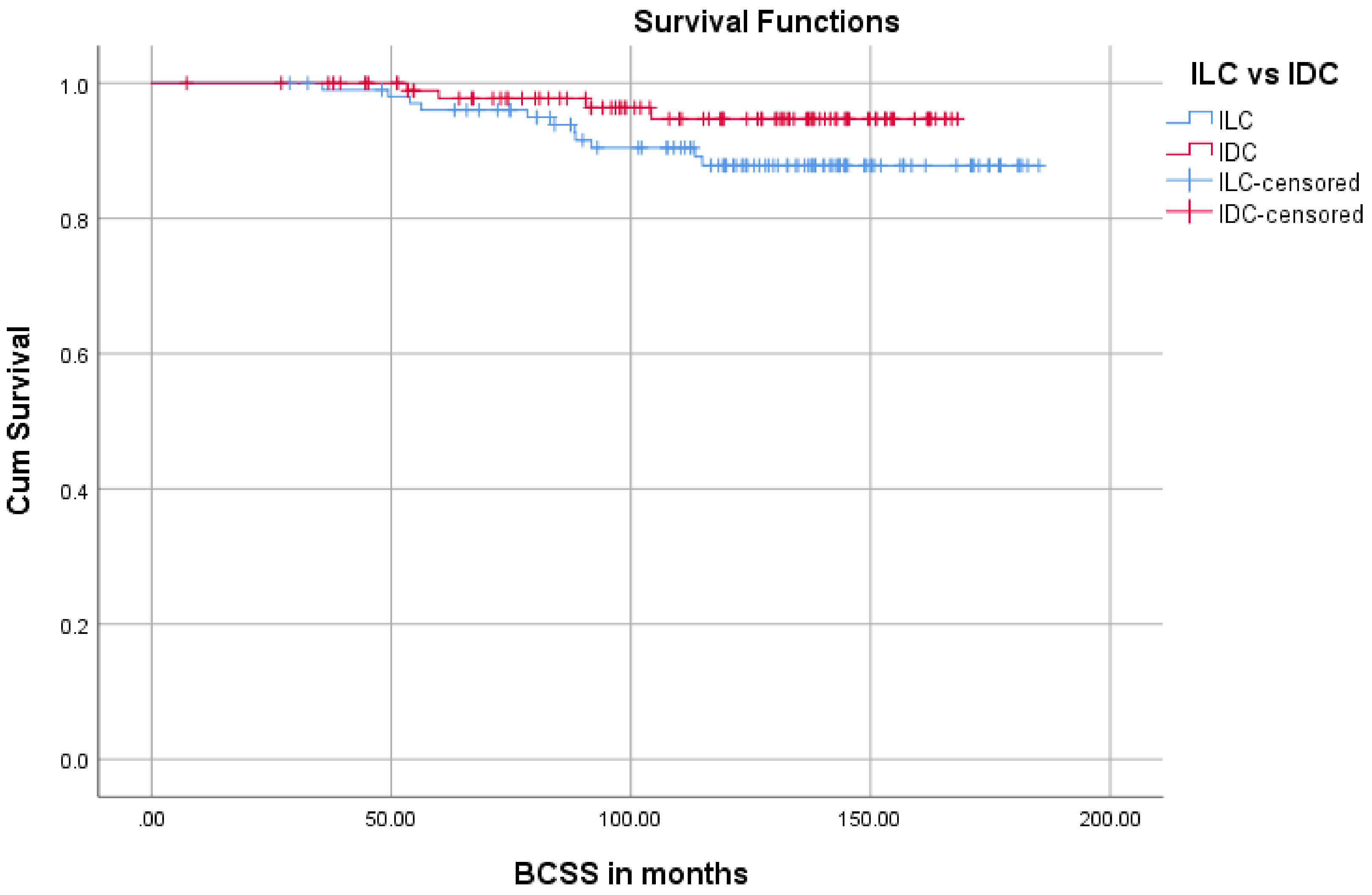

Log rank test p-value: 0.124

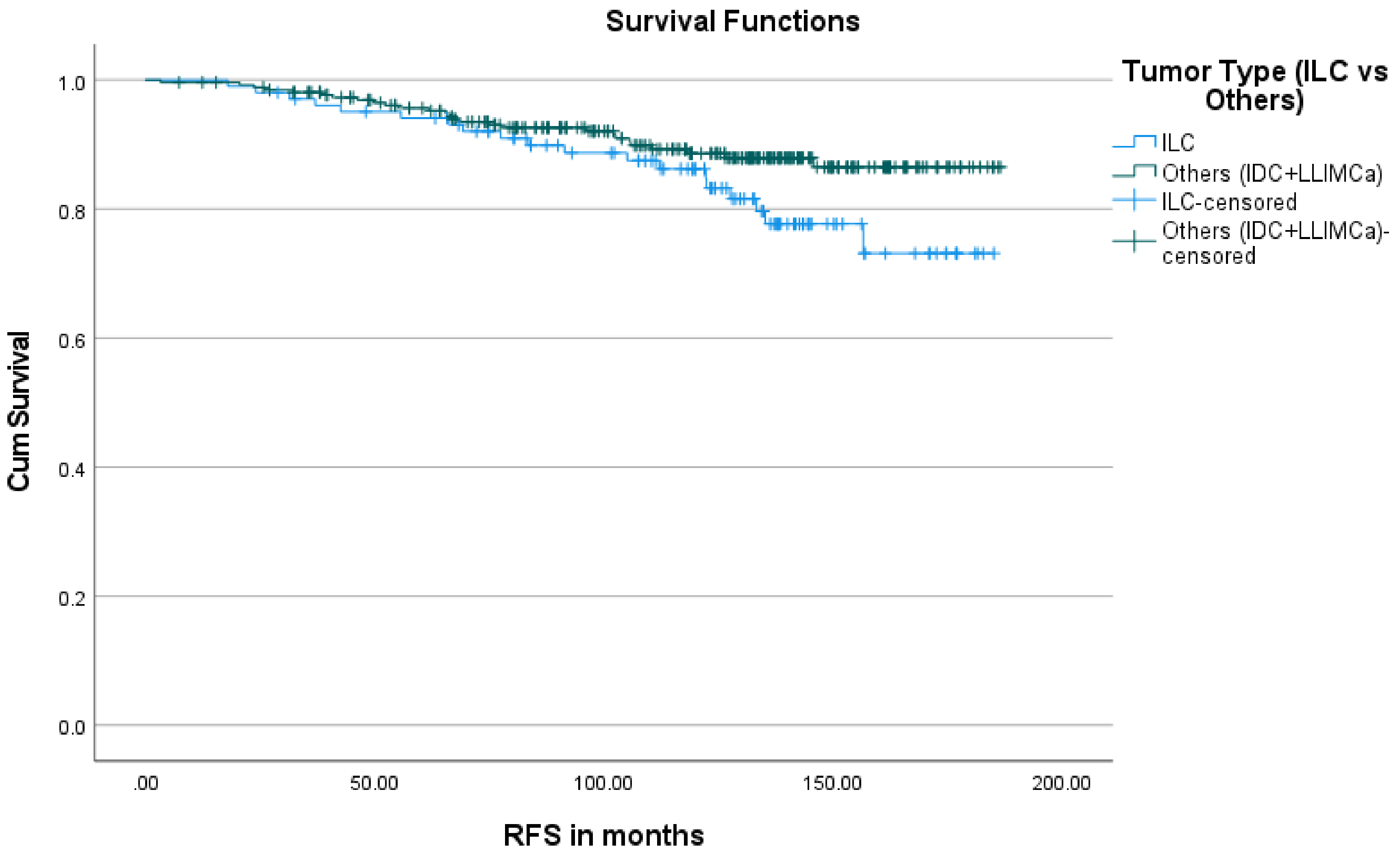

Log rank test p-value: 0.074

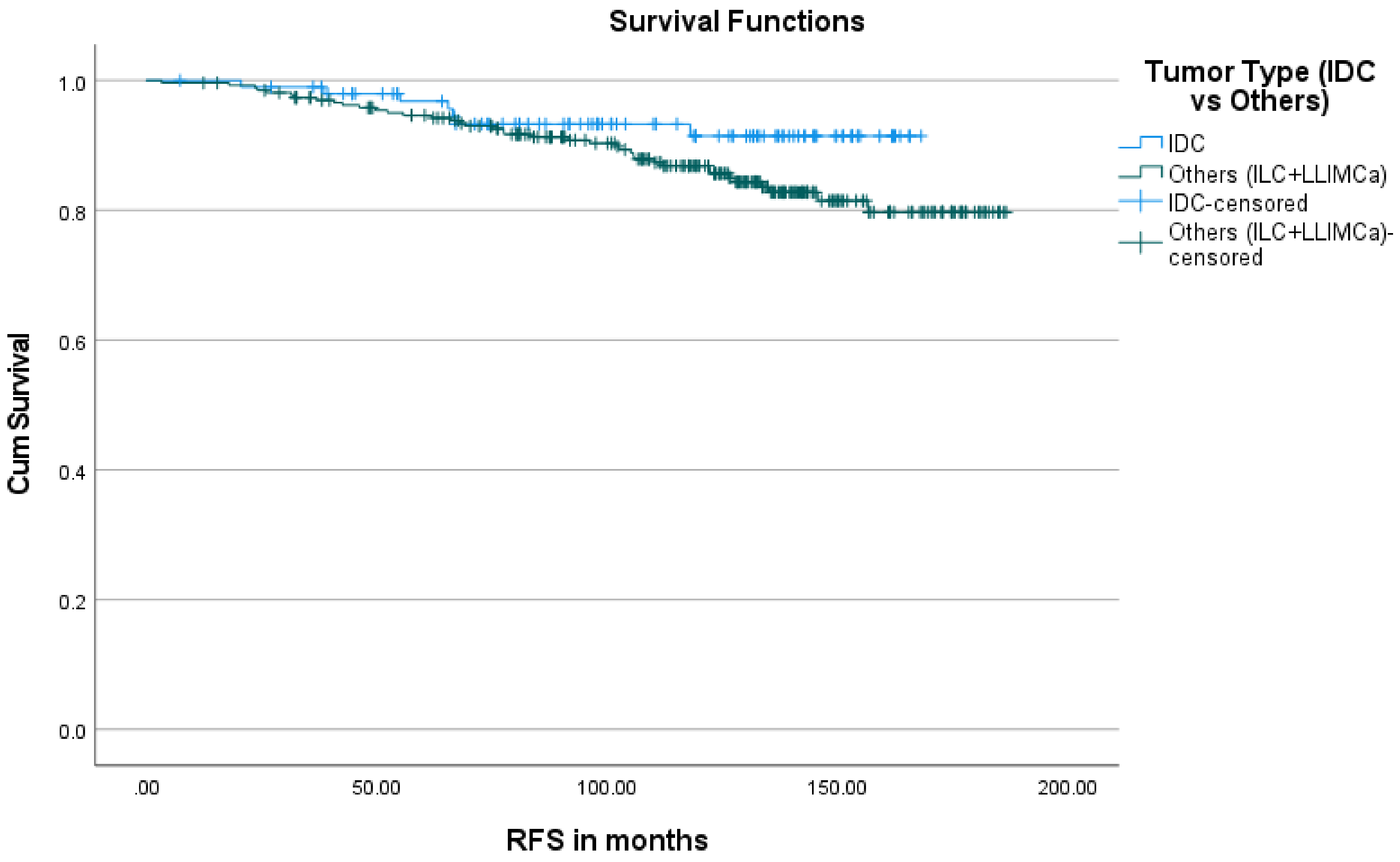

Log rank test p-value: 0.113

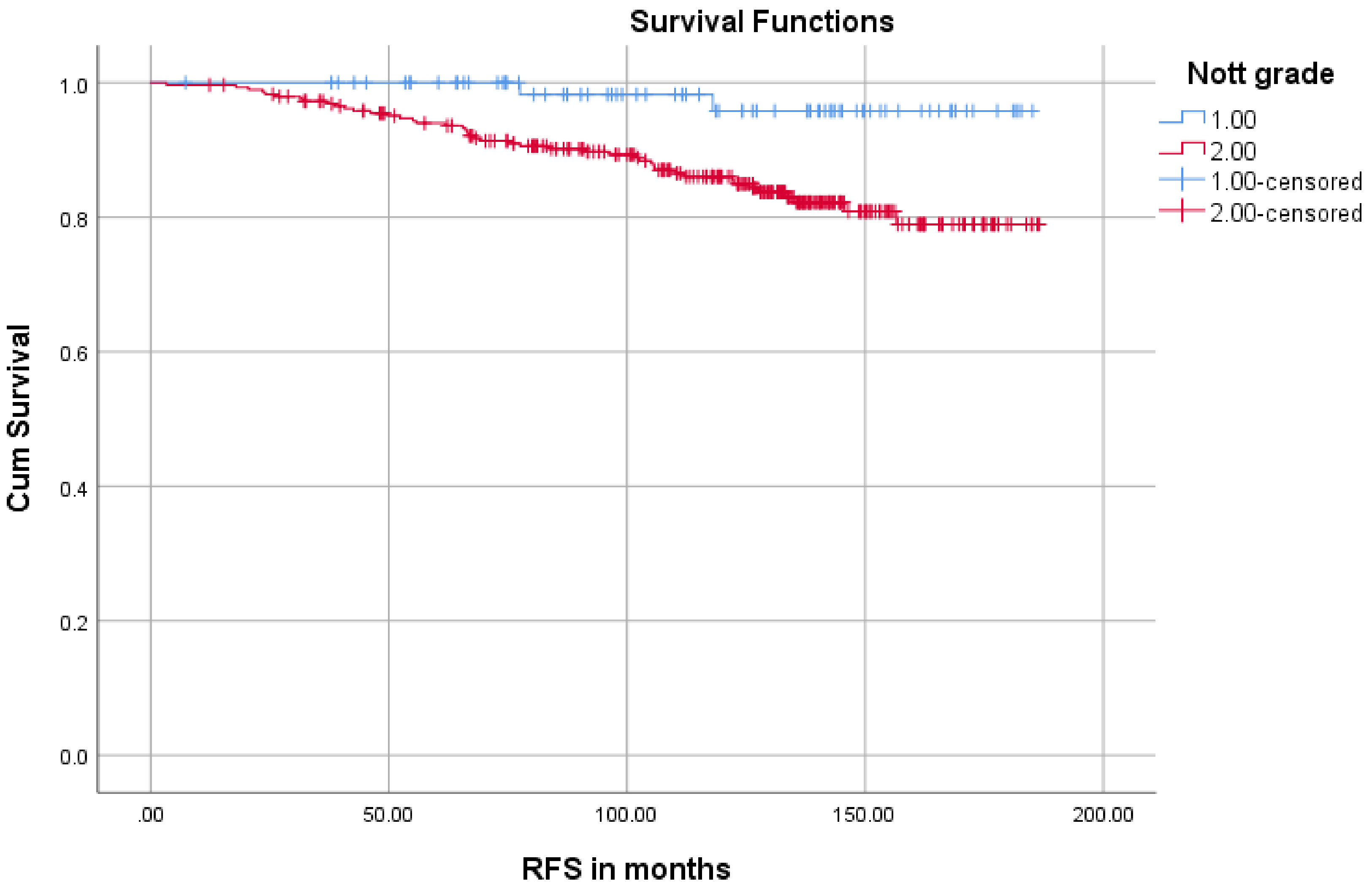

Log rank test p-value: 0.007*

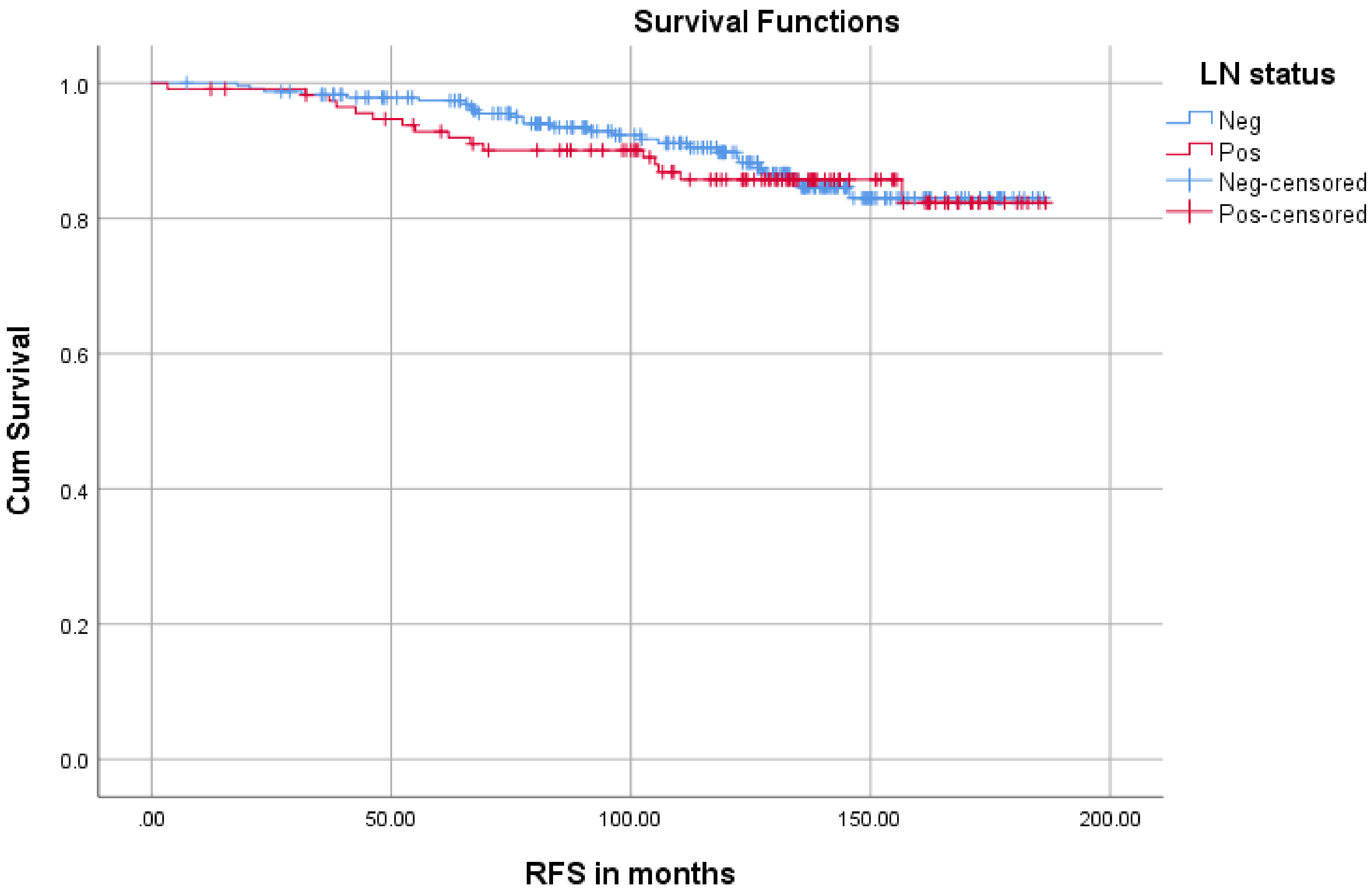

Log rank test p-value: 0.695

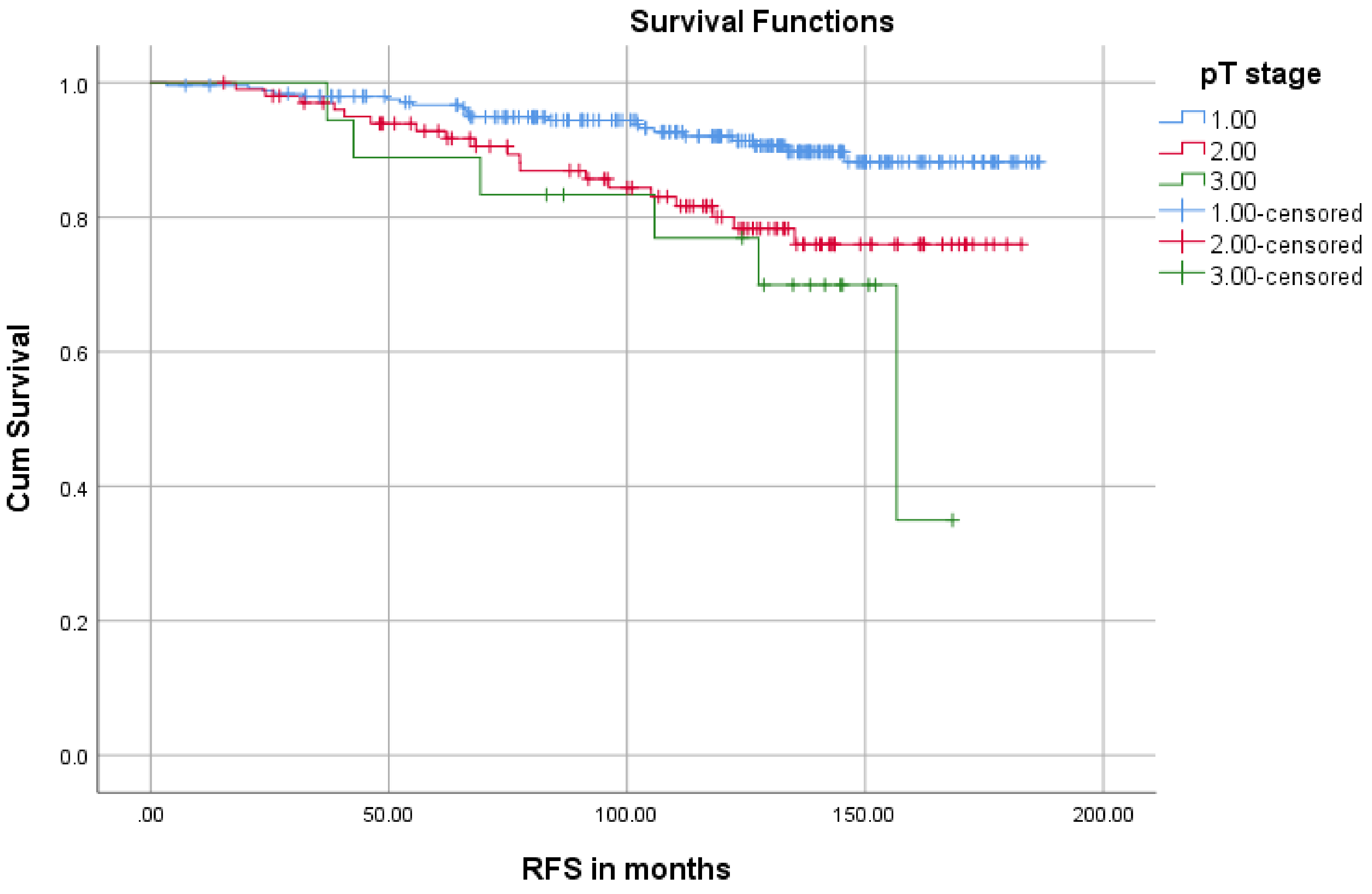

Log rank test p-value: 0.002*

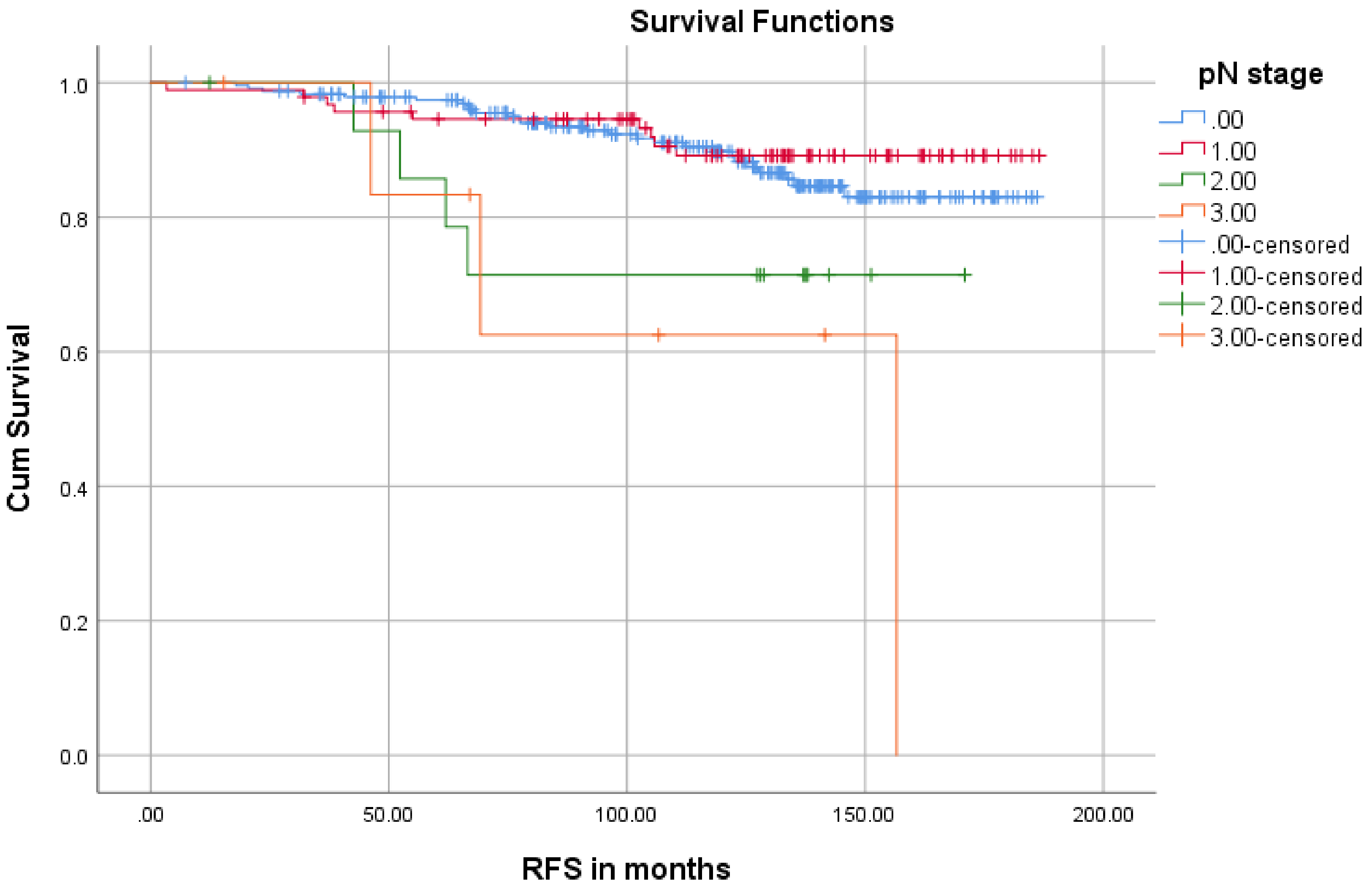

Log rank test p-value: 0.004*

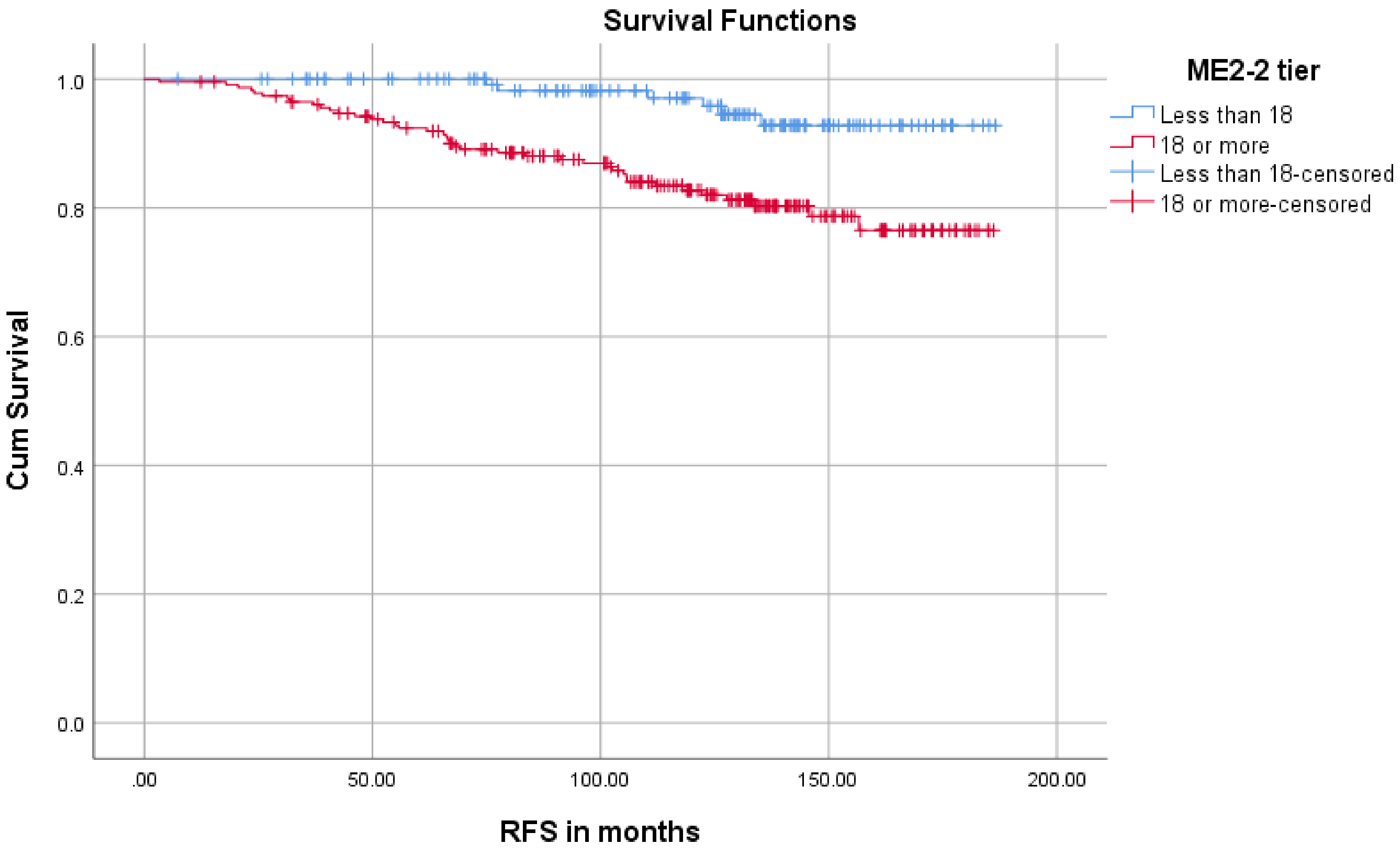

Log rank test p-value: <0.0001*

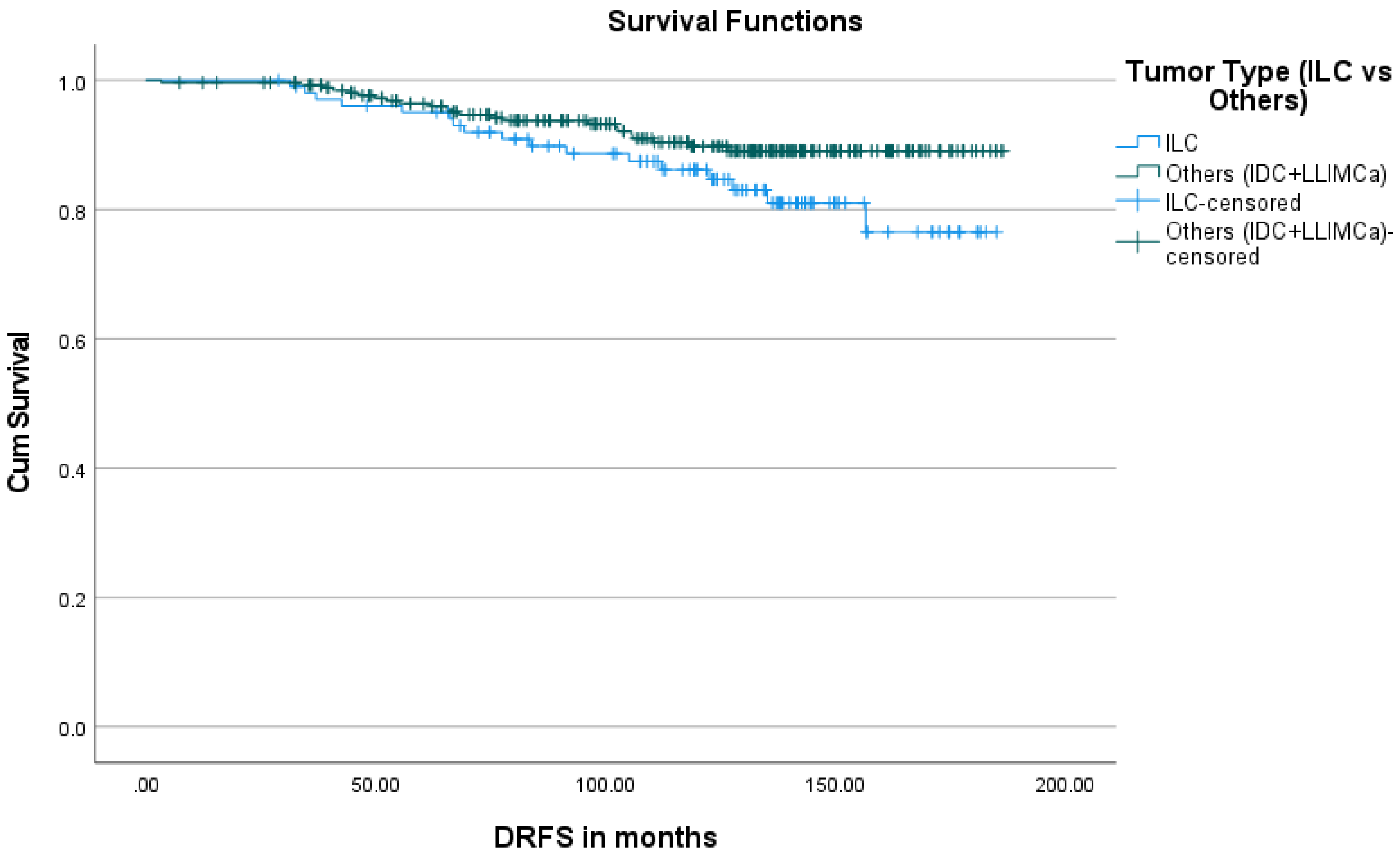

Log rank test p-value: 0.071

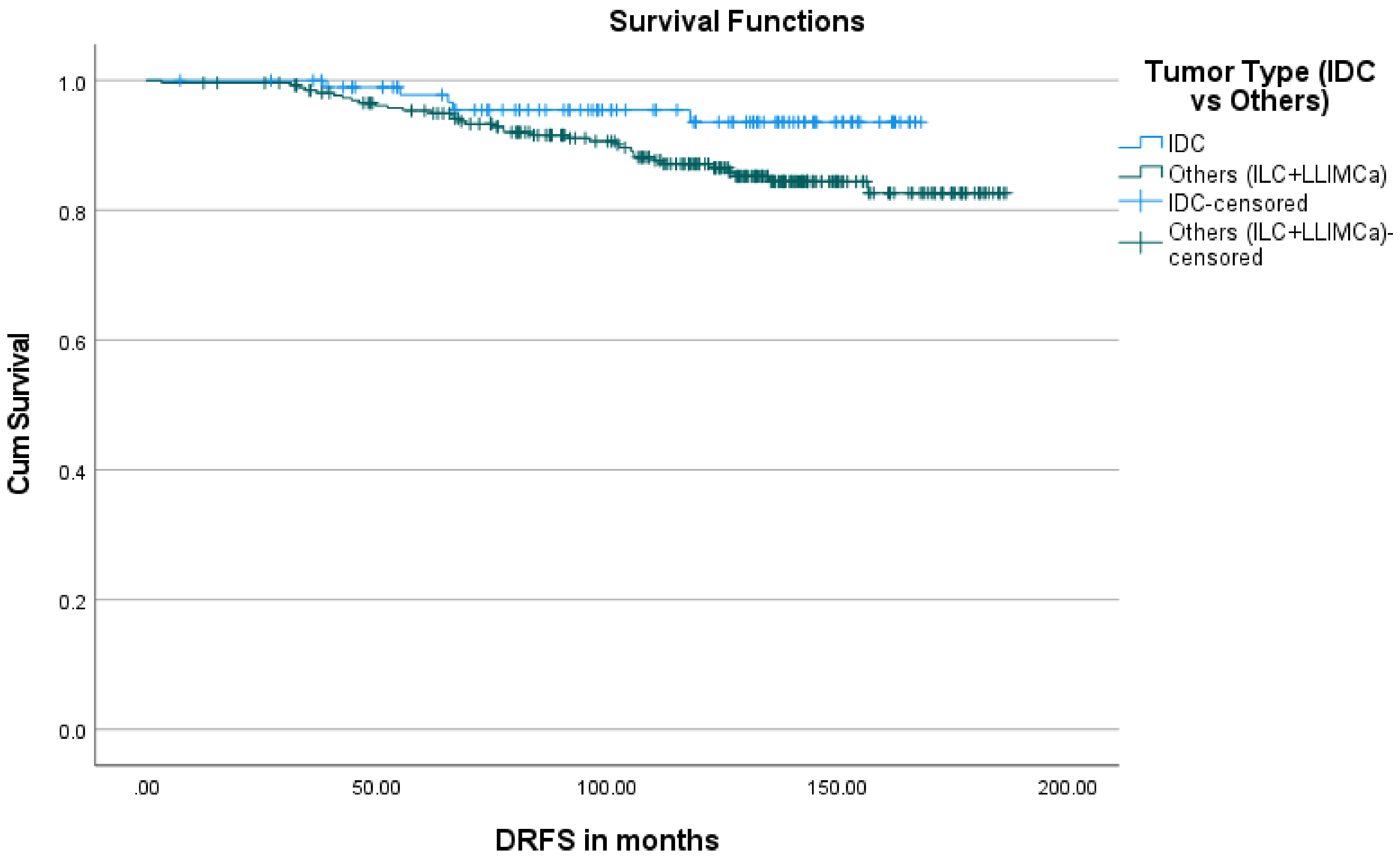

Log rank test p-value: 0.059

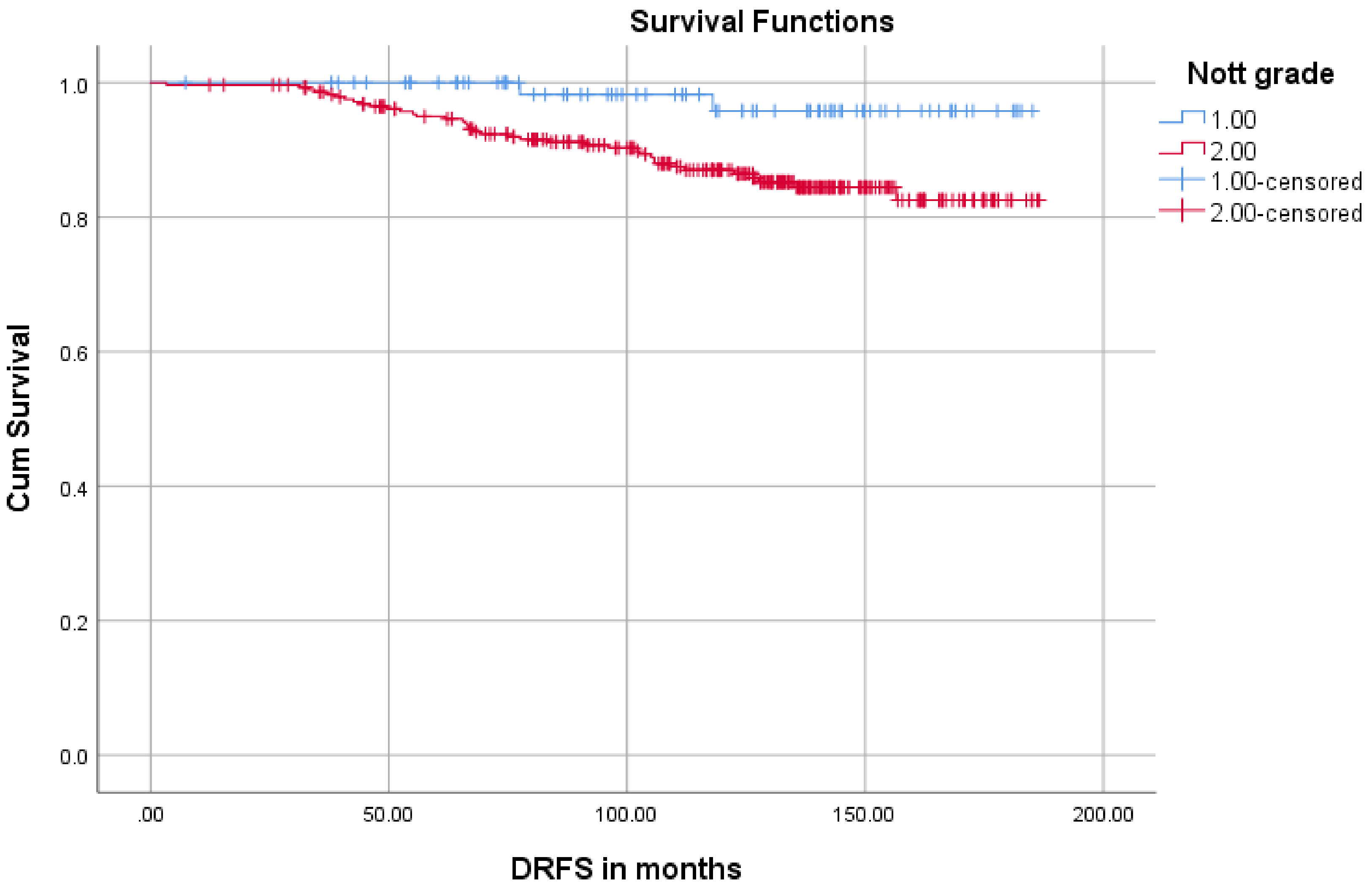

Log rank test p-value: 0.016*

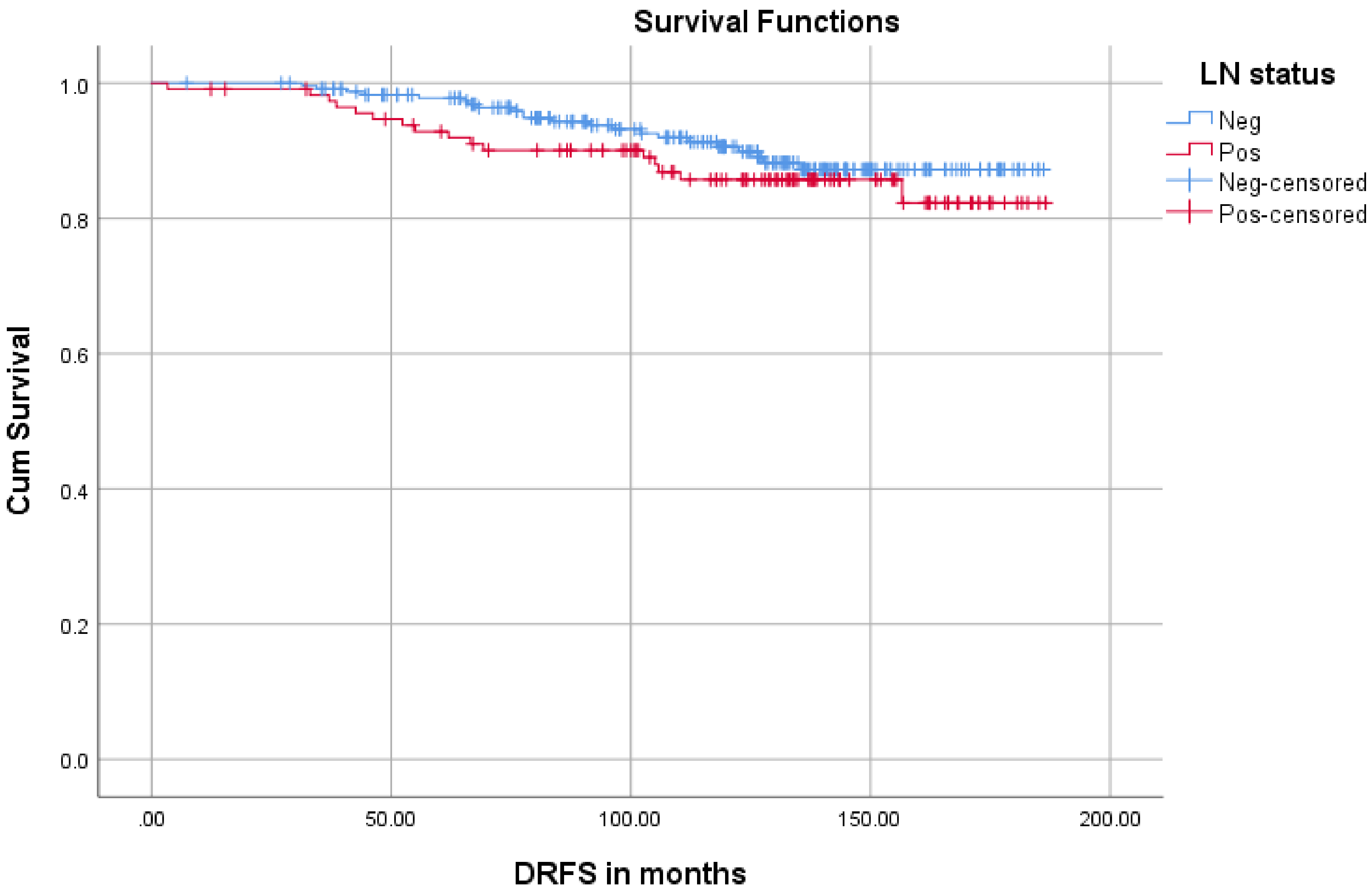

Log rank test p-value: 0.294

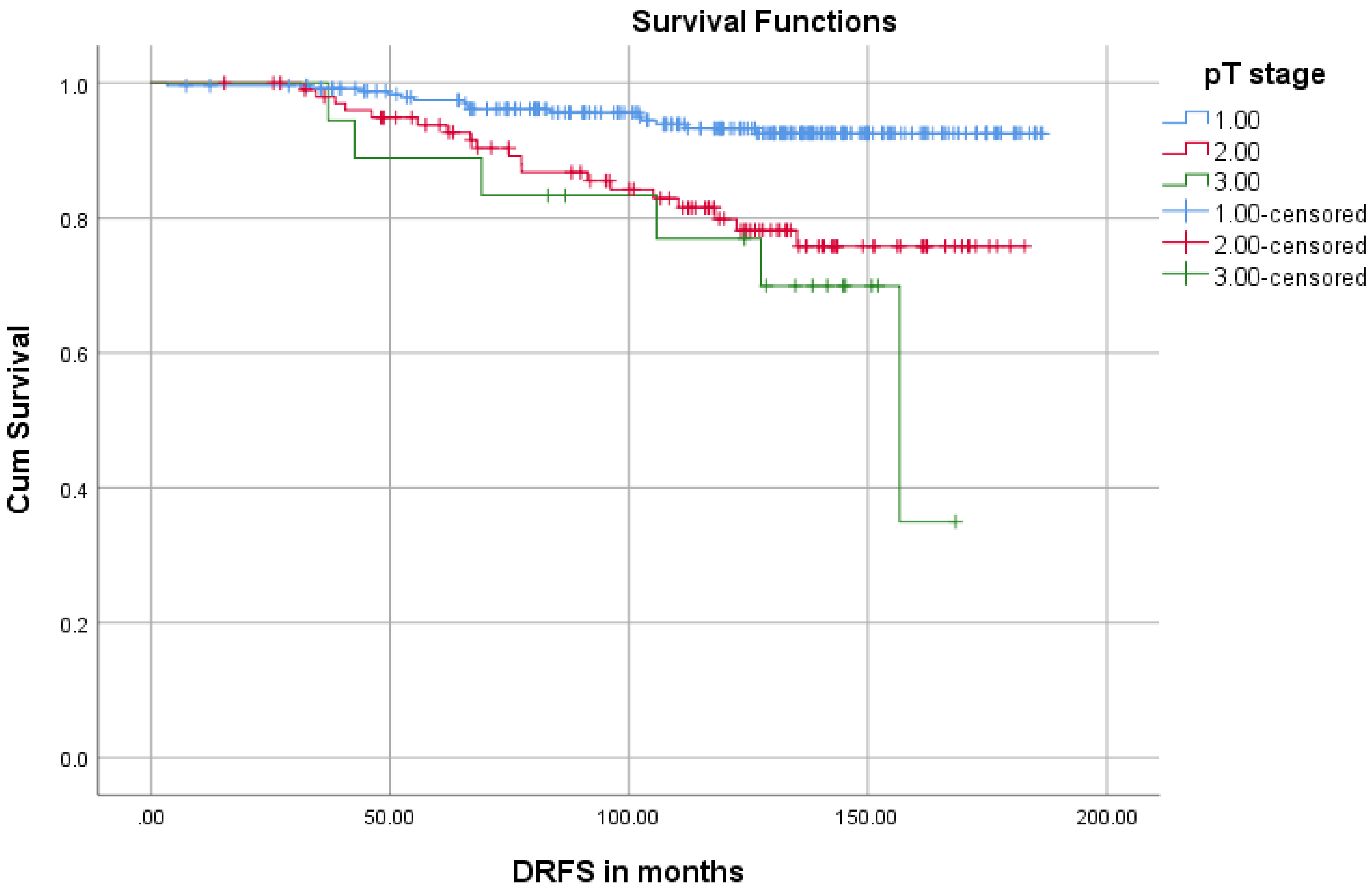

Log rank test p-value: <0.0001*

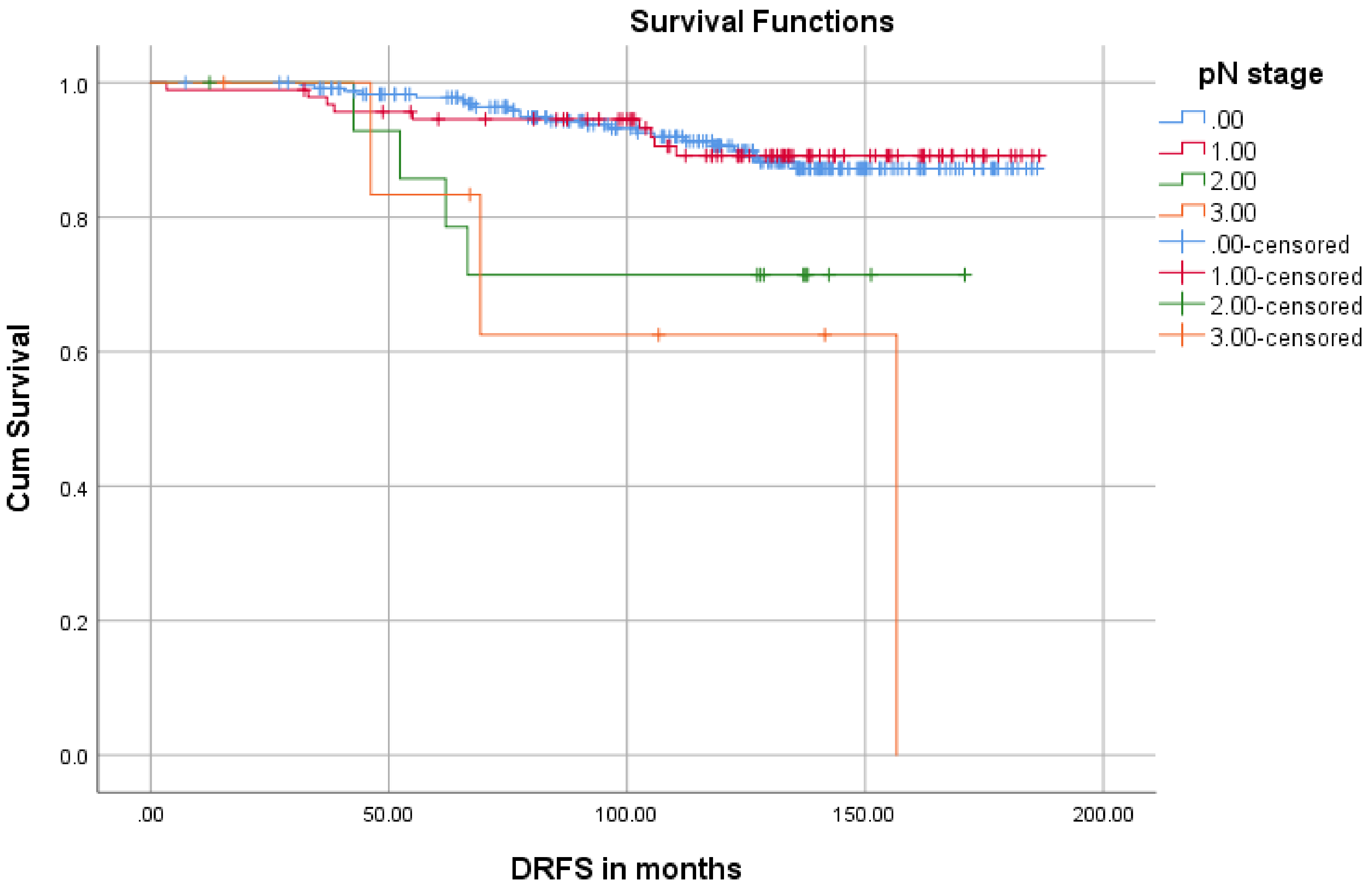

Log rank test p-value: 0.001*

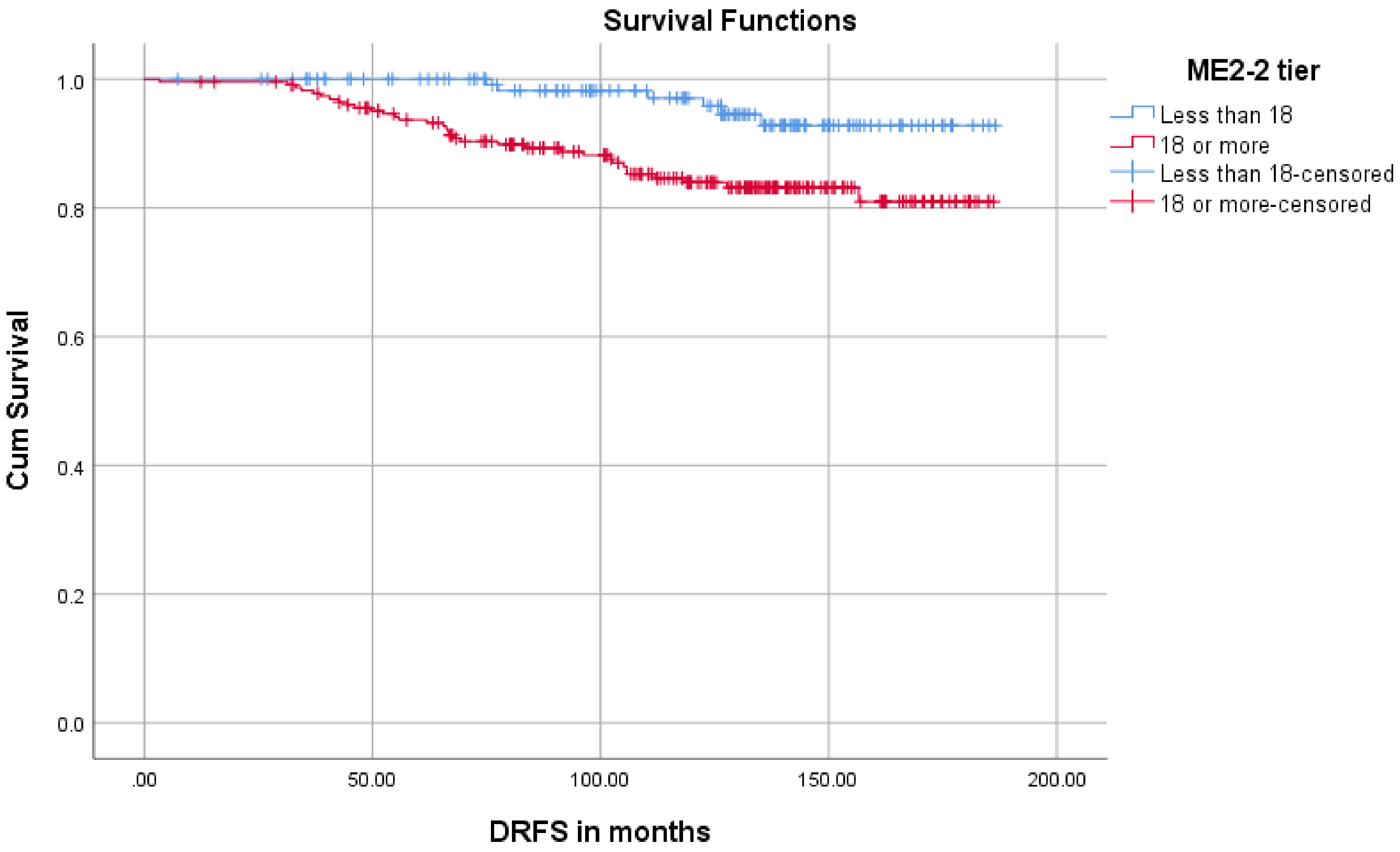

Log rank test p-value: 0.003*

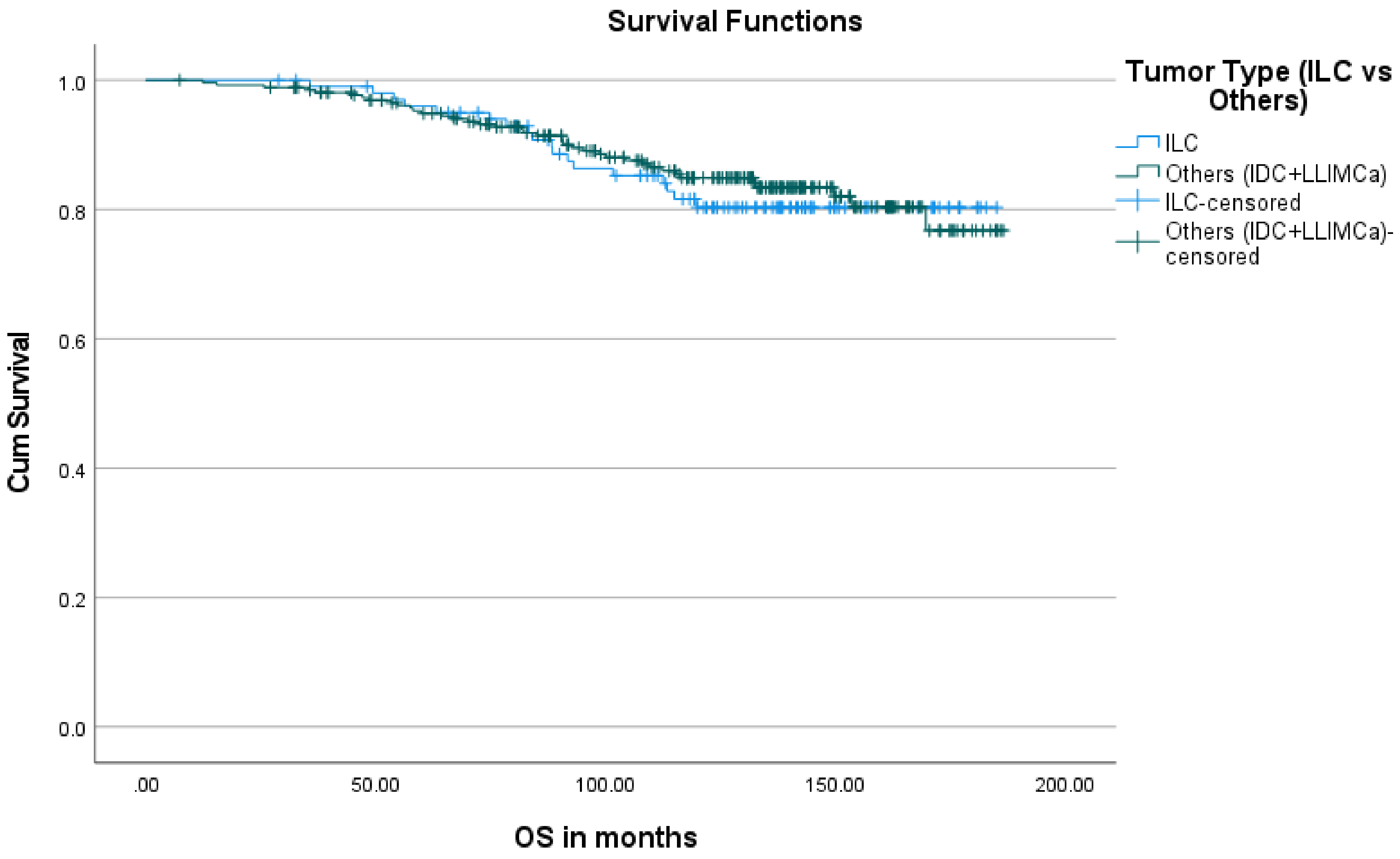

Log rank test p-value: 0.736

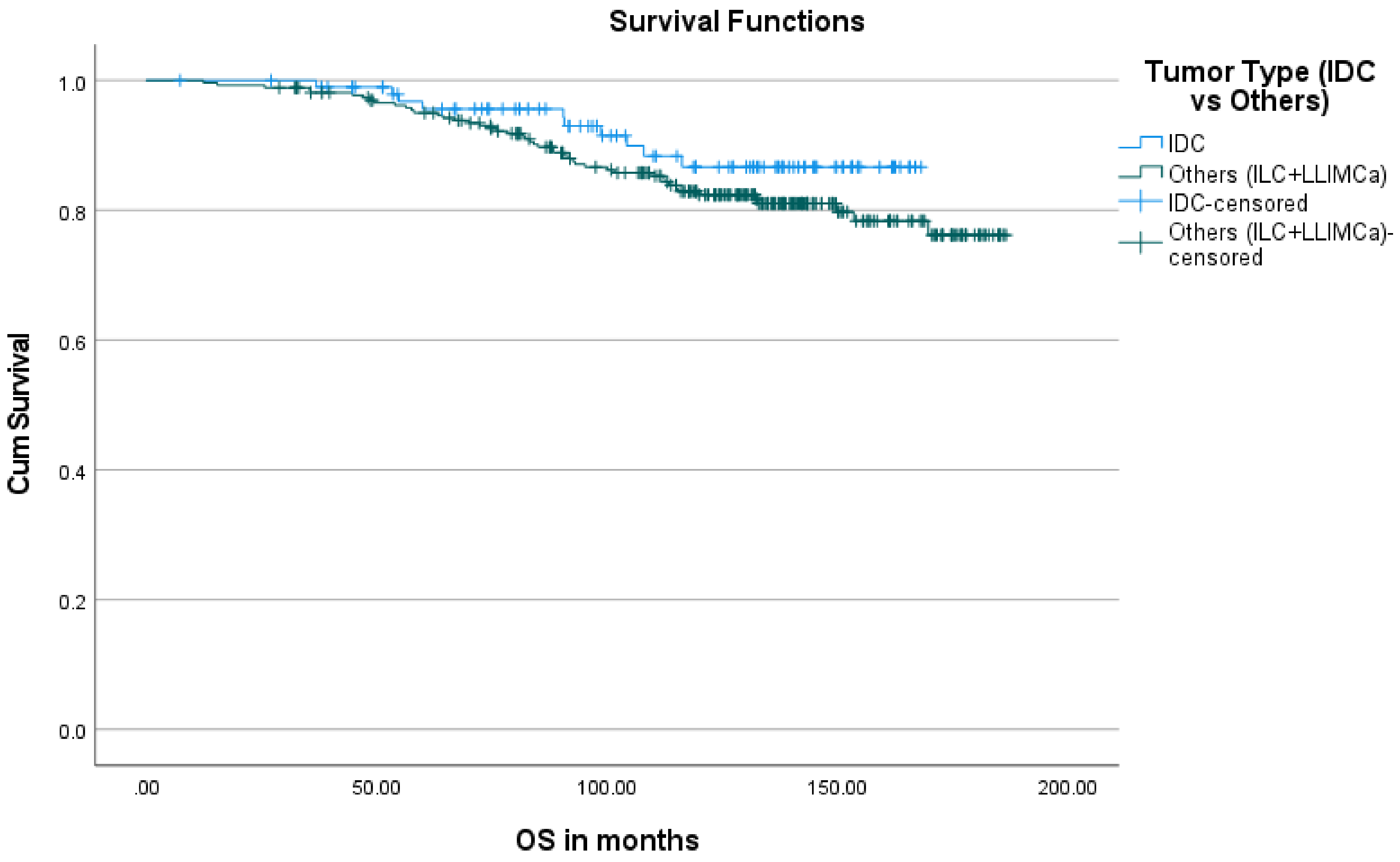

Log rank test p-value: 0.202

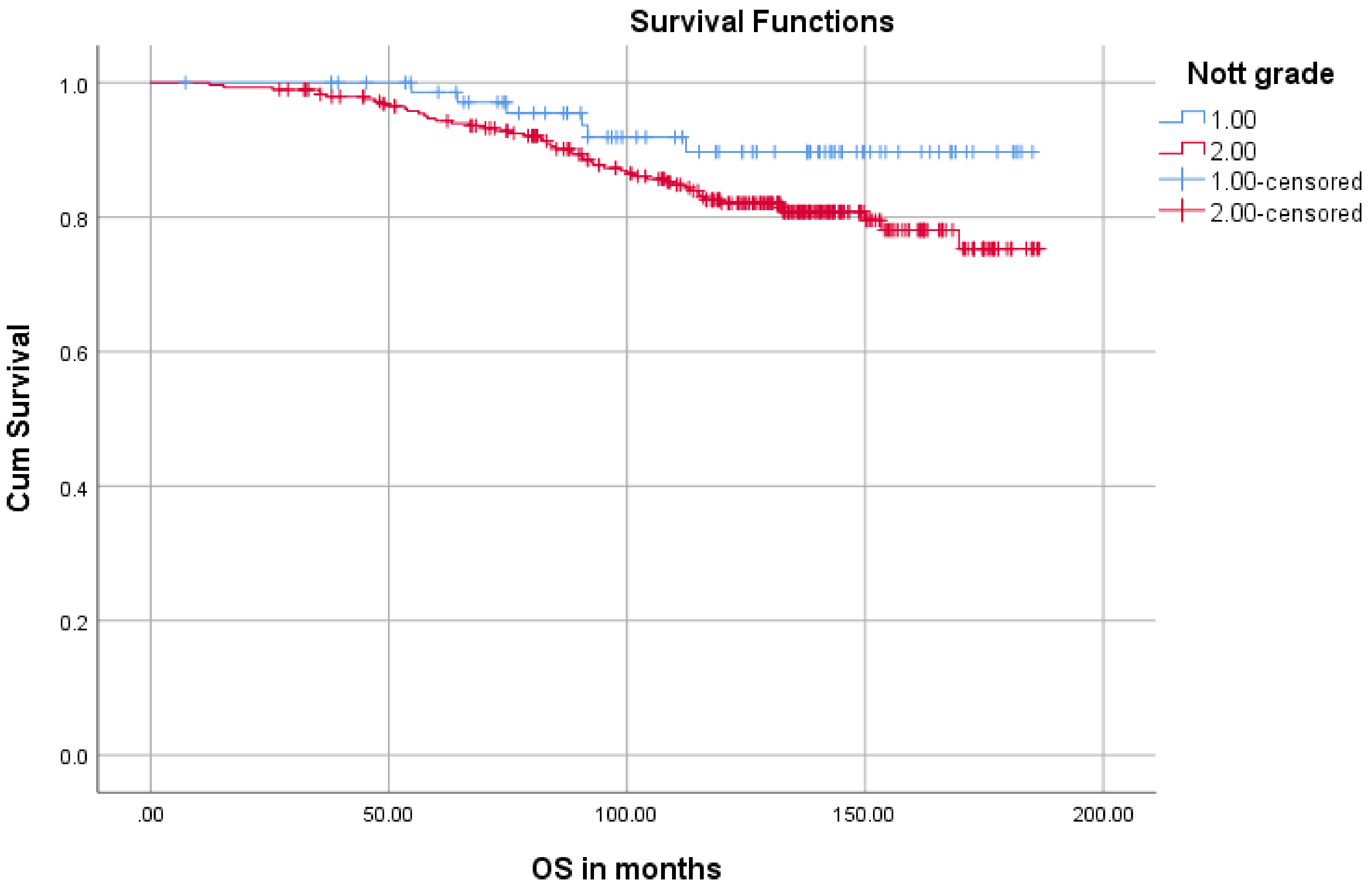

Log rank test p-value: 0.080

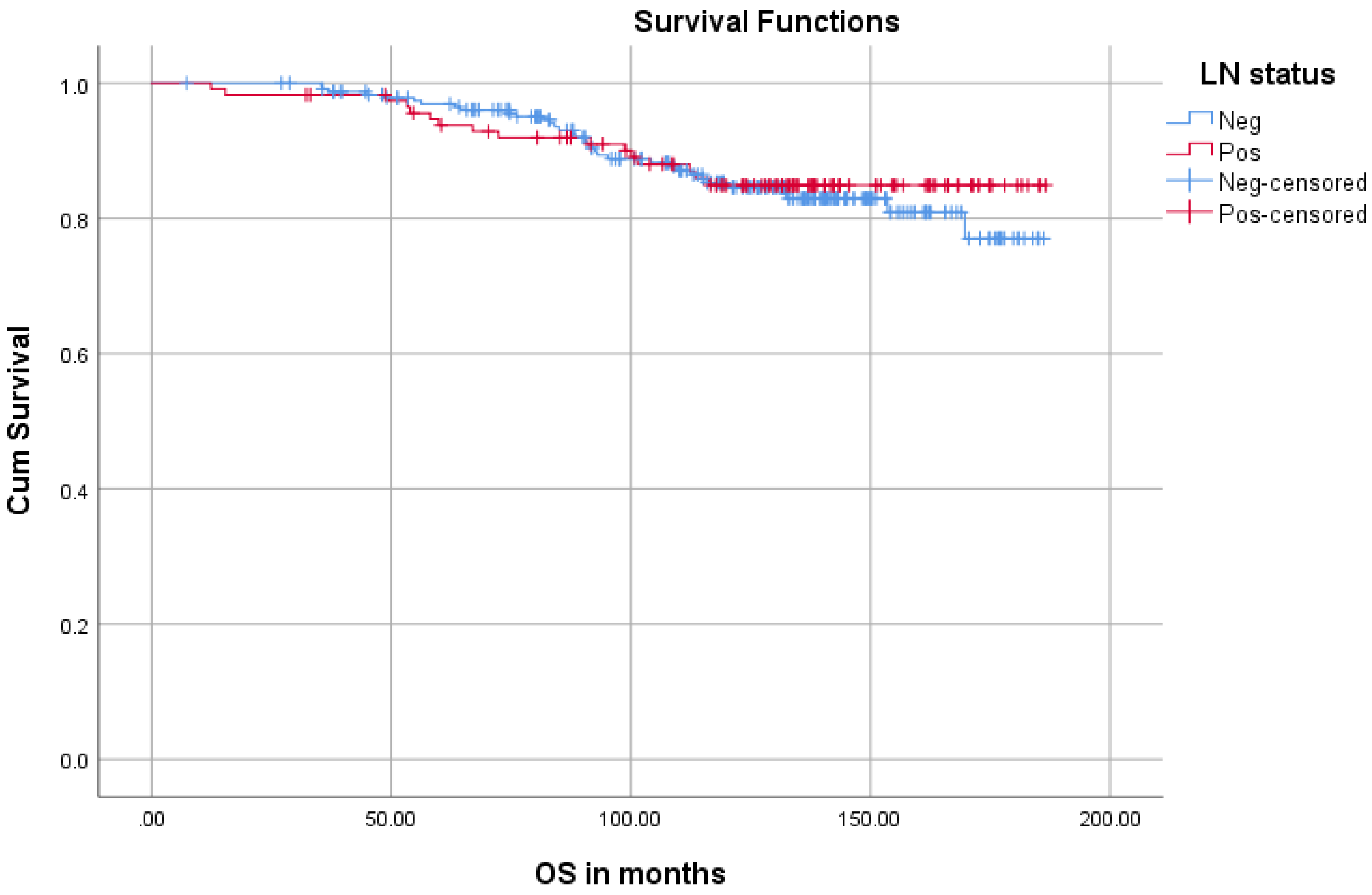

Log rank test p-value: 0.661

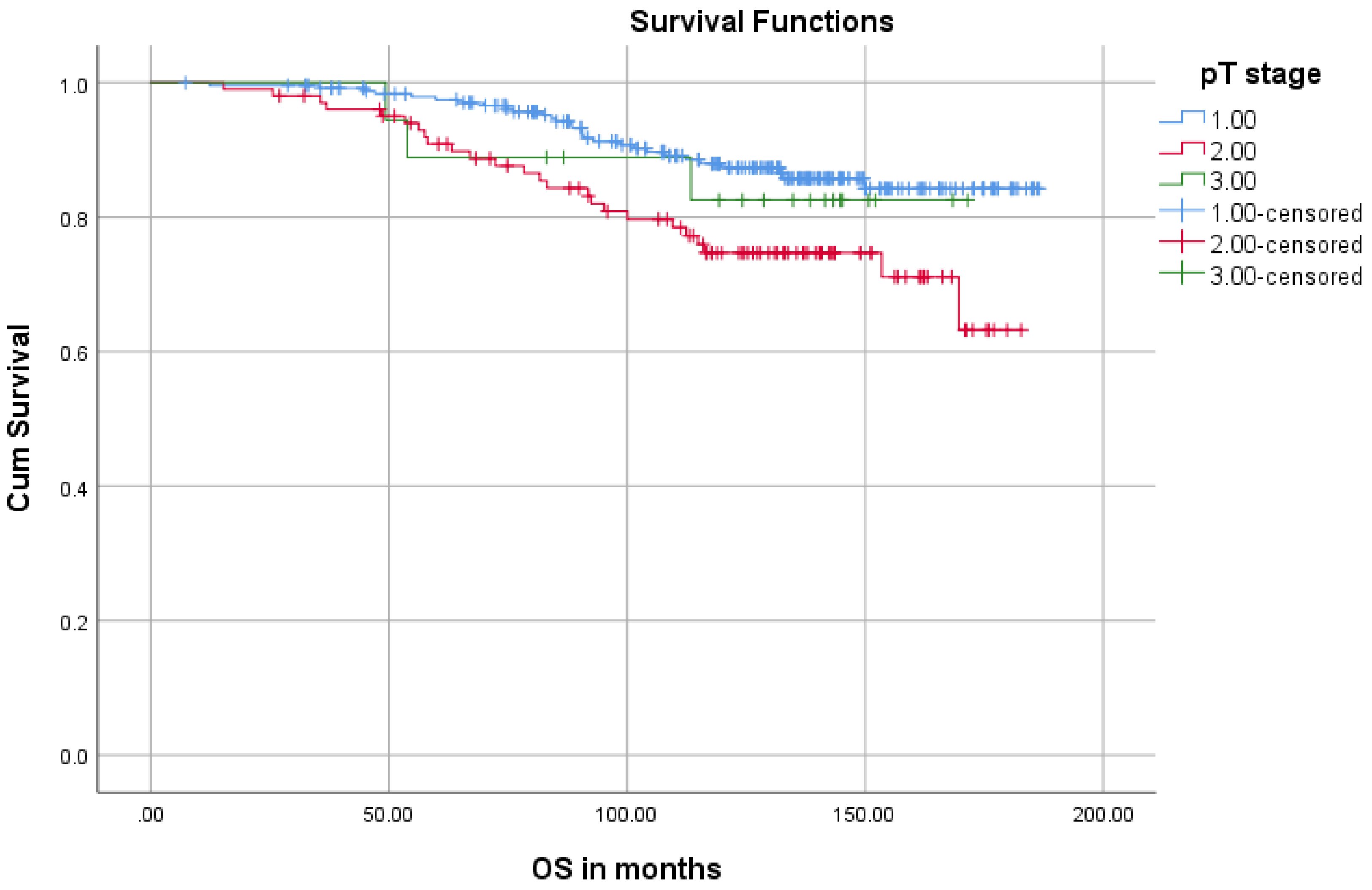

Log rank test p-value: 0.014*

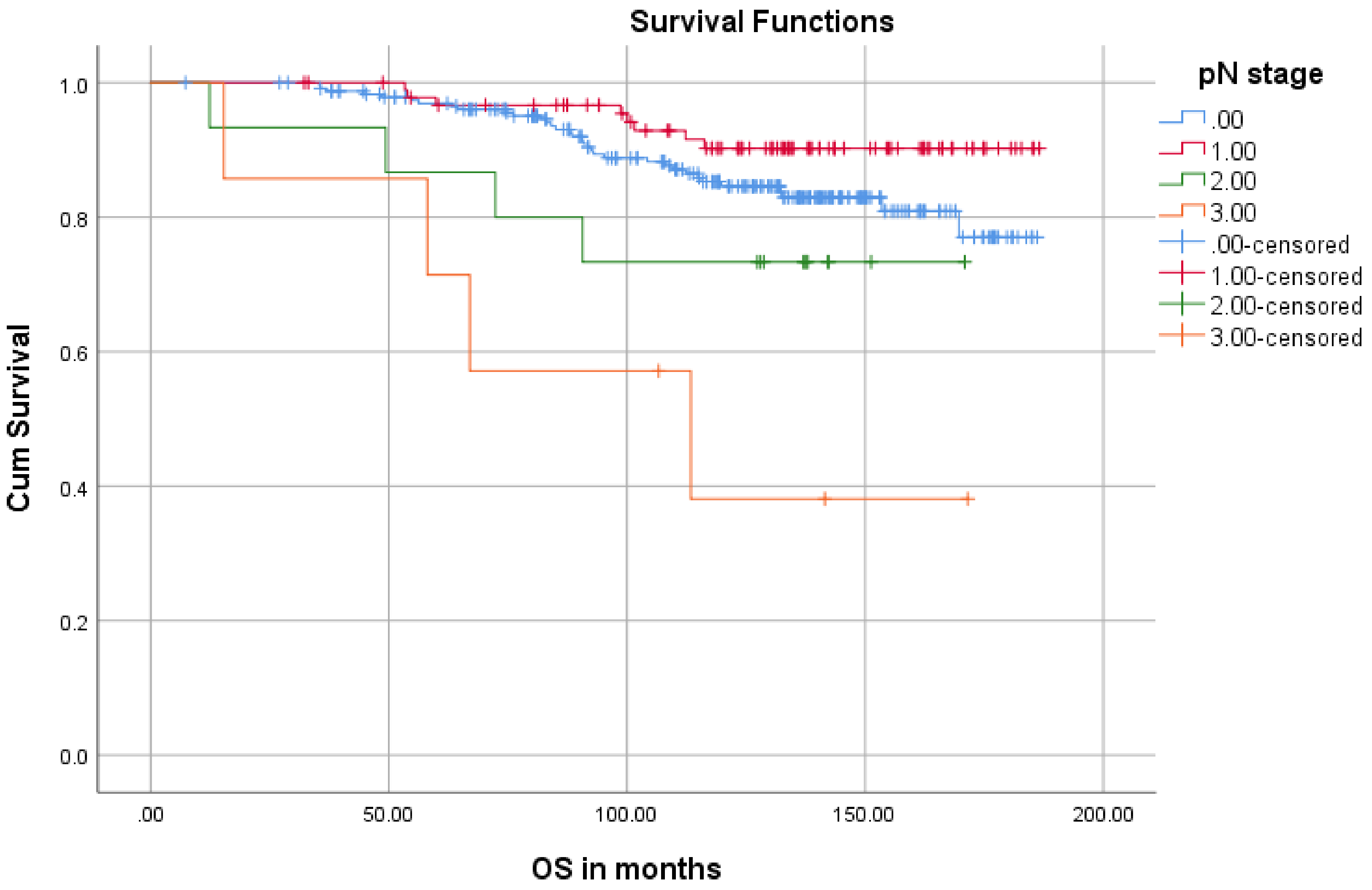

Log rank test p-value: <0.0001*

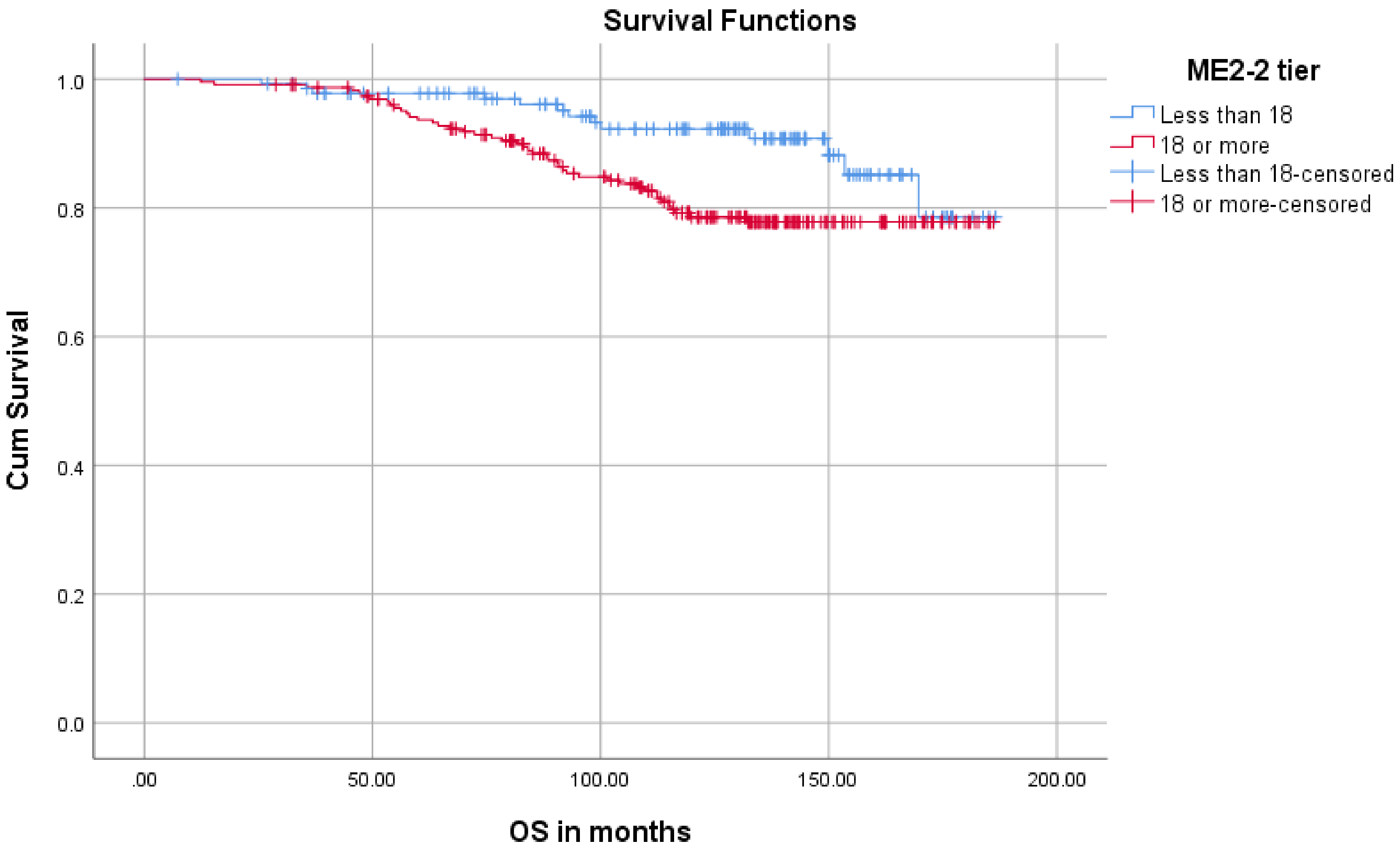

Log rank test p-value: 0.020*

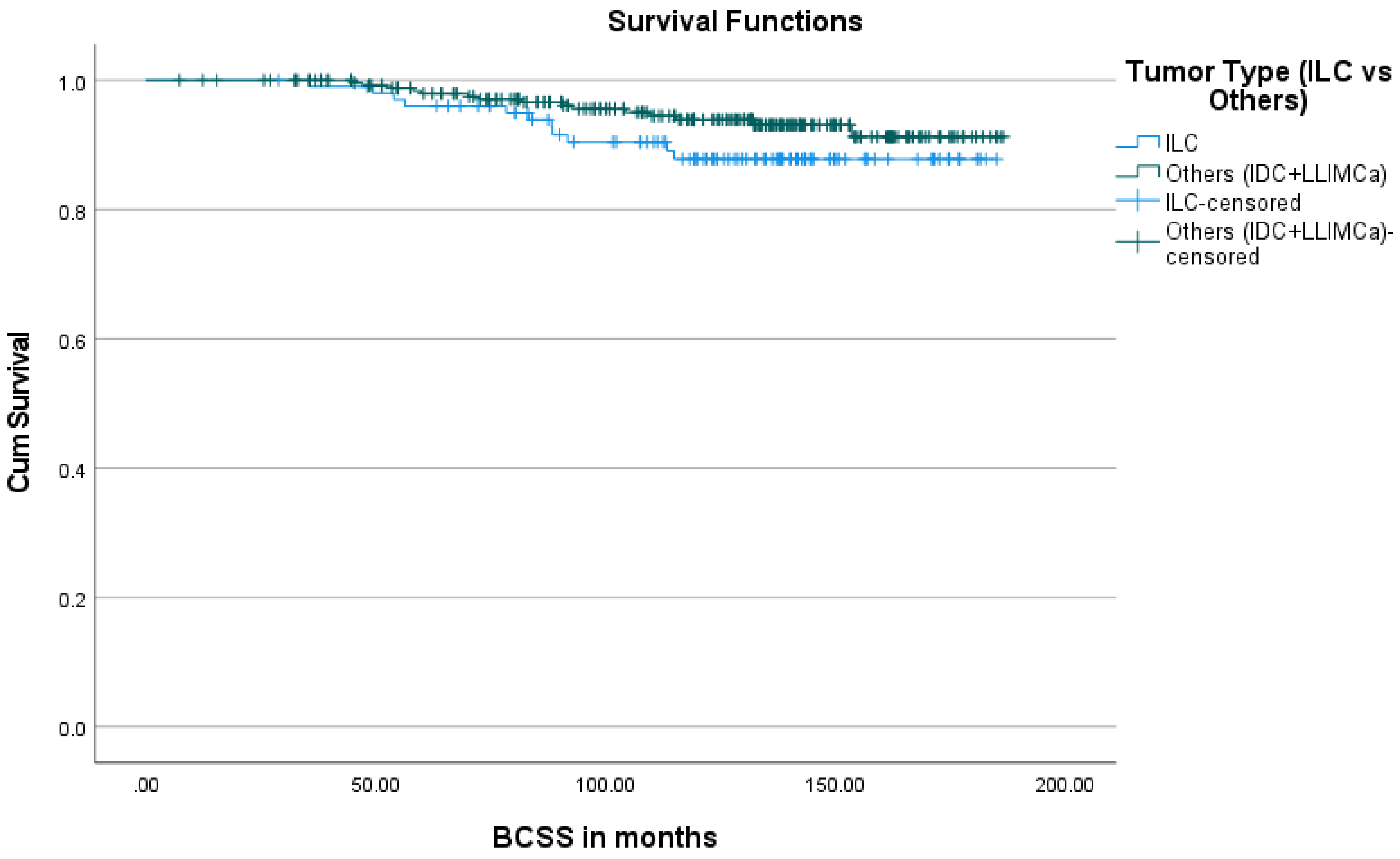

Log rank test p-value: 0.148

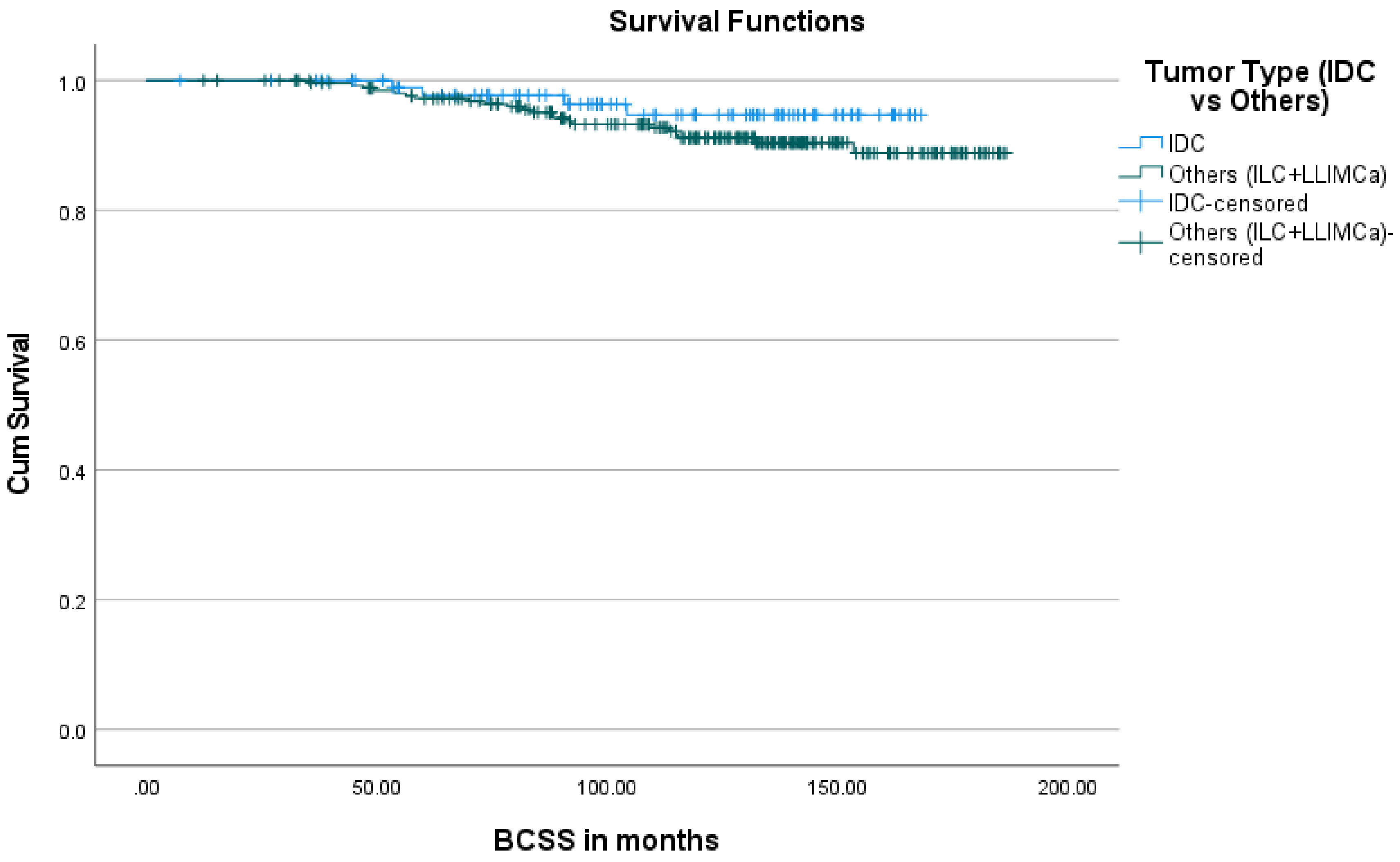

Log rank test p-value: 0.248

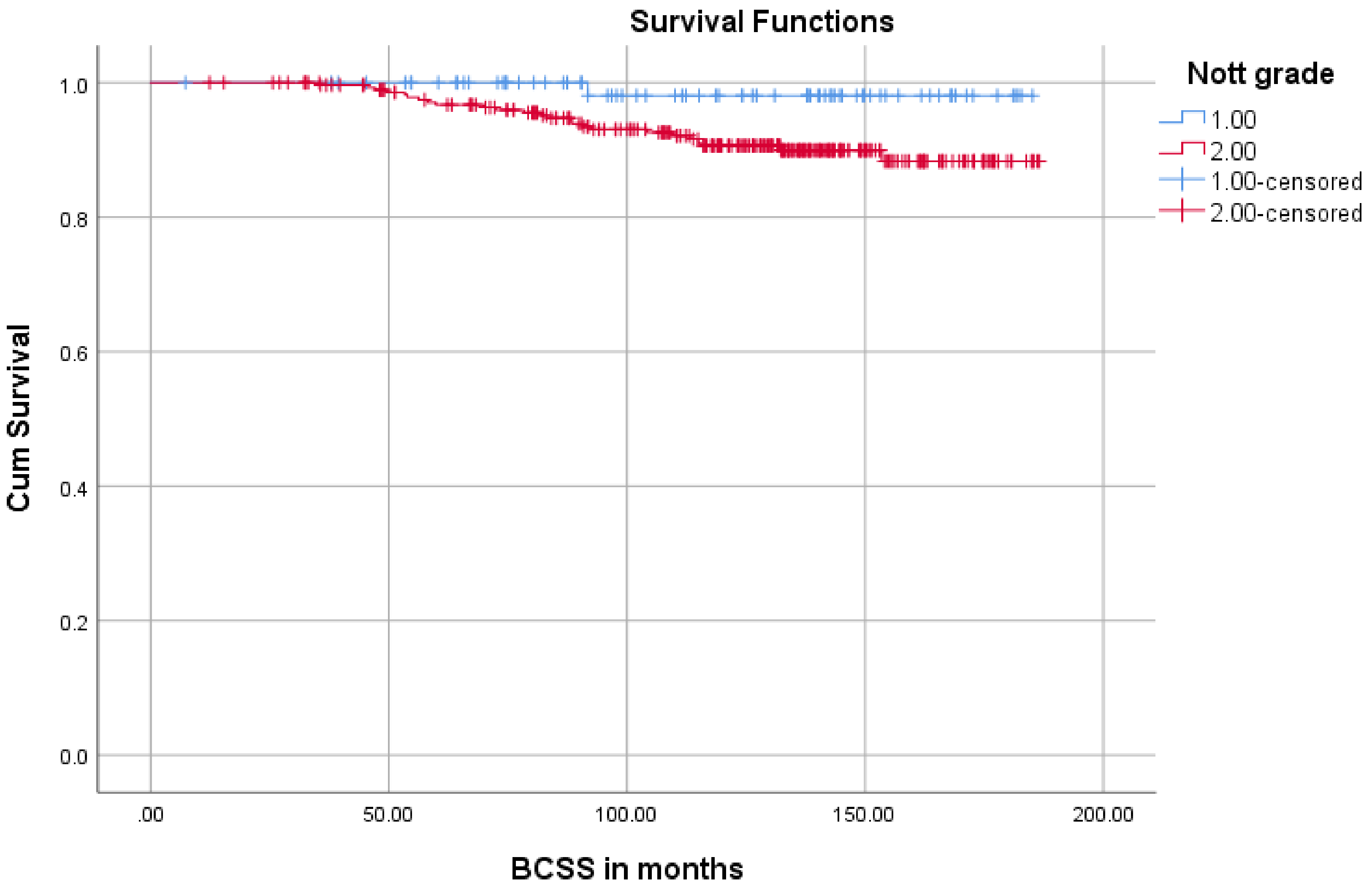

Log rank test p-value: 0.041*

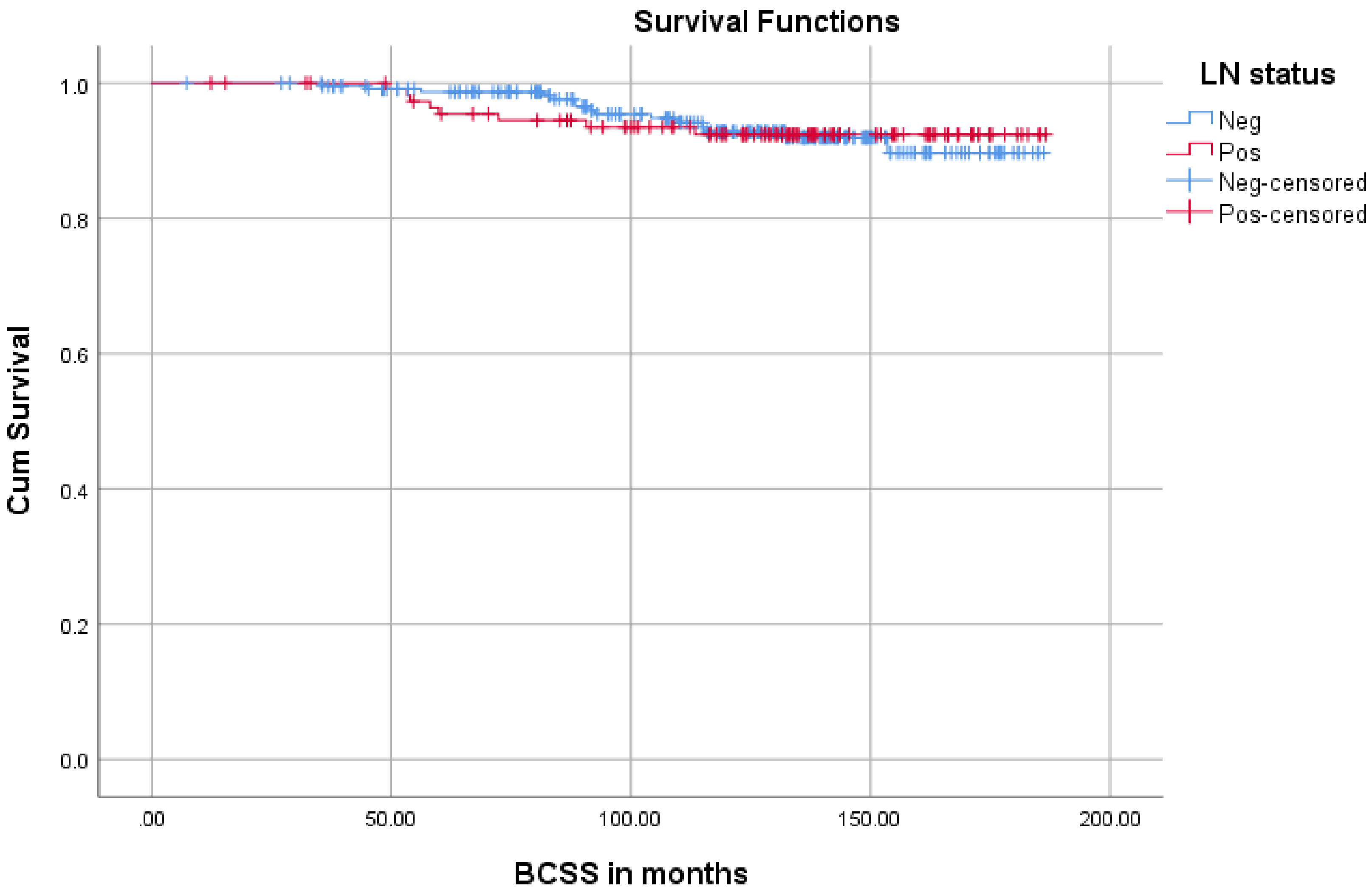

Log rank test p-value: 0.987

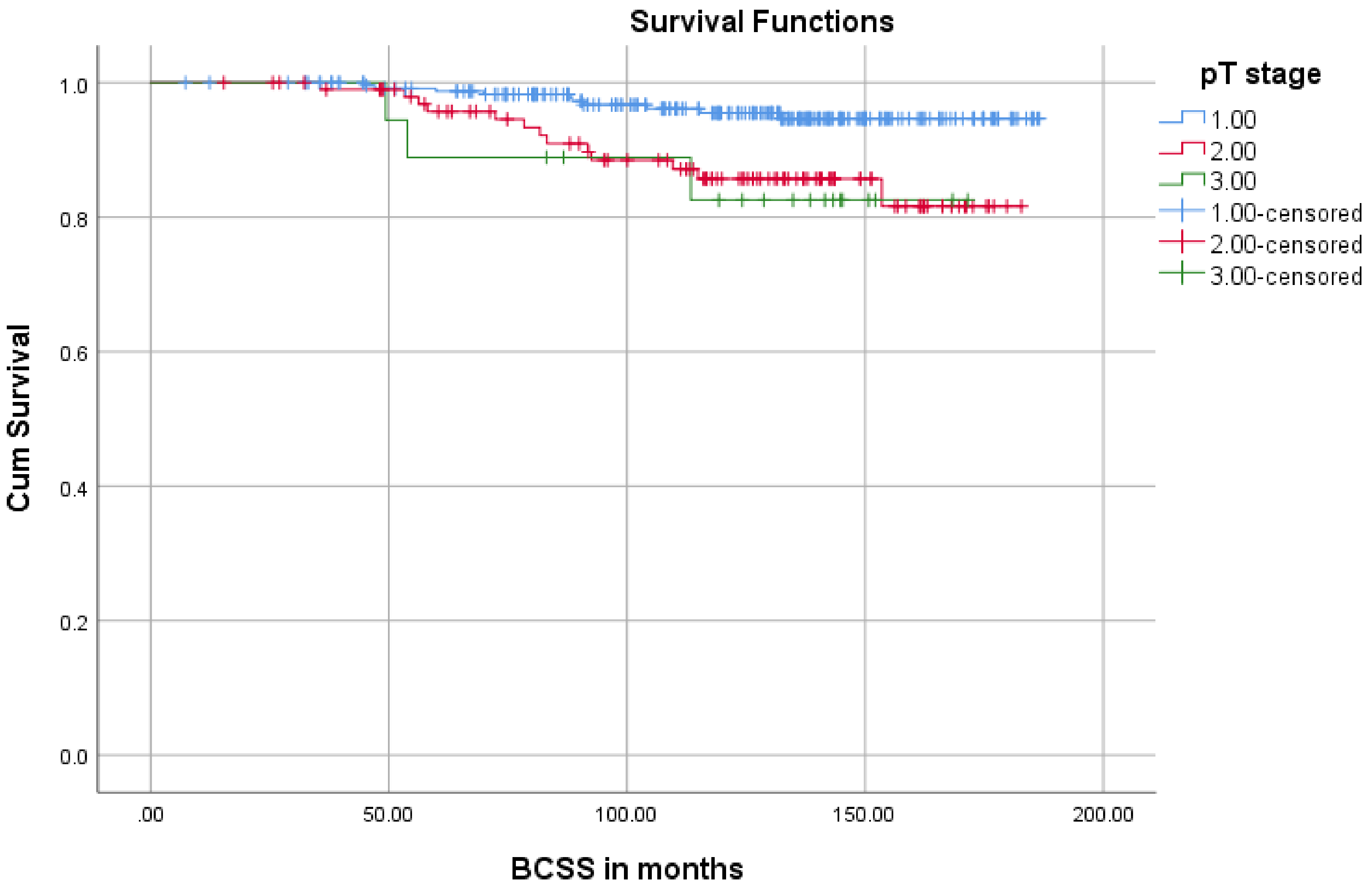

Log rank test p-value: 0.005*

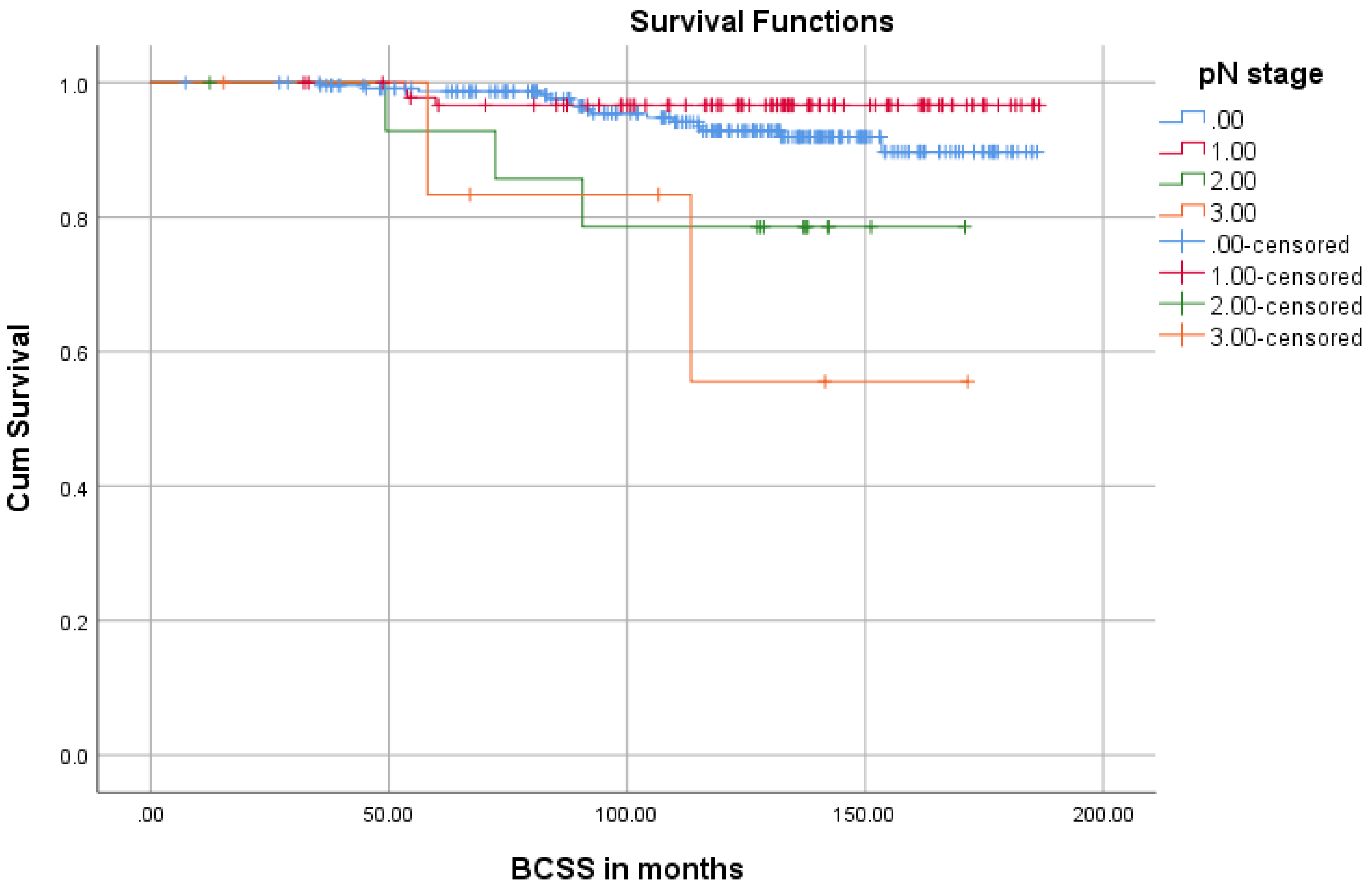

Log rank test p-value: 0.003*

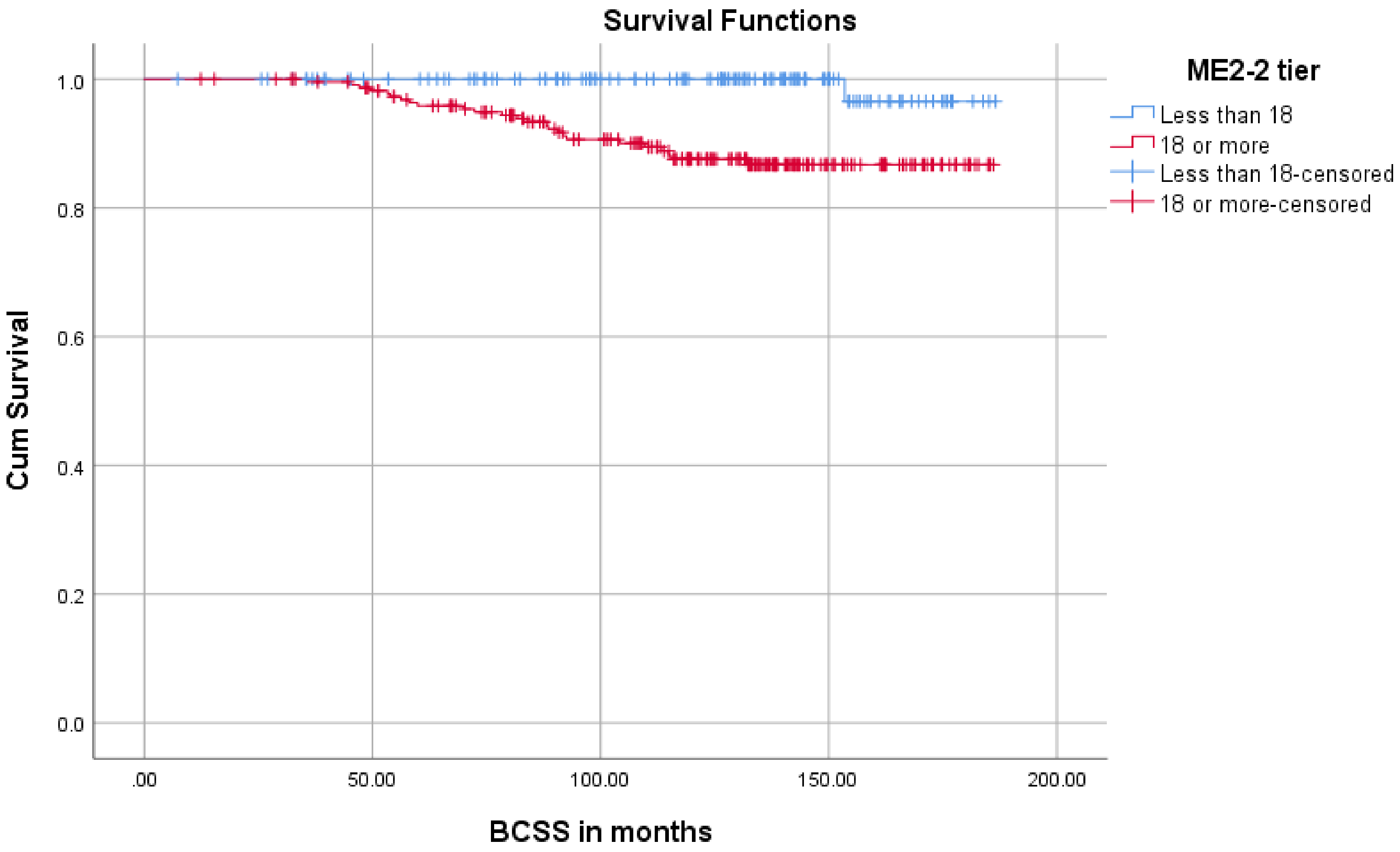

Log rank test p-value: <0.0001*

Supplementary Data

Cox proportional Hazard regression Analysis

The variables showing significant differences in survival by log rank test (Nottingham grade, pT, pN, and ME2) were included for multivariable Cox proportion hazard regression analysis. Since the survival difference for the pT stage was mostly seen between stage pT1 and others, it was used as a categorical variable (pT1 vs pT2+pT3) for Cox proportional hazard regression analysis. Similarly, for the pN stage, the survival difference was seen mostly between pN0/pN1 versus pN2/pN3 groups, it was also used as a categorical variable (pN0+pN1 vs pN2+pN3) for Cox proportional hazard regression analysis. pT stage and ME2 score categories showed statistically significant differences in RFS and DRFS on Cox proportion hazard regression analysis (see table below). pT stage, pN stage, and ME2 score categories showed statistically significant differences in OS and BCSS on Cox proportion hazard regression analysis (see table below).

Cox proportional hazard regression analysis

Variables	RFS	DRFS	OS	BCSS
Grade (1 vs 2)				
Regression coefficient	-1.229	-1.060	-0.232	-0.988
p-value	0.093	0.150	0.605	0.339
Hazard ratio	0.293	0.346	0.793	0.372
95% CI	0.070-1.229	0.082-1.466	0.330-1.907	0.049-2.818
pT (pT1 vs pT2+pT3)				
Regression coefficient	-0.819	-1.129	-0.604	-1.172
p-value	0.010*	0.001*	0.040*	0.009*
Hazard ratio	0.441	0.323	0.547	0.310
95% CI	0.236-0.825	0.163-0.641	0.307-0.973	0.128-0.749
pN (pN0+pN1 vs pN2+pN3)				
Regression coefficient	-0.784	-0.845	-0.861	-1.029
p-value	0.069	0.052	0.032*	0.049*
Hazard ratio	0.457	0.430	0.423	0.357
95% CI	0.196-1.063	0.183-1.009	0.192-0.930	0.128-0.996
ME2 score (<18 vs =18)				
Regression coefficient	-1.180	-1.037	-0.838	-2.458
p-value	0.008*	0.021*	0.020*	0.017*
Hazard ratio	0.307	0.355	0.432	0.086
95% CI	0.129-0.733	0.147-0.855	0.213-0.878	0.011-0.640

*Statistically significant. pT: Pathologic tumor stage; pN: Pathologic nodal stage; ME2: Magee Equation 2; CI: Confidence interval; RFS: Recurrence-free survival; DRFS: Distant recurrence-free survival; OS: Overall survival; BCSS: Breast cancer-specific survival.
